# Identification of key claudin genes associated with survival prognosis and diagnosis in colon cancer through integrated bioinformatic analysis

**DOI:** 10.3389/fgene.2023.1221815

**Published:** 2023-09-19

**Authors:** Rana A. Alghamdi, Maryam H. Al-Zahrani

**Affiliations:** ^1^ Department of Chemistry, Science and Arts College, King Abdulaziz University, Rabigh, Saudi Arabia; ^2^ Regenerative Medicine Unit, King Fahad Medical Research Centre, King Abdulaziz University, Jeddah, Saudi Arabia; ^3^ Biochemistry Department, Faculty of Science, King Abdulaziz University, Jeddah, Saudi Arabia

**Keywords:** claudin, bioinformatics methods, survival prognosis, integrated bioinformatic analysis, CLDN2, immune infiltrations, colon cncer

## Abstract

The claudin multigene family is associated with various aberrant physiological and cellular signaling pathways. However, the association of claudins with survival prognosis, signaling pathways, and diagnostic efficacy in colon cancer remains poorly understood.

**Methods:** Through the effective utilization of various bioinformatics methods, including differential gene expression analysis, gene set enrichment analysis protein-protein interaction (PPI) network analysis, survival analysis, single sample gene set enrichment analysis (ssGSEA), mutational variance analysis, and identifying receiver operating characteristic curve of claudins in The Cancer Genome Atlas colon adenocarcinoma (COAD).

**Results:** We found that: *CLDN2, CLDN1, CLDN14, CLDN16, CLDN18, CLDN9, CLDN12,* and *CLDN6* are elevated in COAD. In contrast, the *CLDN8, CLDN23, CLDN5, CLDN11, CLDN7,* and *CLDN15* are downregulated in COAD. By analyzing the public datasets GSE15781 and GSE50760 from NCBI-GEO (https://www.ncbi.nlm.nih.gov/geo/), we have confirmed that CLDN1, CLDN2, and CLDN14 are significantly upregulated and CLDN8 and CLDN23 are significantly downregulated in normal colon, colon adenocarcinoma tumor, and liver metastasis of colon adenocarcinoma tissues from human samples. Various claudins are mutated and found to be associated with diagnostic efficacy in COAD.

**Conclusion:** The claudin gene family is associated with prognosis, immune regulation, signaling pathway regulations, and diagnosis of COAD. These findings may provide new molecular insight into claudins in the treatment of colon cancer.

## 1 Introduction

### 1.1 Background

A family of multiple-gene transmembrane proteins called claudins has at least 27 members (Mineta et al., 2011; Zhu et al., 2019). For the purpose of signaling, claudin proteins are connected to tight junctions in cell-cell communication between the plasma membranes of two interacting cells (Krause et al., 2008). Claudins are related to various physiological functions, including paracellular ion pores, extracellular loops, ion permeability, cell polarity, affecting regulatory pathways, stabilizing the integrity of the epithelium, *etc.* (Krause et al., 2008; Markov et al., 2017). Claudins are crucially related to human diseases, including ovarian, breast, pancreatic, and prostate cancers (Singh et al., 2010; Kwon, 2013; Tabariès and Siegel, 2016). The deregulated expression level of claudins can be a modulator of carcinogenesis (Kwon, 2013; Tabariès and Siegel, 2016). Claudins work by regulating a number of processes in the development of cancer to the metastatic cascade, which is associated with the prediction of patient prognosis (Tabariès and Siegel, 2016). Likewise, they have recently been implicated in the epithelial-to-mesenchymal transition (EMT), the development of cancer stem cells, chemoresistance, and tumor recurrence (Kwon, 2013). Li (2021a) demonstrated that the aberrant expression of claudins is a potential target for cancer treatment because their abnormal expression is associated with neoplastic transformation (Ueda et al., 2007; Dhawan et al., 2011; Zhu et al., 2019; Zuo et al., 2020; Li, 2021a). Claudin-1, for instance, has been shown by Zuo et al. (2020) to be a significant predictive biomarker in colorectal cancer. The involvement of the claudin family in colitis-related colorectal cancer has been shown by Zhu et al. (2019). Claudin-1 protein has been linked to the development of colorectal cancer, according to research by Huo et al. (2009). The epidermal growth factor receptor (EGFR) may be transactivated by claudin-2 to promote colon cancer (Dhawan et al., 2011). The invasion and metastasis of colorectal cancer are significantly controlled by the reduced expression level of claudin-4 (Ueda et al., 2007). These findings show that claudins have a significant role in the invasion, metastasis, and prognosis of colon cancer.

In-depth bioinformatics analysis is presented in this article to show how variations in claudin expression levels affect the major malignant characteristics and immunology of colon adenocarcinomas. Furthermore, we explore how well claudins predict outcomes and aid in the diagnosis of colon cancer. We also discovered that the claudins are significantly involved in controlling the activation of carcinogenic pathways in COAD and that they are mutated.

## 2 Methods

During the academic years 2020–2021, this study was conducted in the computer labs of King Abdulaziz University. The study was approved by the research ethics committee (HA-02-J003) at the center of excellence in genomic medicine research (CEGMR). The ethical standards of the CEGMR were followed in the analysis of all the data used in this work.

### 2.1 Working Pipeline

**FIGURE 1:**
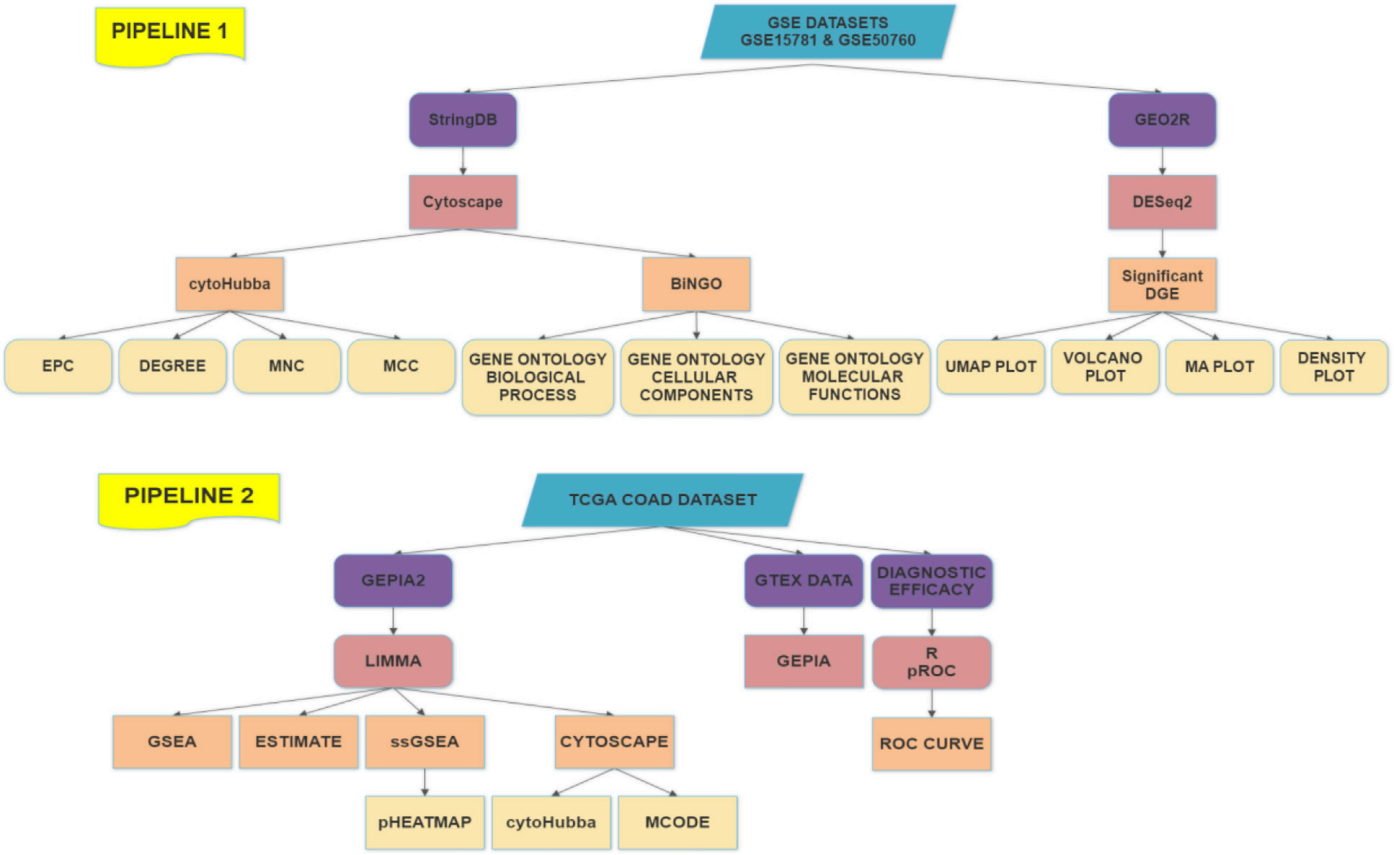
Working Pipeline of the analysis with two pipelines based on the data sources.

The working flowchart involves two pipelines based upon the data sources and the results have been combined effectively from all the processes followed and incorporated in this literature. The flowchart is presented to mark different aspects of the combinatorial study in Figure 1.

### 2.2 Identification of differentially expressed genes from public datasets

We downloaded The Cancer Genome Atlas (TCGA) COAD cohort (https://portal.gdc.cancer.gov/) and normalized the data into log2-base transformation. For analyzing the survival differences between two groups of patients in the TCGA COAD dataset (https://portal.gdc.cancer.gov/), we used the Expression Profiling Interactive Analysis (GEPIA) (Tang et al., 2017) tool (http://gepia2.cancer-pku.cn/#index).

The R package “limma” was employed for identifying the significant differentially expressed genes (DEGs) between COAD (*n* = 287) and normal samples (*n* = 41) (Ritchie et al., 2015). We identified the DEGs with a threshold absolute value of Log_2_FC > 0.50 and adjusted *p*-value ≤ 0.05. In addition, we checked the differential expression of claudins in TCGA COAD with combined GTEx normal data (adjusted *p*-value ≤ 0.05) by using Gene Expression Profiling Interactive Analysis (GEPIA) (Tang et al., 2017).

We used NCBI-GEO’s public datasets, platforms GPL2986 for GSE15781 (N: 10, T: 13), and platforms GPL11154 for GSE50760 (N: 18, T: 18, and M: 18) to analyze the expression of Claudin genes as well as significant molecular hallmarks in normal colon (N), colon adenocarcinoma tumor (T), and liver metastasis of colon adenocarcinoma tissues (M) from human samples using an interactive web application called GEO2R. It was employed to contrast two or more groups of Samples from a GEO Series in order to find genes that exhibit differential expression under various experimental circumstances. In order to view differentially expressed genes and evaluate the quality of the data set, the results are given as a table of genes arranged by *p*-value and as a collection of graphic plots. The R programming language is the foundation of the open-source software project known as Bioconductor, which offers tools for the study of high-throughput genetic data. Numerous R packages from the Bioconductor project are used by GEO2R. Input NCBI-generated raw count matrices are used by GEO2R to perform differential expression analysis using DESeq2. It is suitable for both large observational studies and small studies with few repetitions because it employs negative binomial generalized linear models and has features that allow consistent performance throughout a wide variety of data types.

**FIGURE 2:**
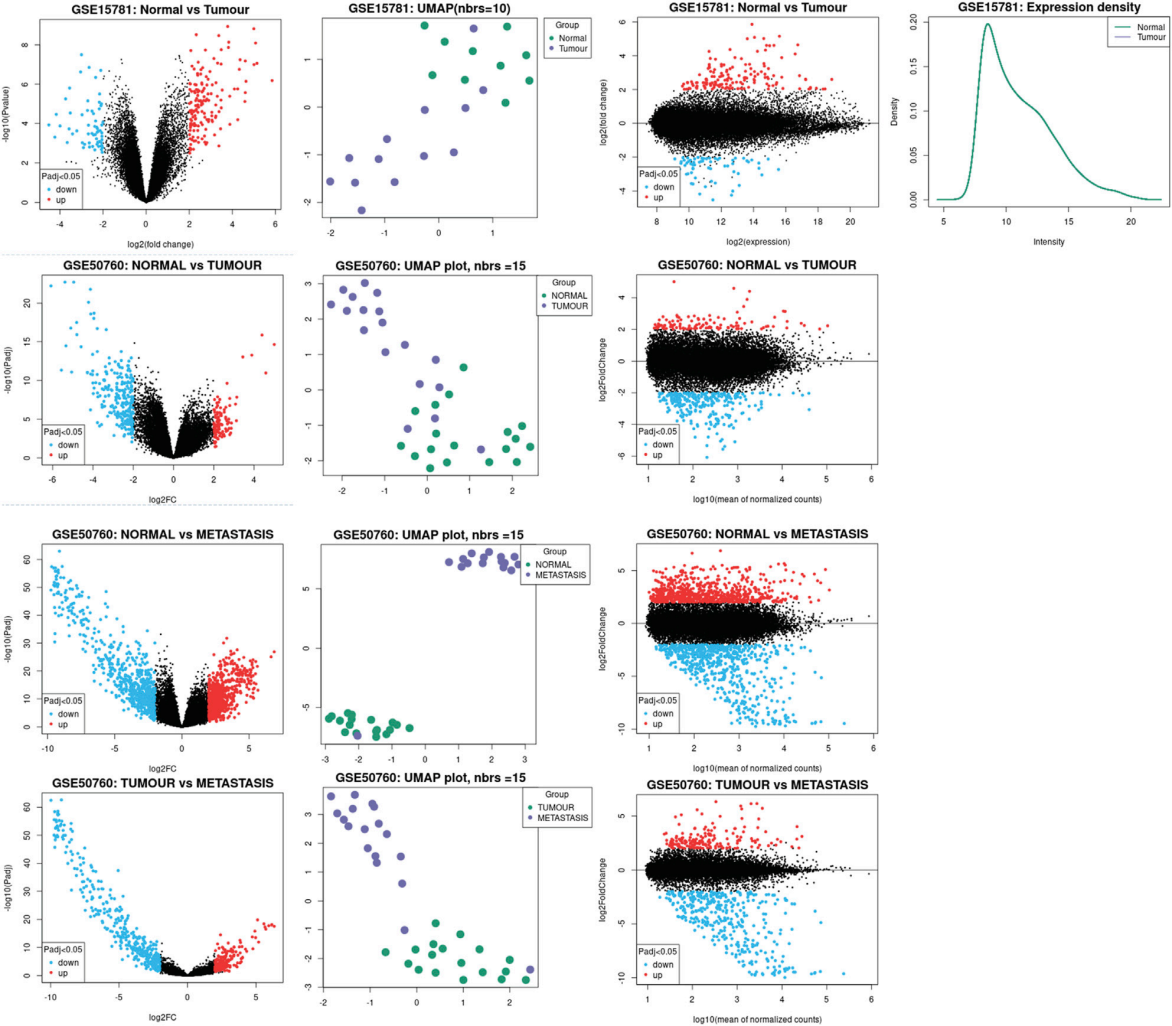
Illustration of the data analysis process. Significant Claudins were inferred using the DESeq2 package of GEO2R. UMAP plot was used for data visualization, the scatterplot displays statistical significance (*p*-value) vs magnitude of change (fold change) along with the volcano plot.

We inferred the significant claudins from the differential gene expression done using the DESeq2 package of GEO2R. By condensing data into two dimensions, a UMAP plot was used for data visualization. The umap package now provides utility functions to make visualizing UMAP data straightforward and offer a variety of ways to analyze and diagnose the findings because this is such a prevalent use case. We used a scatterplot that displays statistical significance (*p*-value) vs. magnitude of change (fold change) is known as a volcano plot. It makes it possible to quickly visually identify genes that have substantial statistical fold changes. These genes could be the ones with the most biological impact. A density plot was shown to give a depiction of a numeric variable’s distribution that displays the probability density function of the variable using a kernel density estimate and an adaptation of a Bland-Altman plot known as an MA plot is used in computational biology to visualize genetic data. By converting the data to M and A scales and then showing these values, the figure illustrates the variations between measurements made in two samples. We have illustrated the data analysis in Figure 2.

### 2.3 Gene-set enrichment analysis

Using the GSEA (Subramanian et al., 2005), we conducted gene-set enrichment analysis on the DEGs. For the GSEA tool to determine Gene Ontology (GO) and pathways, all 23 claudins were entered. The pathways from the Reactome (Jassal et al., 2020) and Kyoto Encyclopedia of Genes and Genomes (KEGG) (Kanehisa et al., 2017) databases that are significantly linked to claudins were found. When choosing the GO keywords and pathways, the *p*-value of 0.05 was regarded as a significant cut-off.

### 2.4 Construction of protein-protein interaction (PPI) network of claudins and identifying modules

The PPI network was created using the STRING tool in order to comprehend the relationships between all claudins (Szklarczyk et al., 2019). We utilized the Cytoscape plug-in program cytoHubba to determine the rank of hub genes (Chin et al., 2014). Based on the extent of interactions with nearby genes, the rank of the genes was determined. For determining the PPI of claudins, we decided that an interaction score of at least 0.40 was necessary. Using the program Cytoscape 3.6.1, we can see the PPI networks (Shannon et al., 2003). Utilizing the Cytoscape plug-in molecular complex discovery (MCODE) tool from the initial PPI network, we were able to identify the crucial modules (Bader and Hogue, 2003). We identified the significant modules on the basis of MCODE score and node number. The parameters that we used to determine the MCODE threshold were: MCODE score>5.0, Node Score Cut-off: 0.2, Haircut: true, K-Core: 2, and maximum depth from Seed: 100.

### 2.5 Survival analysis of claudins by using the GEPIA tool

The informative and frequently cited GEPIA (Gene Expression Profiling Interactive Analysis) resource has been used to build various analyses utilizing tumor and normal samples from the TCGA and GTEx databases. The GEPIA tool was used to compare the colon cancer patients’ overall survival (OS) and disease-free survival (DFS). The survival disparities between the high expression group and low expression group were displayed using Kaplan-Meier survival curves (High expression group > median > Low expression group). Using GEPIA (Tang et al., 2017) databases, the survival significance of each differentially expressed claudin in the TCGA COAD cohort was examined. When comparing the survival between the two groups, a Cox regression *p*-value of 0.05 was deemed significant.

### 2.6 ESTIMATE algorithmic for quantifying immune score and stromal score

ESTIMATE is an algorithmic tool based on the R package for predicting tumor purity, Immune Score (predicting the infiltrations of immune cells), and Stromal Score (predicting the infiltrations of stromal cells) which uses the gene expression profiles of 141 immune genes and 141 stromal genes (Yoshihara et al., 2013). The presence of infiltrated immune cells and stromal cells in tumor tissues was calculated using related gene expression matrix data, represented by Immune Score and Stromal Score, respectively (Yoshihara et al., 2013). Then, the correlations of key genes with immune scores and stromal scores were calculated. The threshold value of correlation is R > 0.20, and the *p*-value is not less than 0.001 (Spearman’s correlation test).

### 2.7 Single sample gene set enrichment analysis (ssGSEA)

One of the extension packages of GSEA, single-sample gene-set enrichment analysis (ssGSEA) was used to identify the enrichment scores of immune cells for each pairing of a sample and gene set in the tumor samples (Hänzelmann et al., 2013). The marker gene set for immune signatures, biological processes, and cancer-associated pathways was collected, and each gene set was employed to quantify the ssGSEA scores of specific immune signatures (Tirosh et al., 2016; Aran et al., 2017; Davoli et al., 2017; Liu et al., 2018). The ssGSEA score was identified with various immune stimulatory and inhibitory signatures, including B cells, cancer-associated fibroblasts (CAFs), CD4 regulatory T cells, CD8 T cells, cytolytic activity, endothelial cells, immune checkpoint genes, M2 macrophages, macrophages, myeloid-derived suppressor cells (MDSCs), neutrophils, natural killer (NK) cells, plasmacytoid dendritic cells (pDC), T cell activation, T cell exhaustion, tumor-associated macrophages (TAM), T follicular helper cells (Tfh), T-helper 17 (Th17), tumor infiltrating lymphocytes TILs, and regulatory T cells (Tregs). Then, we identified the ssGSEA score of angiogenesis, apical junction, apoptosis, epithelial-mesenchymal transition (EMT), hypoxia, proliferation, and stemness. Moreover, the identified ssGSEA score of cancer-associated pathways (Chen et al., 2020) included cell adhesion molecules (CAMs), extracellular matrix (ECM) receptor interaction, epidermal growth factor receptor (ERBB) signaling pathway, focal adhesion, gap junction, leukocyte transendothelial migration, mitogen-activated protein kinase (MAPK) signaling pathway, mammalian target of rapamycin (MTOR) signaling, Notch signaling, pathways in cancer, transforming growth factor (TGF) beta signaling, tight junction, vascular endothelial growth factor (VEGF) signaling pathway, and Wingless/Integrated (Wnt) signaling. All of the gene sets are attached in the Supplementary Table S1.

### 2.8 Diagnostic efficacy evaluation of differentially expressed claudins in the COAD

To assess diagnostic values of the prognostic genes, the receiver operating characteristic (ROC) curve was plotted and the area under the ROC curve (AUC) was calculated using the “pROC” R package (Robin et al., 2011) to evaluate the capability of distinguishing COAD and normal samples. We selected the expression level of these claudins members in tumor samples as cases in comparison with the normal sample as control. The greater AUC value of individual genes indicated the differences between tumor and normal samples, and the key gene of AUC>0.5 in the CAFs datasets was defined as a diagnostic efficiency of the gene (Yang et al., 2018).

### 2.9 Statistical analysis

R software version 4.0.1 was used for all statistical analyses. In the Log-rank test, a *p*-value <0.05 was considered statistically significant for survival analysis. In order to investigate the correlation of genes, Spearman’s correlation between the ssGSEA scores and specific genes was performed (*p*-value <0.001). The Pearson correlation test was used to identify the correlation between the two genes (*p*-value ≤0.05). The pheatmap package (version 1.0.8, https://cran.r-project.org/web/packages/pheatmap/index.html) in R (Version 4.1.0) was utilized to draw the heatmap. The R package “ggplot2” was implemented for preparing the graphical representation of the Heatmap and the correlation graph.

## 3 Results

### 3.1 Identifying the differentially expressed claudins gene members in the COAD

**FIGURE 3:**
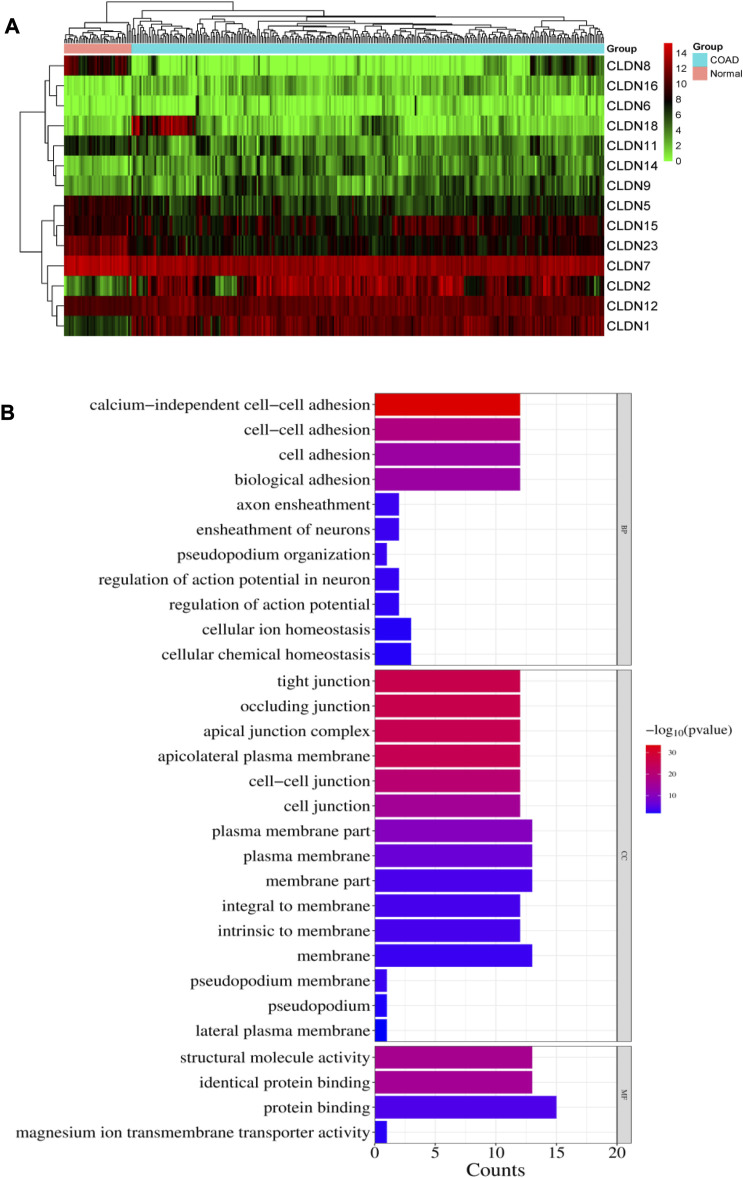
**(A)**. Heatmap of the differentially expressed 14 claudins in the TCGA COAD samples. **(B)**. illustrates the significance of the biological pathways, cellular components, and molecular functions identified through BiNGO analysis of the important Claudin genes from GSE15781 and GSE50760 studies.

We investigated the differential expression analysis of Claudins gene members in the COAD relative to the normal samples (Table 1). We found that the expression level of *CLDN2, CLDN1, CLDN14, CLDN16, CLDN18, CLDN9, CLDN12,* and *CLDN6* are elevated in the COAD. In contrast, the expression level of *CLDN8, CLDN23, CLDN5, CLDN11, CLDN7,* and *CLDN15* is downregulated in the COAD. The Heatmap of the expression value of differentially expressed genes is shown in Figure 3A. When compared to TCGA normal samples, the expression of other claudins is not changed in the TCGA COAD samples. Additionally, using combined GTEx and TCGA normal data, we examined the differential expression of these claudins in the TCGA tumor samples. We found that *CLDN2, CLDN1, CLDN14,* and *CLDN12 are* consistently upregulated after increasing the normal samples and *CLDN8, CLDN5,* and *CLDN11* are consistently downregulated at the same condition (Supplementary Figure S1). However, *CLDN23* are *CLDN7* upregulated when adding the GTEx normal data. Using public datasets GSE15781 and GSE50760 from NCBI-GEO (https://www.ncbi.nlm.nih.gov/geo/), we validated CLDN1, CLDN2, and CLDN14 to be significantly upregulated and CLDN8 and CLDN23 to be significantly downregulated in normal colon *versus* tumor tissues from human samples.

**TABLE 1:** The claudins gene members are differentially expressed in the TCGA COAD.

Entrez id	Regulatory status	log2FC	Average expression	*p*-value	Adjusted *p*-value	Symbols	Name of the genes
9075	Upregulated	5.96	9.85	1.07E-34	4.55E-34	CLDN2	Claudin 2
9076		4.76	10.21	1.00E-76	1.70E-75	CLDN1	Claudin 1
23562		2.23	3.71	1.03E-16	2.19E-16	CLDN14	Claudin 14
10686		1.69	2.03	5.27E-16	9.95E-16	CLDN16	Claudin 16
51208		1.61	2.85	4.16E-03	5.44E-03	CLDN18	Claudin 18
9080		1.49	4.26	1.20E-07	1.86E-07	CLDN9	Claudin 9
9069		0.80	10.84	2.86E-14	4.87E-14	CLDN12	Claudin 12
9074		0.57	0.82	1.92E-03	2.71E-03	CLDN6	Claudin 6
24146	Downregulated	−0.52	8.67	4.11E-02	4.99E-02	CLDN15	Claudin 15
1366		−1.27	12.72	2.19E-28	6.20E-28	CLDN7	Claudin 7
5010		−2.60	4.39	2.60E-20	6.31E-20	CLDN11	Claudin 11
7122		−2.71	6.88	1.06E-30	3.59E-30	CLDN5	Claudin 5
137075		−3.32	8.21	4.09E-58	2.32E-57	CLDN23	Claudin 23
9073		−7.48	2.44	2.51E-58	2.13E-57	CLDN8	Claudin 8

The public datasets are obtained from NCBI-GEO (Gene Expression Omnibus) and are analyzed using the standard protocol of Geo2R. The datasets are taken from human tissue samples of healthy colon as control, tumor and metastasis.

The investigation let us critically cross-examine the data analysis generated by TCGA-COAD and provided us with substantial molecular signatures for colon cancer or colorectal cancer. We found that between normal and tumor samples, CLDN8 and CLDN23 were downregulated and CLDN1 and CLDN2 were upregulated, respectively. However, when compared to samples of tumors as opposed to metastases, we found CLDN14 to be significantly enriched. Table 2 contains statistics for the aforementioned data as well as additional significant molecular markers discovered through analysis of the external datasets GSE15781 and GSE50760.

**TABLE 2:** The claudins gene members are significantly differentially expressed in the public datasets: GSE15781 and GSE50760.

SIGNIFICANT_CLDN		CLDN_DOWNregulated	CLDN_UPregulated
CLDN1	CLDN11	CLDN8	CLDN1
CLDN2	CLDN12	CLDN23	CLDN2
CLDN3	CLDN14		CLDN14
CLDN4	CLDN14-AS1		
CLDN5	CLDN16		
CLDN6	CLDN23		
CLDN7			
CLDN8			

### 3.2 Claudins gene family is associated with functional enrichment and pathways

**FIGURE 4:**
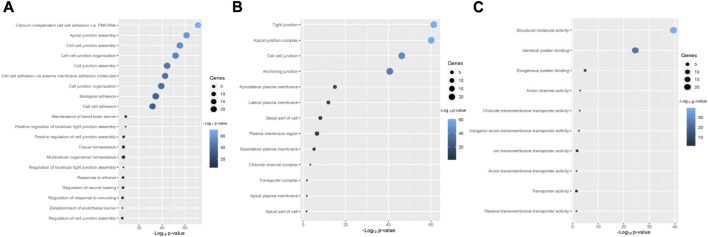
The Claudins family genes are associated with functional enrichment. **(A)**. The top 20 significant biological processes that are associated with Claudins **(B)**. The 13 cellular components that are associated with Claudins. **(C)**. The 10 significant molecular functions associated with Claudins.

**FIGURE 5:**
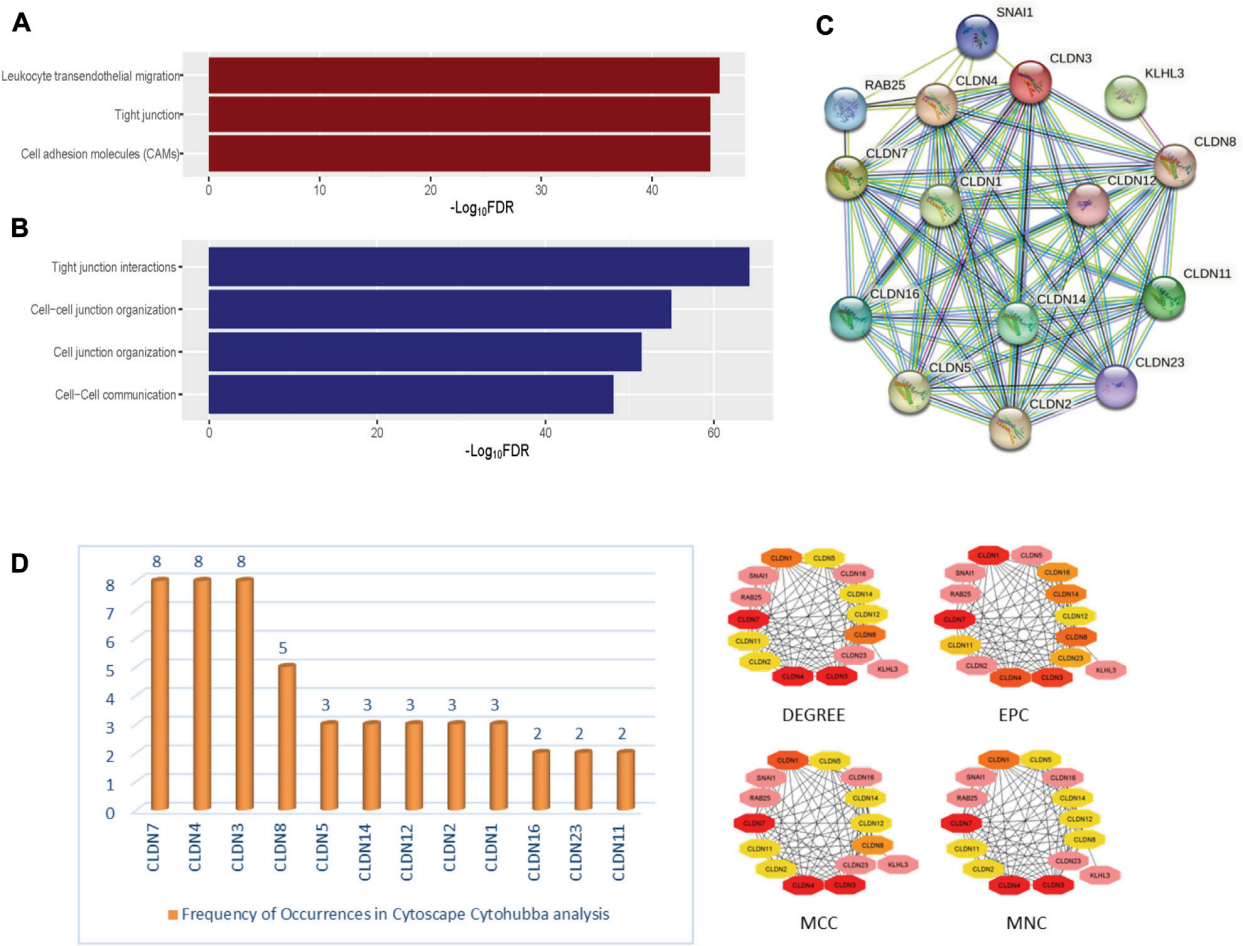
The significantly enriched pathways associated with Claudins. **(A)**. The enriched KEGG pathways **(B)**. the significantly enriched Reactome pathways. **(C)**. The identified protein-protein network of significantly enriched Claudin genes from the Stringdb tool. **(D)**. The identified Claudin network from Cytoscape—Cytohubba analysis.

The enriched gene ontology (GO) terms and pathways were identified by using the GSEA tool (Figure 4; Figure 5). We identified 79 biological processes that are significantly associated with the claudins gene family (Supplementary Table S2). The top 20 biological processes are illustrated in Figure 4A, including alcium independent cell adhesion via plasma membrane cell adhesion molecules, Apical junction assembly, Cell junction assembly, Cell junction organization, Cell junction assembly, cell adhesion via plasma membrane adhesion molecules, Cell junction organization, Biological adhesion, cell adhesion, Maintenance of blood brain barrier, and Positive regulation of bicellular tight junction assembly.

Besides, we found the 13 significant cellular components terms (Tight junction, Apical junction complex, Cell-cell junction, Anchoring junction, Apicolateral plasma membrane, Lateral plasma membrane, Basal part of cell, Plasma membrane region, Basolateral plasma membrane, Chloride channel complex, Transporter complex, Apical plasma membrane, and Apical part of cell) that are associated with claudins (Figure 4B). Moreover, we revealed that the 10 significant molecular functions terms, included Structural molecule activity, Identical protein binding, Exogenous protein binding, Anion channel activity, Chloride transmembrane transporter activity, Inorganic anion transmembrane transporter activity, Ion transmembrane transporter activity, Anion transmembrane transporter activity, Transporter activity, and Passive transmembrane transporter activity are associated with claudins (Figure 4C).

Furthermore, we found that the three KEGG pathways (Leukocyte transendothelial migration, Tight junction, Cell adhesion molecules (CAMs)) and four Reactome pathways (Tight junction interactions, Cell-cell junction organization, Cell junction organization, and Cell-Cell communication) are significantly enriched that are significantly associated with claudins as shown in Figures 5A and 5B. *CLDN1, CLDN16, CLDN4, CLDN3, CLDN7, CLDN23, CLDN19, CLDN14, CLDN15, CLDN17, CLDN20, CLDN11, CLDN18, CLDN22, CLDN5, CLDN10, CLDN8, CLDN6, CLDN2,* and *CLDN9*. Claudins members are associated with the enrichment of three KEGG pathways. Moreover, the other 21 claudins members, included *CLDN5, CLDN16, CLDN4, CLDN3, CLDN7, CLDN23, CLDN19, CLDN14, CLDN15, CLDN17, CLDN20, CLDN11, CLDN18, CLDN22, CLDN12, CLDN10, CLDN8, CLDN6, CLDN2, CLDN1*, and *CLDN9* are associated with enrichment four Reactome pathways.

The pathway enrichment study of the Claudin gene set was done using DAVID Bioinformatics webserver (Sherman et al., 2022) and was again conducted using the BINGO plugin of Cytoscape v. 3.9.1 software, revealing overrepresented biological pathways and Gene Ontology (GO) categories associated with the Claudin gene set is represented in Figure 3B with significant *p*-value as ≤0.05. According to our understanding, Claudins are a group of tight junction proteins that are crucial for maintaining the form and function of epithelial and endothelial barriers. Therefore, it can be very useful to understand the biological roles played by Claudin genes and how likely it is that they participate in specific biological processes. Comparing the list of significant Claudins reveals the important biological pathways that include calcium-independent cell-cell adhesion, cell adhesion, cell adhesion, and biological adhesion.

Occluding junction, tight junction, apical junction complex, apicolateral plasma membrane, cell-cell junction, plasma membrane, membrane part, that is, forming an integral to membrane, and pseudopodium membrane are the cellular components that notably participate in action with the Claudins. The structural molecule activity, identical protein binding, and protein binding capability are molecular functionalities that have been greatly enriched.

### 3.3 Claudins gene family members are involved in the PPI network, gene module and correlated with each other

In-depth relationships and important regulatory components within the claudin family can be discovered by using STRINGdb and Cytoscape CytoHubba parameters for claudin gene network analysis. We may obtain a plethora of information about protein-protein interactions by searching STRINGdb, shown in Figure 5C, with significantly expressed claudin genes as input, which enables the creation of an extensive gene network from GEO datasets, viz. GSE15781 and GSE50760. Utilizing Cytoscape, several topological properties may be exploited. To locate hub genes or highly connected nodes in the claudin network, use CytoHubba. These hub genes are prominent players in the tight junction regulation landscape and may be connected to significant cellular processes. Cytoscape CytoHubba is a powerful plugin that uses network analysis to locate key nodes and hub genes in biological networks. In order to assess the nodes’ topological importance inside the network, it offers a variety of metrics. One of the fundamental features is “degree,” which measures a node’s network connectivity by counting the connections it has with other nodes. Assumed to be an undirected network is the biological network G = (V, E), where V is the set of network nodes and E is the edge set. A network may also be represented by the notation G = [V(G), E(G)], where V(G) is the collection of nodes and E(G) is the collection of edges. When referring to the cardinality (or total number of elements) of a set S, we use the symbol |S|. Local-based methods only take into account a vertex’s immediate vicinity. N(v) represents the collections of a node’s neighbors given a node v. The degree (Deg) technique Deg(v) = |N(v)|. Other important parameters are MNC or Maximum Neighborhood Component (MNC), MCC, or Maximum Clique Centrality and EPC or Edge Percolated Component analysis, where MNC implies (MNC(v) = |V (MC(v))|), where MC(v) is a maximum connected component of the G [N(v)] and G [N(v)] is the subgraph of G, that is, produced when N(v) is applied. We used the MCC method to find prominent nodes in order to improve sensitivity and specificity, where the (Lu et al., 2010) MCC of a node is defined as MCC(v) = CS(v) (|C|1)! for a given node, where S(v) is the collection of maximum cliques that contain v and (|C|-1)! is the product of all positive integers smaller than |C|. MCC(v) is equal to the node’s degree if there is no edge separating its neighbors at node v. Lastly, EPC is used to create reduced networks by assigning a random number between 0 and 1 to every edge and removing edges if their associated random numbers are less than the threshold. For a node v in G, EPC(v) is defined as EPC(v) = 1|V|∑1000k = 1∑t∈Vδkvt. We identified key nodes that are central to the network’s structure and dynamics, shedding light on critical genes or proteins, represented in Figure 5D. Identifying the key players, likely play pivotal roles in biological processes, signaling pathways, and disease mechanisms through Network Biology analysis using the Cytohubba plugin of Cytoscape.

**FIGURE 6:**
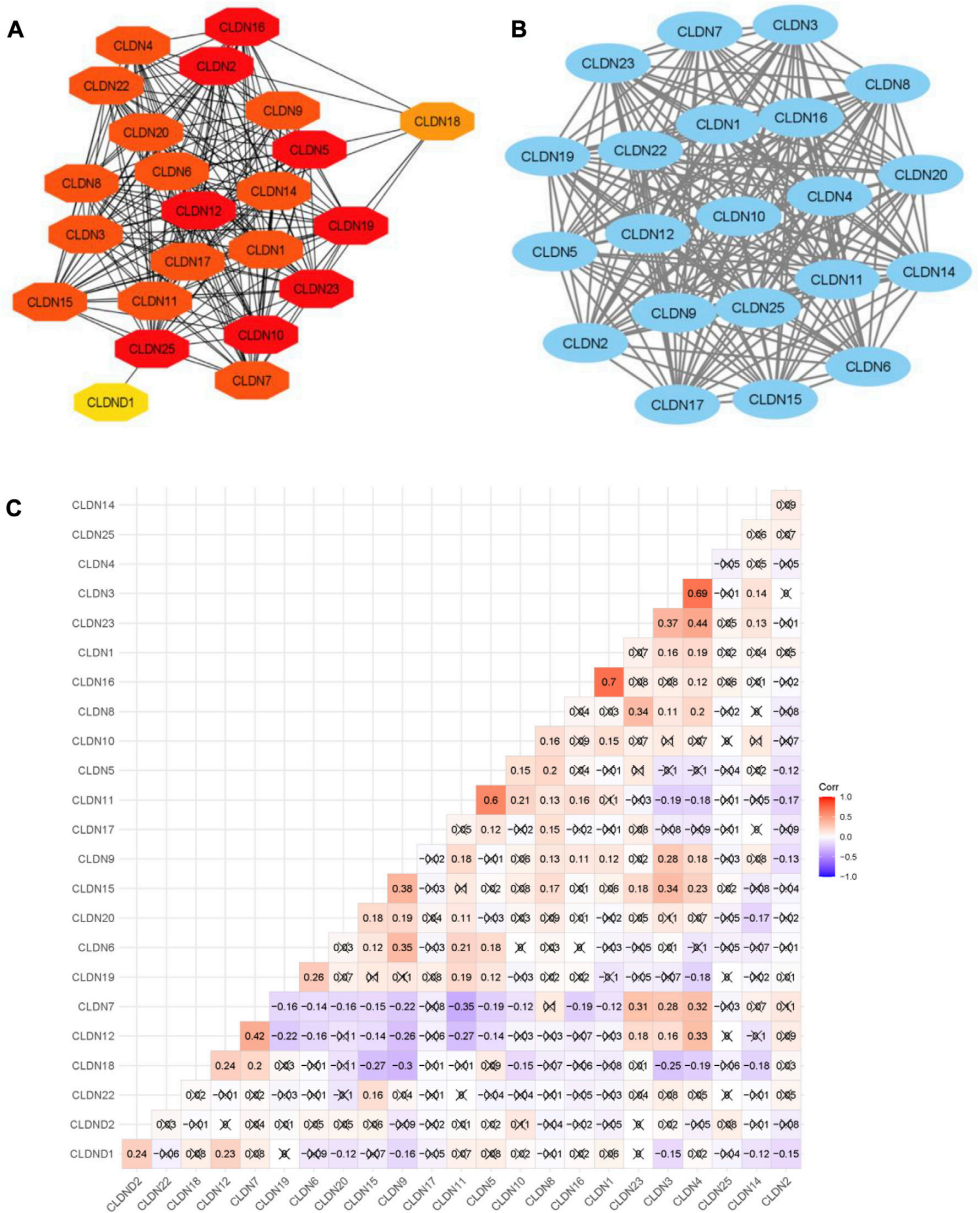
The PPI network of Claudins and correlation of Claudins. **(A)**. The 23 Claudins are involved in the PPI. **(B)**. The claudins member of cluster 1 is associated with PPI network. **(C)**. The correlation of Claudins in the TCGA COAD data (Pearson correlation test). × indicated the non-significant value.

The ability of certain aberrantly produced proteins to promote tumors under malignant settings directly affects their capacity to interact with a protein-binding regulatory partner, making the targeting of protein-protein interactions significantly relevant to cancer (Garner and Janda, 2011). We used the STRING tool to look at the PPI interaction of all claudins from both the Pipeline 1 and Pipeline 2 candidate claudin gene family. We discovered that the PPI involves the following genes: CLDN10, CLDN2, CLDN16, CLDN23, CLDN12, CLDN19, CLDN25, CLDN5, CLDN8, CLDN4, CLDN15, CLDN1, CLDN11, CLDN9, CLDN17, CLDN3, CLDN22, CLDN20, CLDN14, CLDN6, CLDN7, CLDN18, and CLDND1. The 21 additional nodes are connected to the eight claudins (CLDN10, CLDN2, CLDN16, CLDN23, CLDN12, CLDN19, CLDN25, and CLDN5). Additionally, the 13 other claudins (CLDN8, CLDN4, CLDN15, CLDN1, CLDN11, CLDN9, and CLDN17) have interacted with the other 20 claudins of the PPI. While CLDN1 only interacted with CLDN25, CLDN18 interacted with the other 7 members of the claudin family (Figure 6A).

**FIGURE 7:**
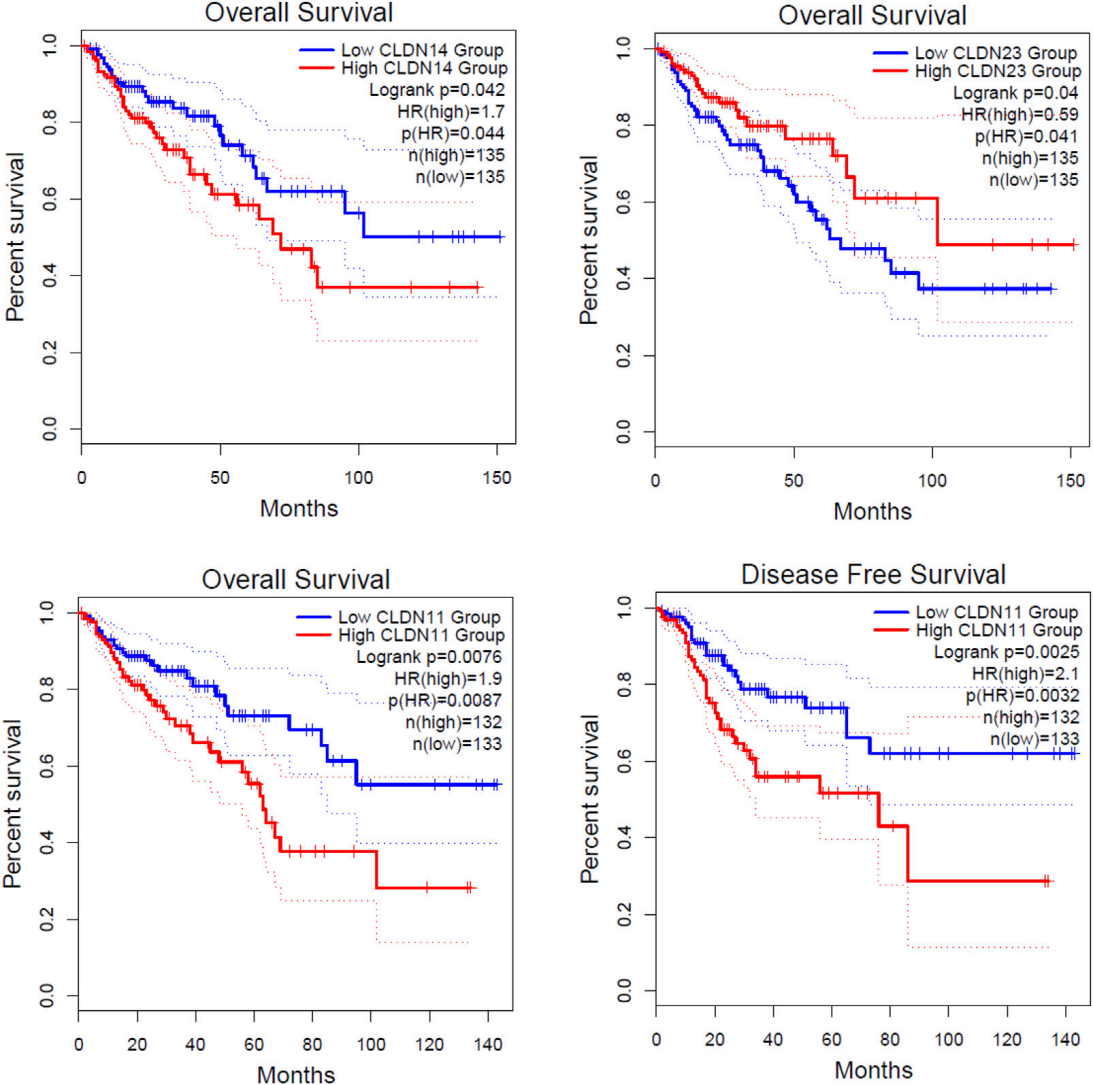
Identification of prognostic DEGs in the COAD. The higher expression group of CLDN11 and *CLDN14* are significantly correlated with shorter survival time in colon adenocarcinoma. The lower expression group of CLDN23 is significantly correlated with a shorter survival time in colon adenocarcinoma.

In addition, we identified the core module from the original PPI network by using the Cytoscape plug-in MCODE. Interestingly, we found a single gene module with a high Cluster Score (MCODE identified score is 21). In this key gene module or cluster, 21 claudins members (*CLDN7, CLDN5, CLDN6, CLDN14, CLDN20, CLDN25, CLDN19, CLDN22, CLDN3, CLDN12, CLDN23, CLDN17, CLDN9, CLDN16, CLDN2, CLDN11, CLDN1, CLDN15, CLDN4, CLDN10,* and *CLDN8*) are associated with PPI network (Figure 7B). STRING-based analysis revealed that this module is associated with functional enrichment (Table 3).

**TABLE 3:** Functional enrichment analysis of the entire working pipeline.

Functional terms	Term id	Term description	Observed gene count	Background gene count	False discovery rate	Matching proteins in your network (labels)
KEGG pathways	hsa04670	Leukocyte transendothelial migration	20	109	3.53E-41	CLDN11,CLDN16,CLDN17,CLDN1,CLDN19,CLDN10,CLDN22,CLDN7,CLDN20,CLDN3,CLDN6,CLDN14,CLDN8,CLDN15,CLDN5,CLDN25,CLDN9,CLDN4,CLDN23,CLDN2
	hsa04514	Cell adhesion molecules	20	137	1.20E-39	CLDN11,CLDN16,CLDN17,CLDN1,CLDN19,CLDN10,CLDN22,CLDN7,CLDN20,CLDN3,CLDN6,CLDN14,CLDN8,CLDN15,CLDN5,CLDN25,CLDN9,CLDN4,CLDN23,CLDN2
	hsa04530	Tight junction	20	156	9.04E-39	CLDN11,CLDN16,CLDN17,CLDN1,CLDN19,CLDN10,CLDN22,CLDN7,CLDN20,CLDN3,CLDN6,CLDN14,CLDN8,CLDN15,CLDN5,CLDN25,CLDN9,CLDN4,CLDN23,CLDN2
	hsa05160	Hepatitis C	20	156	9.04E-39	CLDN11,CLDN16,CLDN17,CLDN1,CLDN19,CLDN10,CLDN22,CLDN7,CLDN20,CLDN3,CLDN6,CLDN14,CLDN8,CLDN15,CLDN5,CLDN25,CLDN9,CLDN4,CLDN23,CLDN2
	hsa05130	Pathogenic *Escherichia coli* infection	20	187	1.65E-37	CLDN11,CLDN16,CLDN17,CLDN1,CLDN19,CLDN10,CLDN22,CLDN7,CLDN20,CLDN3,CLDN6,CLDN14,CLDN8,CLDN15,CLDN5,CLDN25,CLDN9,CLDN4,CLDN23,CLDN2
Reactome pathways	HSA-420029	Tight junction interactions	20	30	7.63E-50	CLDN11,CLDN16,CLDN17,CLDN12,CLDN1,CLDN19,CLDN10,CLDN22,CLDN7,CLDN20,CLDN3,CLDN6,CLDN14,CLDN8,CLDN15,CLDN5,CLDN9,CLDN4,CLDN23,CLDN2
GENE ONTOLOGY Biological process	#term ID	term description	observed gene count	background gene count	false discovery rate	matching proteins in your network (labels)
	GO:0016338	Calcium-independent cell-cell adhesion via plasma membrane cell-adhesion molecules	20	21	2.58E-51	CLDN11,CLDN16,CLDN17,CLDN12,CLDN1,CLDN19,CLDN10,CLDN22,CLDN7,CLDN20,CLDN3,CLDN6,CLDN14,CLDN8,CLDN15,CLDN5,CLDN9,CLDN4,CLDN23,CLDN2
	GO:0070830	Bicellular tight junction assembly	20	58	9.47E-45	CLDN11,CLDN16,CLDN17,CLDN1,CLDN19,CLDN10,CLDN22,CLDN7,CLDN20,CLDN3,CLDN6,CLDN14,CLDN8,CLDN15,CLDN5,CLDN25,CLDN9,CLDN4,CLDN23,CLDN2
	GO:0007155	Cell adhesion	21	925	1.99E-25	CLDN11,CLDN16,CLDN17,CLDN12,CLDN1,CLDN19,CLDN10,CLDN22,CLDN7,CLDN20,CLDN3,CLDN6,CLDN14,CLDN8,CLDN15,CLDN5,CLDN25,CLDN9,CLDN4,CLDN23,CLDN2
	GO:0016337	cell-cell adhesion	12	291	3.52E-19	CLDN11,CLDN5,CLDN4,CLDN3,CLDN14,CLDN8,CLDN23,CLDN7,CLDN12,CLDN16,CLDN2,CLDN1
	GO:0022610	biological adhesion	12	712	1.75E-14	CLDN11,CLDN5,CLDN4,CLDN3,CLDN14,CLDN8,CLDN23,CLDN7,CLDN12,CLDN16,CLDN2,CLDN1
	GO:0035633	Maintenance of blood-brain barrier	4	34	5.02E-05	CLDN12,CLDN1,CLDN3,CLDN5
	GO:1903348	Positive regulation of bicellular tight junction assembly	3	6	6.01E-05	CLDN1,CLDN3,CLDN5
	GO:1901890	Positive regulation of cell junction assembly	4	102	0.0028	CLDN1,CLDN19,CLDN3,CLDN5
	GO:0061041	Regulation of wound healing	4	140	0.0089	CLDN1,CLDN19,CLDN3,CLDN4
	GO:0048871	Multicellular organismal homeostasis	5	352	0.0175	CLDN12,CLDN1,CLDN3,CLDN5,CLDN4
	GO:0090303	Positive regulation of wound healing	3	59	0.0198	CLDN1,CLDN3,CLDN4
	GO:1905050	Positive regulation of metallopeptidase activity	2	9	0.0289	CLDN3,CLDN4
GENE ONTOLOGY Cellular components	GO:0005923	Bicellular tight junction	21	122	4.95E-43	CLDN11,CLDN16,CLDN17,CLDN12,CLDN1,CLDN19,CLDN10,CLDN22,CLDN7,CLDN20,CLDN3,CLDN6,CLDN14,CLDN8,CLDN15,CLDN5,CLDN25,CLDN9,CLDN4,CLDN23,CLDN2
	GO:0016327	Apicolateral plasma membrane	6	19	2.94E-11	CLDN7,CLDN3,CLDN6,CLDN8,CLDN5,CLDN4
	GO:0016021	Integral component of membrane	21	5181	1.67E-10	CLDN11,CLDN16,CLDN17,CLDN12,CLDN1,CLDN19,CLDN10,CLDN22,CLDN7,CLDN20,CLDN3,CLDN6,CLDN14,CLDN8,CLDN15,CLDN5,CLDN25,CLDN9,CLDN4,CLDN23,CLDN2
	GO:0005886	Plasma membrane	21	5314	2.52E-10	CLDN11,CLDN16,CLDN17,CLDN12,CLDN1,CLDN19,CLDN10,CLDN22,CLDN7,CLDN20,CLDN3,CLDN6,CLDN14,CLDN8,CLDN15,CLDN5,CLDN25,CLDN9,CLDN4,CLDN23,CLDN2
	GO:0016328	Lateral plasma membrane	6	61	9.40E-09	CLDN12,CLDN1,CLDN7,CLDN3,CLDN15,CLDN4
	GO:0016323	Basolateral plasma membrane	5	237	0.00058	CLDN1,CLDN19,CLDN7,CLDN8,CLDN4
	GO:0070160	Occluding junction	12	74	2.55E-25	CLDN11,CLDN5,CLDN4,CLDN3,CLDN14,CLDN8,CLDN23,CLDN7,CLDN12,CLDN16,CLDN2,CLDN1
	GO:0043296	Apical junction complex	12	85	1.02E-24	CLDN11,CLDN5,CLDN4,CLDN3,CLDN14,CLDN8,CLDN23,CLDN7,CLDN12,CLDN16,CLDN2,CLDN1
	GO:0005911	Cell-cell junction	12	192	1.68E-20	CLDN11,CLDN5,CLDN4,CLDN3,CLDN14,CLDN8,CLDN23,CLDN7,CLDN12,CLDN16,CLDN2,CLDN1
	GO:0030054	Cell junction	12	522	2.70E-15	CLDN11,CLDN5,CLDN4,CLDN3,CLDN14,CLDN8,CLDN23,CLDN7,CLDN12,CLDN16,CLDN2,CLDN1
	GO:0044459	Plasma membrane part	13	1999	6.09E-10	CLDN2,CLDN1,CLDN11,CLDN5,CLDN4,CLDN3,RAB25,CLDN14,CLDN8,CLDN23,CLDN7,CLDN12,CLDN16
	GO:0044425	Membrane part	13	6114	5.42E-04	CLDN2,CLDN1,CLDN11,CLDN5,CLDN4,CLDN3,RAB25,CLDN14,CLDN8,CLDN23,CLDN7,CLDN12,CLDN16
	GO:0016021	Integral to membrane	12	5266	7.70E-04	CLDN11,CLDN5,CLDN4,CLDN3,CLDN14,CLDN8,CLDN23,CLDN7,CLDN12,CLDN16,CLDN2,CLDN1
	GO:0031224	Intrinsic to membrane	12	5375	8.72E-04	CLDN11,CLDN5,CLDN4,CLDN3,CLDN14,CLDN8,CLDN23,CLDN7,CLDN12,CLDN16,CLDN2,CLDN1
	GO:0016020	Membrane	13	7253	2.82E-03	CLDN2,CLDN1,CLDN11,CLDN5,CLDN4,CLDN3,RAB25,CLDN14,CLDN8,CLDN23,CLDN7,CLDN12,CLDN16
	GO:0098590	Plasma membrane region	8	1219	0.0026	CLDN1,CLDN19,CLDN7,CLDN3,CLDN6,CLDN8,CLDN5,CLDN4
GENE ONTOLOGY Molecular functions	GO:0005198	Structural molecule activity	20	635	1.52E-25	CLDN11,CLDN16,CLDN17,CLDN1,CLDN19,CLDN10,CLDN22,CLDN7,CLDN20,CLDN3,CLDN6,CLDN14,CLDN8,CLDN15,CLDN5,CLDN25,CLDN9,CLDN4,CLDN23,CLDN2
	GO:0042802	Identical protein binding	20	1896	1.81E-16	CLDN11,CLDN16,CLDN17,CLDN12,CLDN1,CLDN19,CLDN10,CLDN22,CLDN7,CLDN20,CLDN3,CLDN6,CLDN14,CLDN8,CLDN15,CLDN5,CLDN9,CLDN4,CLDN23,CLDN2
	GO:0005515	Protein binding	15	8123	7.99E-04	KLHL3,CLDN2,CLDN1,CLDN11,CLDN5,CLDN4,CLDN3,RAB25,CLDN14,CLDN8,CLDN23,CLDN7,CLDN12,SNAI1,CLDN16

After getting the interaction of claudins, we thought that the claudins are correlated with each other (Absolute value of Pearson correlation, R > 0.2, *p* ≤ 0.05). Our analysis revealed that the expression level of claudins is correlated with other members (Figure 6C). For example, the expression level of *CLDN1* is positively correlated with *CLDN2* and *CLDN12*. Similarly, *CLDN*4 is positively correlated with *CLDN1, CLDN3, CLDN12, CLDN7,* and *CLDN15*. In contrast, the expression level of *CLDN18* is negatively correlated with *CLDN15, CLDN9,* and *CLDN3*.

### 3.4 Claudins are associated with poor survival prognosis in the COAD

We investigated the survival significance of all differentially expressed significant claudins (*CLDN2, CLDN1, CLDN14, CLDN16, CLDN18, CLDN9, CLDN12, CLDN6, CLDN8, CLDN23, CLDN5, CLDN11, CLDN7,* and *CLDN15*) in TCGA COAD data. The patients’ characteristics, such as the severity of the cancer grade, age, survival status, and other clinical pathological features are presented in the Supplementary Table S3. Our analysis revealed that the higher expression group of *CLDN14* and *CLDN11* are significantly correlated with the survival prognosis of colon cancer patients (Figure 7) and the low expression group of *CLDN23* is significantly correlated with shorter survival time of colon cancer patients (Figure 7). The median expression value of CLDN14 in the high expression group of TCGA data is 5.12 (Log2 transformed) and the medial value of the low expression group is 2.67 (Log2 transformed). Similarly, the median value of CLDN11 in the high expression group is 5.25 (Log2 transformed) and the medial value of the low expression group is 2.87 (Log2 transformed). The two genes have obvious median differences between the groups of patients. On the other hand, the median expression value of CLDN23 in the high expression group is 8.91 (Log2 transformed) and the medial value of the low expression group is 7.31 (Log2 transformed). These claudins are associated with the survival prognosis of cancer patients. For example, *CLDN11* and *CLDN14* are correlated with prognostic values in human breast carcinoma (Jia et al., 2019). The risk score models identify that the *CLDN23* is correlated to the disease prognosis in colon cancer patients (Yang et al., 2019). The hypermethylated *CLDN11* is related to the metastasis of CRC and also related to the prognosis of poor survival of CRC (Li et al., 2017a). It indicates that the expression levels of *CLDN14, CLDN11,* and *CLDN23* are key regulators in the COAD.

### 3.5 Claudins are associated with immune infiltrations in the COAD

**FIGURE 8:**
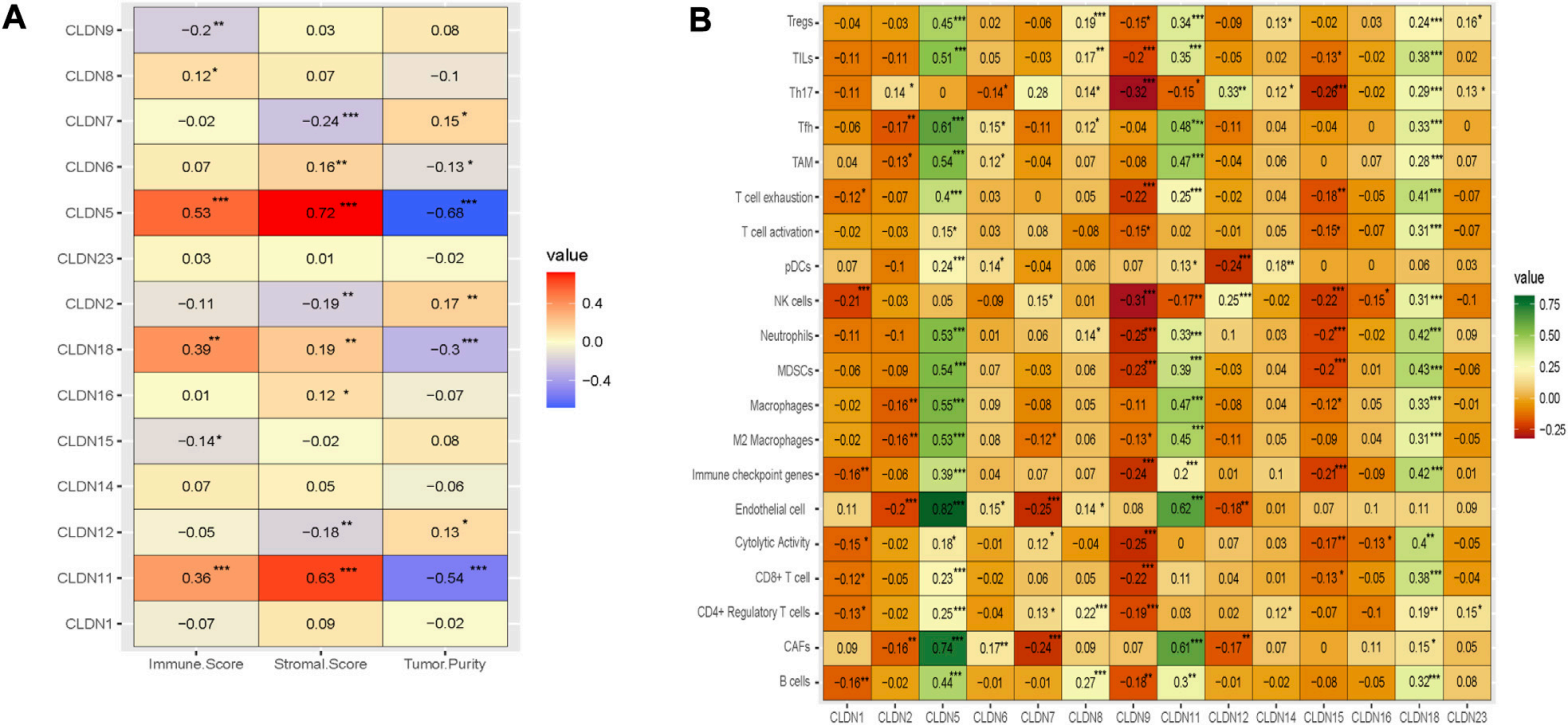
The association of claudins with the regulation of tumor microenvironment in the COAD. **(A)**. Claudins are associated with immune scores, stromal scores, and tumor purity. **(B)**. The various claudins genes are correlated with immune infiltrations in the COAD.

We investigated the regulation of the tumor microenvironment by Claudins genes. We found that the immune score is positively correlated with *CLDN5, CLDN11,* and *CLDN18* and negatively correlated with *CLDN9* (Absolute value of Spearman Correlation, R > 0.20 and *p* < 0.001) (Figure 8A). Besides, the stromal score is positively correlated with *CLDN5* and *CLDN11* and negatively correlated with *CLDN7* (Absolute value of Spearman Correlation is 0.20 and *p* < 0.001) (Figure 8A). Tumor purity, another substantial parameter for the tumor microenvironment, is negatively correlated with *CLDN5, CLDN11,* and *CLDN18* (Absolute value of Spearman Correlation is 0.20 and *p* < 0.001) (Figure 8A). This result indicates that the expression of *CLDN5, CLDN11,* and *CLDN18* in the COAD may be regulating the tumor microenvironment.

Moreover, we analyze the relationship between the immune infiltrations and the expression level of differentially expressed claudin gene members (R > 0.20 and *p* < 0.001) (Figure 8B). The expression level of *CLDN5* is positively correlated with the various immune signatures, including B cells, CAFs, CD4 Regulatory T cells, CD8 T cells, Endothelial cells, Immune checkpoint genes, M2 Macrophages, Macrophages, MDSCs, Neutrophils, pDC, T cell exhaustion, TAM, Tfh, TILs, and Tregs (R > 0.20 and *p* < 0.001) (Figure 8B). Also, the expression level of *CLDN11* is positively correlated with the activity of B cells, CAFs, CD8 T cells, Endothelial cells, Immune checkpoint genes, M2 Macrophages, Macrophages, MDSCs, Neutrophils, T cell exhaustion, TAM, Tfh, TILs, and Tregs (R > 0.20 and *p* < 0.001) (Figure 8B). In addition, the expression level of *CLDN18* is positively correlated with the various immune signatures, including B cells, CD8 T cells, Immune checkpoint genes, M2 Macrophages, Macrophages, MDSCs, Neutrophils, T cell exhaustion, TAM, Tfh, Th17, TILs, and Tregs (R > 0.20 and *p* < 0.001) (Figure 8B). On the other hand, we found a significant negative correlation of *CLDN9* with some immune signatures, including, CD8 T cell, cytolytic activity, Immune checkpoint genes, MDSCs, Neutrophils, NK cells, T cell exhaustion, Th17, and TILs (R > −0.20 and *p* < 0.001) (Figure 8B). Similarly, the expression level of *CLDN15* is negatively correlated with Immune checkpoint genes, MDSCs, Neutrophils, NK cells, and Th17 (R > −0.20 and *p* < 0.001) (Figure 8B). Altogether, it indicated that the expression level of *CLDN5, CLDN9, CLDN11, CLDN15,* and *CLDN18* are associated with immune infiltrations in the COAD.

### 3.6 Claudins are correlated with cancerous biological phenotypes in COAD

**FIGURE 9:**
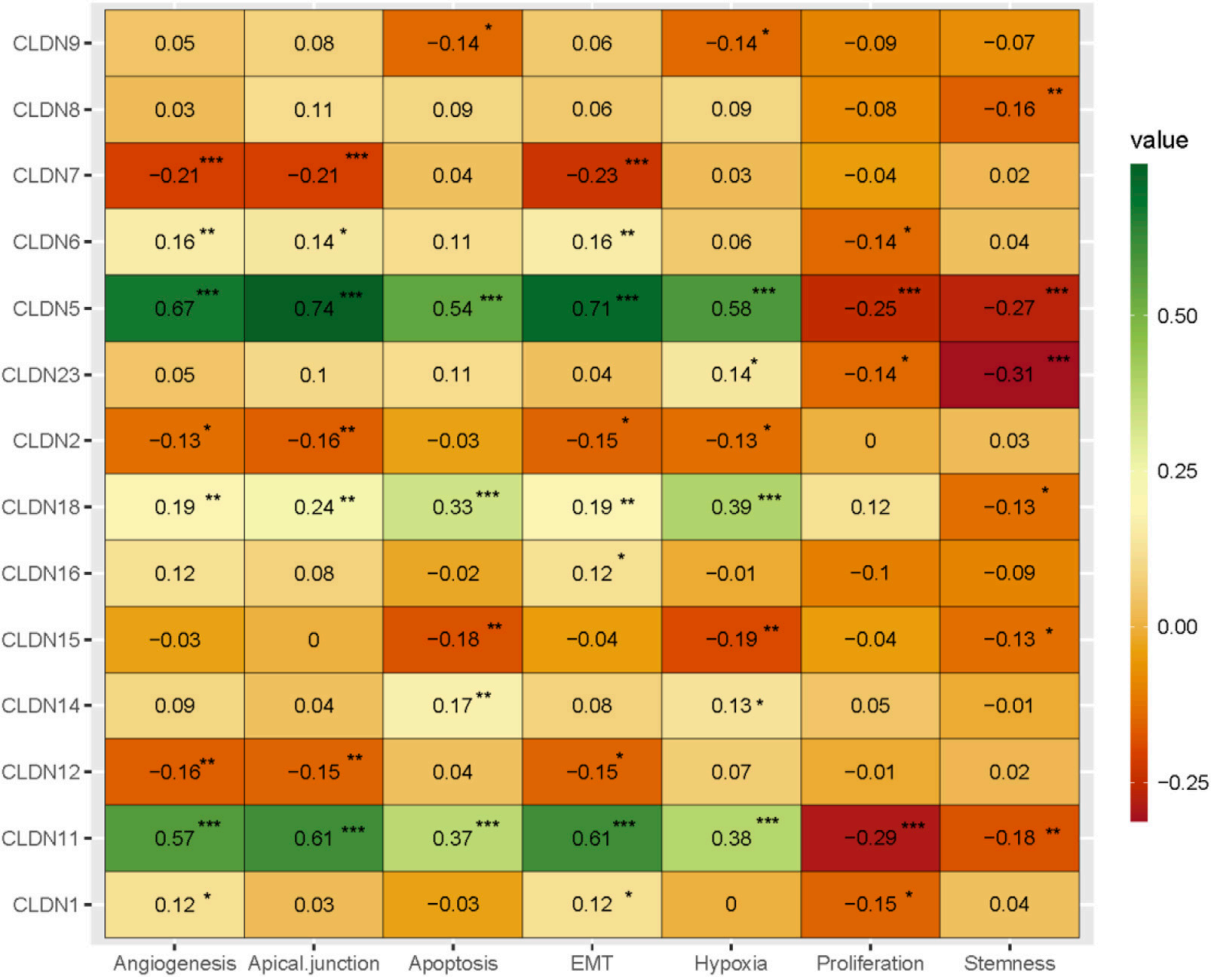
The association of claudins with the regulation of angiogenesis, apical junction, apoptosis, epithelial-mesenchymal transition (EMT), hypoxia, proliferation, and stemness in the COAD and GSEA datasets.

Angiogenesis, apical junction, apoptosis, epithelial-mesenchymal transition (EMT), hypoxia, Proliferation, and stemness are major cancerous biological processes in cancers. We investigated the association of these biological processes with the expression level of claudin genes (Absolute value of Spearman Correlation is 0.20 and *p* < 0.001). We revealed that the expression of *CLDN5* and *CLDN11* are positively correlated with angiogenesis, apical junction, apoptosis, epithelial-mesenchymal transition (EMT), and hypoxia, and negatively (Figure 9) correlated with proliferation and stemness, indicating that it may be acted as a supportive and protective factor in COAD. The expression level of *CLDN18* is positively correlated with apoptosis and hypoxia (Figure 9). It indicates that the expression of claudins regulating the aggressive phenotypes of cancers in the COAD.

### 3.7 Claudins gene family members regulating the cancer-associated pathways

**FIGURE 10:**
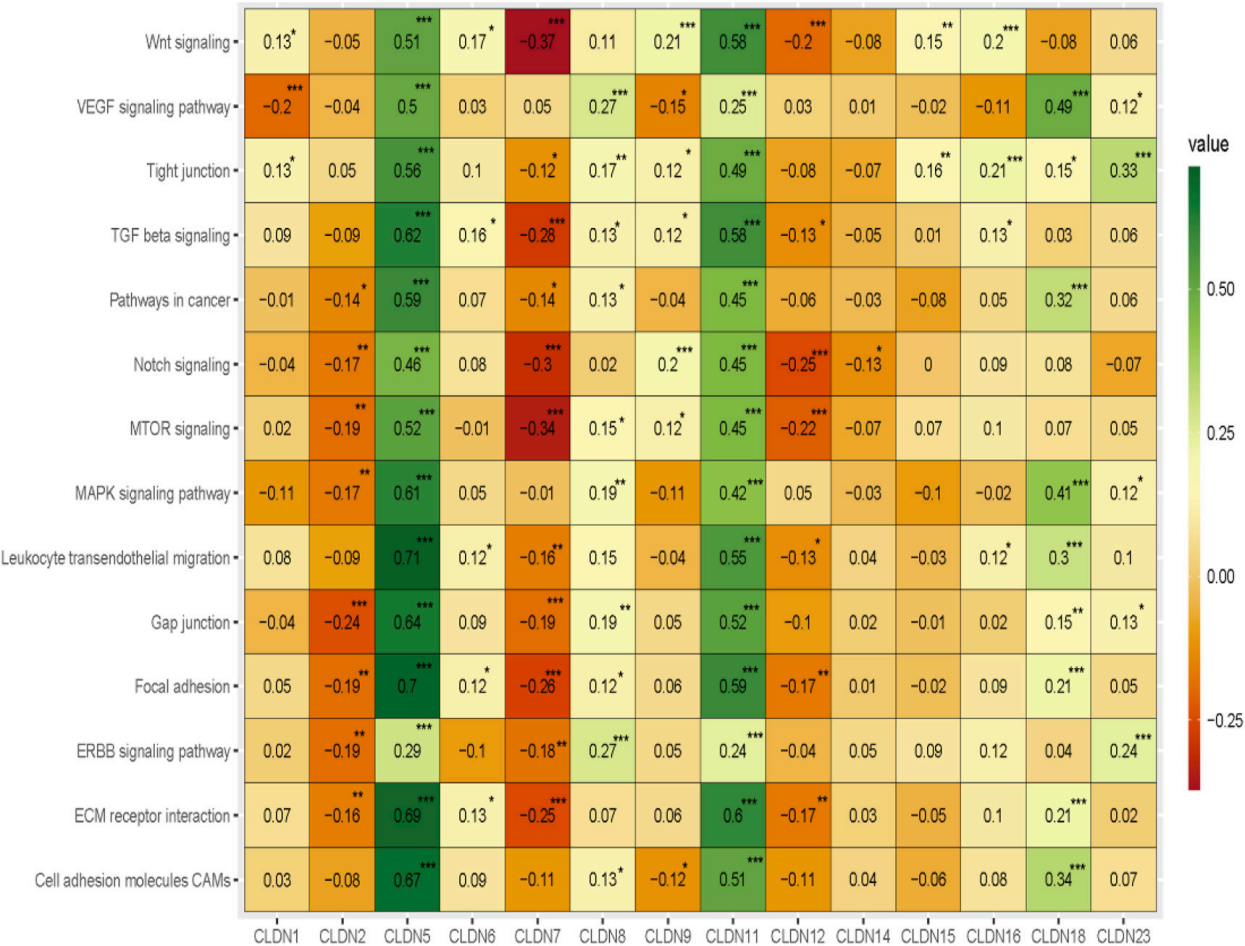
The association of claudins with the regulation of cancer-associated pathways in the COAD and GSEA datasets.

Since our analysis identified the association of claudins with immune infiltrations and cancerous biological processes, we investigated the correlation of claudins with cancerous pathways activity (Absolute value of Spearman Correlation is 0.20 and *p* < 0.001). Interestingly, the expression levels of *CLDN5* and *CLDN11* are positively associated with the activity of cell adhesion molecules CAMs, ECM receptor interaction, ERBB signaling pathway, focal adhesion, gap junction, leukocyte transendothelial migration, MAPK signaling pathway, MTOR signaling, Notch signaling, pathways in cancer, TGF beta signaling, tight junction, VEGF signaling pathway, and Wnt signaling (Figure 10).

Besides, the expression levels of *CLDN2, CLDN7,* and *CLDN12* are negatively correlated with some of the cancer-associated pathways (Figure 10). *CLDN2* is negatively correlated with gap junction and *CLDN12* is negatively correlated with MTOR signaling and Notch signaling pathways (Figure 10). Besides, the expression level of *CLDN7* is negatively associated with the activity of ECM receptor interaction, focal adhesion, MTOR signaling, Notch signaling, TGF beta signaling, and Wnt signaling pathways (Figure 10). Altogether, it indicates that the expression of *CLDN5, CLDN11, CLDN2, CLDN7,* and *CLDN12* is associated with regulating the key cancer-associated pathways in the COAD and GSEA datasets.

### 3.8 Claudins gene family members are mutated in the COAD

**FIGURE 11:**
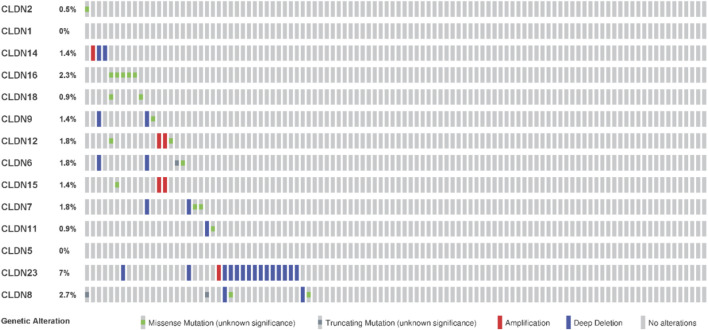
Mutation of differentially expressed claudin genes in the COAD. The mutation was evaluated by using the TCGA COAD dataset in cBioPortal.

We investigated the genetic alterations of all differentially expressed claudins in the COAD GSEA datasets. We found that the 14 Queried DEGs genes are altered in 38 (17%) of queried patients out of the 220 patients with mutation and CNA data. The top mutated claudins in the COAD is CLDN23 (7%) (Figure 11). The other mutated claudins are *CLDN2* (0.5%), *CLDN14* (1.4%), *CLDN16* (2.3%), *CLDN18* (0.9%), *CLDN9* (1.4), *CLDN12* (1.8%), *CLDN8* (2.7%), *CLDN11* (0.9%), *CLDN7* (1.8%) and *CLDN15* (1.4) (Figure 11).

### 3.9 Claudins exhibited the diagnostic efficacy in colon cancers

**FIGURE 12:**
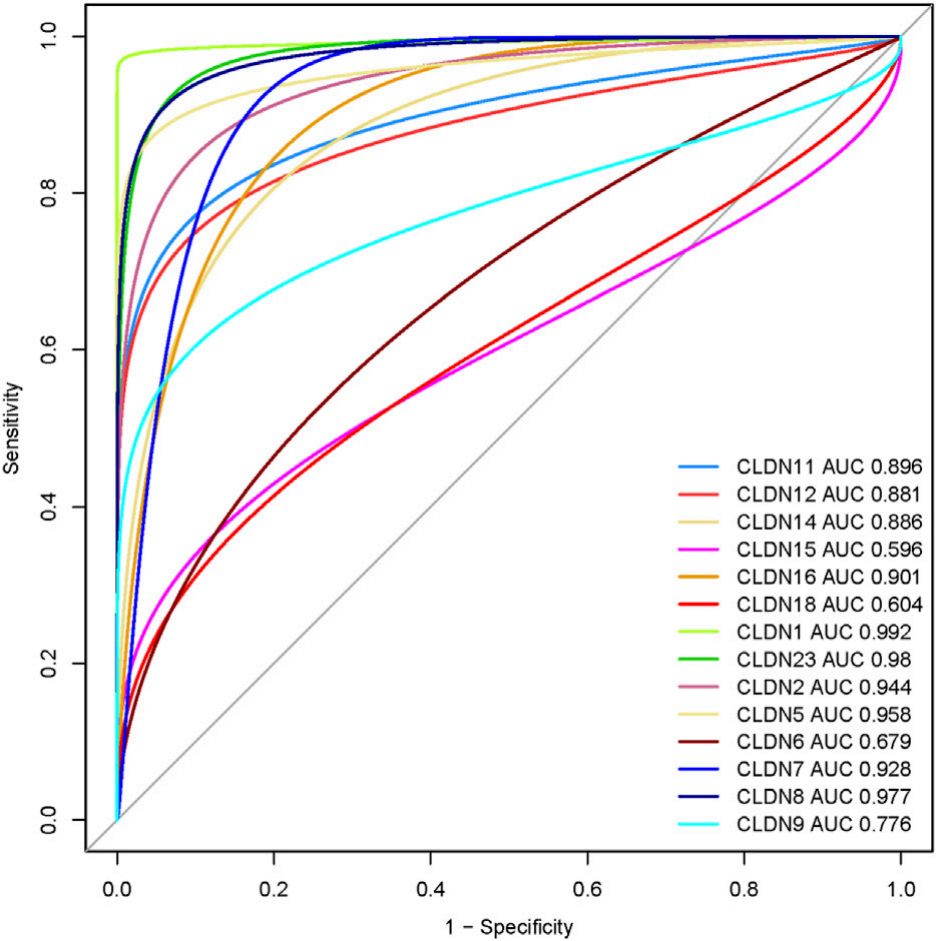
Evaluation of diagnostic efficacy of differentially expressed claudin genes in the COAD. The receiver operating characteristic (ROC) curve of claudin genes in colon cancer (TCGA COAD dataset).

We speculate that these differentially expressed claudins genes (*CLDN2, CLDN1, CLDN14, CLDN16, CLDN18, CLDN9, CLDN12, CLDN6, CLDN8, CLDN23, CLDN5, CLDN11, CLDN7,* and *CLDN15*) have diagnostic value in colon cancer. We used the TCGA COAD and GSEA datasets to validate our hypothesis, and the results showed that the ROC curve of the expression levels of these genes showed excellent diagnostic value for colon cancer cases (AUC>0.5) (Figure 12). We found that the AUC value of *CLDN2, CLDN1, CLDN14, CLDN16, CLDN12, CLDN8, CLDN23, CLDN5, CLDN11,* and *CLDN7* is above 0.80, indicating that these claudins have strong diagnostic value for colon cancer patients. Altogether, it can be hypothesized that the claudins are associated with the diagnostic efficacy in both the GSEA datasets and the TCGA COAD database.

## 4 Discussion

Identifying aberrantly expressed claudins and their carcinogenic effects in colon cancer is important because they are linked to neoplastic transition, tumor biology, tumor growth, cancer cell motility and dissemination, and cancer metastasis (Tabariès and Siegel, 2016; Li, 2021b). To close this information gap, we found 14 caludins in COAD that were unregulated (Table 1; Figure 3). Claudins have been repeatedly linked to the etiology of colon and other cancers, according to certain prior investigations. For instance, it has been shown that a new marker for colorectal cancer is the expression of CLDN1 (Nakagawa et al., 2011). The upregulated level of *CLDN*2 increased the tumorigenicity of colon cancer cells (Dhawan et al., 2011). A higher level of Claudin-2 promotes colorectal cancer liver metastasis and it acts as a crucial biomarker for the replacement type growth pattern (Tabariès et al., 2021). Li et al. (2016) identified that the *CLDN14* is a potentially direct target for EZH2-mediated H3K27ME3 in human hepatocellular carcinoma. The expression level of *CLDN18* is correlated with poor survival in patients with CRC and is associated with the phenotype of gastric cancer (Matsuda et al., 2010). In addition to the upregulated claudins, various claudins are downregulated in colon cancer (Sara Cherradi et al.). demonstrated that *CLDN5, CLDN7,* and *CLDN23* were downregulated in CRC samples (Cherradi et al., 2019). Altogether, it indicates that deregulated claudins are associated with colon cancer pathogenesis.

With 1.4 million new cases each year, colorectal cancer (CRC) is presently the second most frequent malignancy in women and the third most common in men worldwide. By 2030, it is anticipated that there will be 1.1 million fatalities and more than 2.2 million new cases of colorectal cancer (CRC). In many low- and middle-income countries, CRC incidence and mortality rates continue to rise quickly; in more developed nations, although rates remain among the highest in the world, stabilizing or declining trends are more common. Moreover, we found that Claudins are associated with the enrichment of gene ontology and signaling pathways (Figure 4; Figure 5). GO and pathway analysis revealed that the significant terms are mainly involved with immune regulation and cellular communication (Figure 4; Figure 5). Ryan C Winger et al. revealed that claudins are associated with the leukocyte transendothelial migration in a human model of the blood-brain barrier (Winger et al., 2014). The claudins are the backbone of tight junctions that control the signaling pathways in Inflammation, cell proliferation, transformation, and metastasis (Bhat et al., 2019). Claudins have been identified as crucial cell adhesion molecules working at tight junctions (Mori, 2011). Altogether, it indicates that the expression of claudins regulates the various biological signaling pathways in colon cancer. In addition, we identified hub claudins that interacted with other members (Figure 6A). It was demonstrated that the aberrant expression of *CLDN2, CLDN4, CLDN5, CLDN7,* and *CLDN23* are associated with the clinical value in colorectal tumors (Cherradi et al., 2019). These findings indicated that the claudins are involved in the PPI network-mediated cellular signaling.

Then, we found that the expression of three deregulated claudins (*CLDN11, CLDN14, and CLDN23*) is significantly correlated with the shorter survival time of colon cancer patients as well as in the metadata of GSEA datasets (Figure 7). The higher expression level of *CLDN11* is correlated with decreased infiltration levels of CD8^+^ cells and NK cells and increased levels of immunosuppressive components, including CAFs, TAM, MDSCs, etc (Figure 8B). It indicated that CLDN11 was unfavorable for the anti-tumor immune process. The immunosuppressive genes retards the antitumor immune process (Xu et al., 2021). CLDN11 is correlated with lowering tumor purity (Figure 8A). Low tumor purity is associated with poor prognosis in colon cancer (Mao et al., 2018). Genes highly expressed in the microenvironment are expected to have negative associations with tumor purity, while the opposite is expected for genes highly expressed in the tumor cells (Li et al., 2017b). The expression of CLDN11 is may be higher in the tumor microenvironment which ultimately associated with immunosuppression and poor prognosis in the COAD.

Since the level of immune infiltration is a substantial predictor of a patient’s survival in cancer (Ohtani, 2007), we analyzed the correlation of deregulated claudins with the immune infiltration levels in COAD. The previous reports demonstrated that claudins are associated with immune infiltrations in human cancer (Gao et al., 2021). Therefore, these consistent findings indicated that claudins are crucial predictors of a patient’s survival prognosis and immune infiltrations in COAD. Angiogenesis, apical junction, apoptosis, epithelial-mesenchymal transition (EMT), hypoxia, proliferation, and stemness are major cancerous biological processes that influence the pathogenesis of disease. We investigated the association of these biological processes with the expression level of claudins in COAD and GSEA datasets (Figure 9). The expression of *CLDN5* is associated with breast cancer cell motility, indicating the role of *CLDN5* in the metastasis of human breast cancer (Escudero-Esparza et al., 2012). The regulatory axis Snail-claudin-11 influences the formation of circulating tumor cell clusters, which are associated with tumor progression (Li et al., 2019). The downregulation of Claudin-7 induces metastasis and invasion in colorectal cancer via the promotion of EMT (Wang et al., 2019). For identifying the implications for disease behavior and prevention, it is necessary to identify the correlation of claudins with cancer-associated pathways. We found that several deregulated claudins are associated with the activity of cancer-associated pathways (Figure 10). It was indicated that the claudins are related to the cancerous-associated pathways in cancers. For example, the hypermethylation of the *CLDN11* promoter region in CRC cells is committed to the metastasis of cells (Li et al., 2017a). Altogether, it indicates that the expression of claudins is associated with the regulation of cancerous phenotypes and pathways in COAD. Furthermore, we found that the claudins are mutated (Figure 11) and it has strong diagnostic value for colon cancer patients (Figure 12). Recently, it was stated that the *CLDN15* is a diagnostic marker for malignant pleural mesothelioma (Watanabe et al., 2021). *CLDN1*, a gene with diagnostic value, acted as the novel marker in CRC 35. A near-infrared tagged peptide, claudin-1 was recently utilized to detect endoscopically pre-malignant colonic adenomas. Recent research has also demonstrated that the traditional Chinese medication Antrodia camphorta (AC) inhibits the epithelial-mesenchymal transition (EMT) phenomena *in vitro* in human colorectal cancer cells via modifying the Wnt/-catenin and claudin-1 signaling pathways (Hseu et al., 2017). *CLDN7*, with emerging clinical significance, is also a diagnostic marker in the COAD (Wang et al., 2018). *CLDN14*, an upregulated prognostic gene, influence colorectal cancer progression through controlling the PI3K/AKT/mTOR pathway (Qiao et al., 2021). Altogether, it can be stated that the claudins are associated with the diagnostic efficacy in COAD. A changeable biomarker has potential in a common tumor like colorectal cancer. Important issues about the use of such a biomarker in the treatment of cancer still need to be addressed, therefore there is still more work to be done. Regarding the potential utility of claudins as biomarkers of prognostic and therapeutic characteristics, it is evident that there is growing agreement.

## 5 Conclusion

In conclusion, the intricate web of scientific exploration into the identification of key claudin genes and their multifaceted associations with prognosis, immune regulation, signaling regulations, and diagnostic potential holds the promise of ushering in a transformative era in the landscape of colon cancer treatment. The culmination of research efforts reveals a compelling narrative wherein the expression patterns of claudins, with particular emphasis on CLDN5, CLDN11, and CLDN18, emerge as integral players intricately woven into the fabric of immune modulation, cancer-related pathways, malignant phenotypes, and diagnostic precision within the context of COAD.

The nexus between claudin expression and immune regulation signifies a pivotal role in orchestrating the delicate balance between tumor progression and immune response, holding the potential to unlock innovative immunotherapeutic strategies. The intertwining of claudins with cancer-associated pathways further underscores their significance as potential druggable targets, inviting the exploration of targeted interventions to disrupt aberrant signaling cascades.

However, it is imperative to acknowledge that the journey from scientific discovery to clinical translation is a rigorous one, necessitating meticulous experimental validation of these identified key claudins. The validation process stands as a critical bridge, bridging the gap between bench research and bedside application, thereby ensuring the safe and efficacious incorporation of these findings into the realm of colon cancer therapeutics. Rigorous scrutiny and validation are indispensable to establish the credibility and reliability of these observations, ultimately paving the way for their seamless integration into clinical practice.

In essence, the comprehensive studies conducted in unraveling the intricate tapestry of claudin involvement in colon cancer wield the potential to reshape our understanding of the disease and its treatment paradigms. Through the collaborative efforts of researchers, clinicians, and the broader scientific community, the multifaceted functions of claudins shall undoubtedly be harnessed to unveil a new dawn of therapeutic possibilities, advancing the frontiers of colon cancer treatment and, ultimately, augmenting the quality of life for those affected by this formidable disease.

## Figures and Tables

**Table:** 

**AUC**	Area Under The ROC Curve
**Cafs**	Cancer Associated Fibroblasts
**Cams**	Cell Adhesion Molecules
**CEGMR**	Center Of Excellence In Genomic Medicine Research
**COAD**	Colon Adenocarcinoma
**Degs**	Differentially Expressed Genes
**ECM**	Extracellular Matrix
**EGFR**	Epidermal Growth Factor Receptor
**EMT**	Epithelial-To-Mesenchymal Transition
**ERBB**	Epidermal Growth Factor Receptor
**GEPIA**	Gene Expression Profiling Interactive Analysis
**GO**	Gene Ontology
**GSEA**	Gene Set Enrichment Analysis
**KEGG**	Kyoto Encyclopedia Of Genes And Genomes
**MAPK**	Mitogen-Activated Protein Kinase
**MDSC**	Myeloid Derived Suppressor Cells
**MTOR**	Mammalian Target of Rapamycin
**NK**	Natural Killer
**Pdcs**	Plasmacytoid Dendritic Cells
**PPI**	Protein-Protein Interaction
**ROC**	Receiver Operating Characteristic
**ssGSEA**	Survival Analysis, Single Sample Gene Set Enrichment Analysis
**Tams**	Tumor Associated Macrophages
**TCGA**	The Cancer Genome Atlas
**Tfh**	T Follicular Helper Cells
**TGF Beta**	Transforming Growth Factor Beta
**Th17**	T-Helper 17
**Tils**	Tumor Infiltrating Lymphocytes
**Tregs**	Regulatory T Cells
**VEGF**	Vascular Endothelial Growth Factor
**Wnt**	Wingless/Integrated

## Data Availability

The datasets presented in this study can be found in online repositories. The names of the repository/repositories and accession number(s) can be found in the article/Supplementary Material.
