# Supplementary figures and images for: Identification of key claudin genes associated with survival prognosis and diagnosis in colon cancer through integrated bioinformatic analysis
Source: Front Genet. 2023 Sep 19;14:1221815. doi: 10.3389/fgene.2023.1221815 (PMC10550083; doi:10.3389/fgene.2023.1221815)

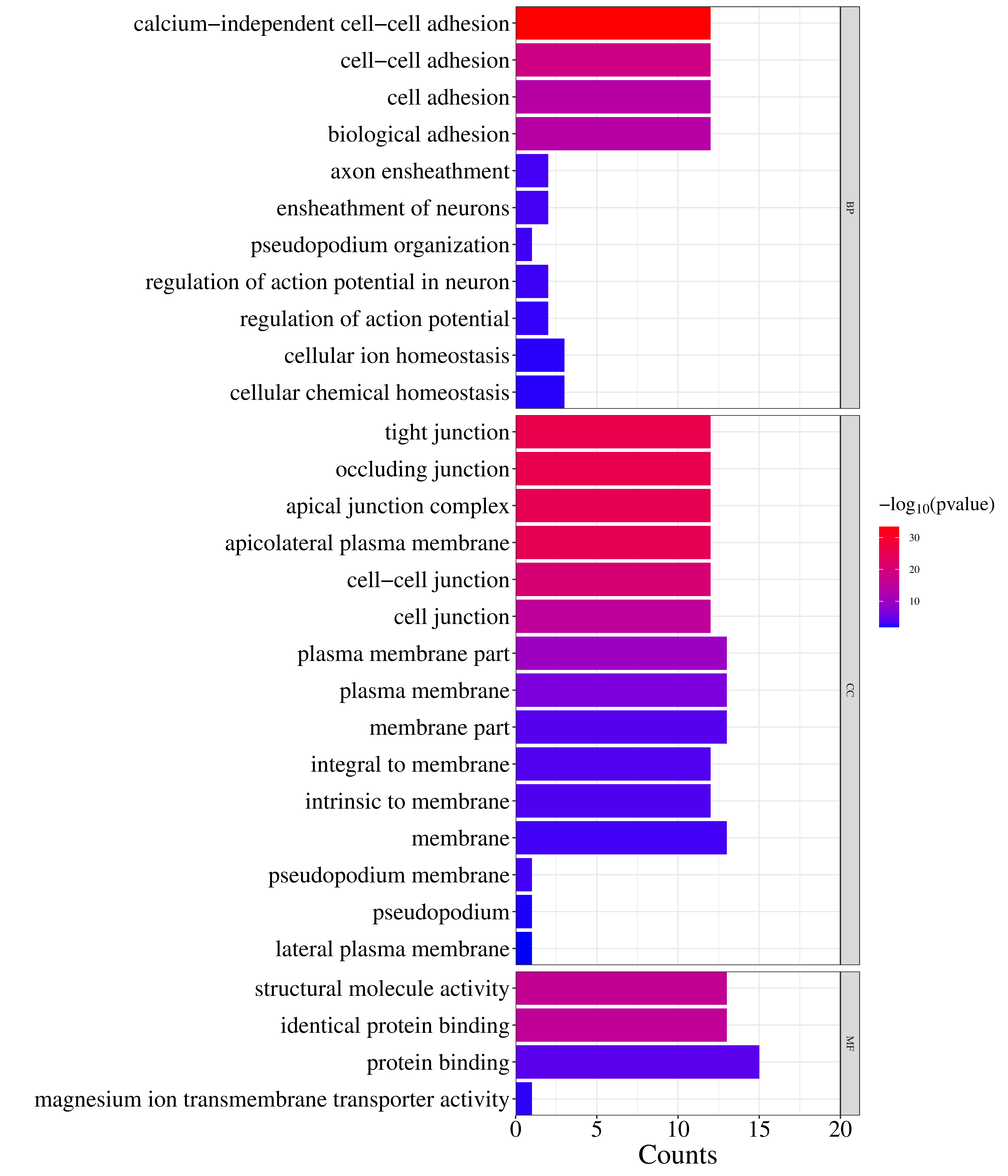

Supplement: Supplementary file 2 [file DataSheet3.ZIP › Zip S6/BINGO and DAVID/60d13a6a74a12068.tiff]

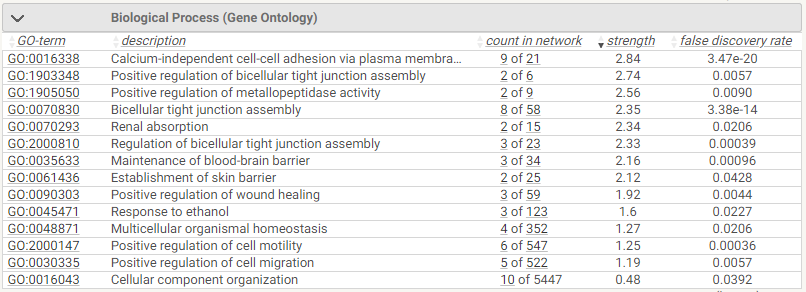

Supplement: Supplementary file 2 [file DataSheet3.ZIP › Zip S6/BINGO and DAVID/BIOLOGICAL_PROCESS.PNG]

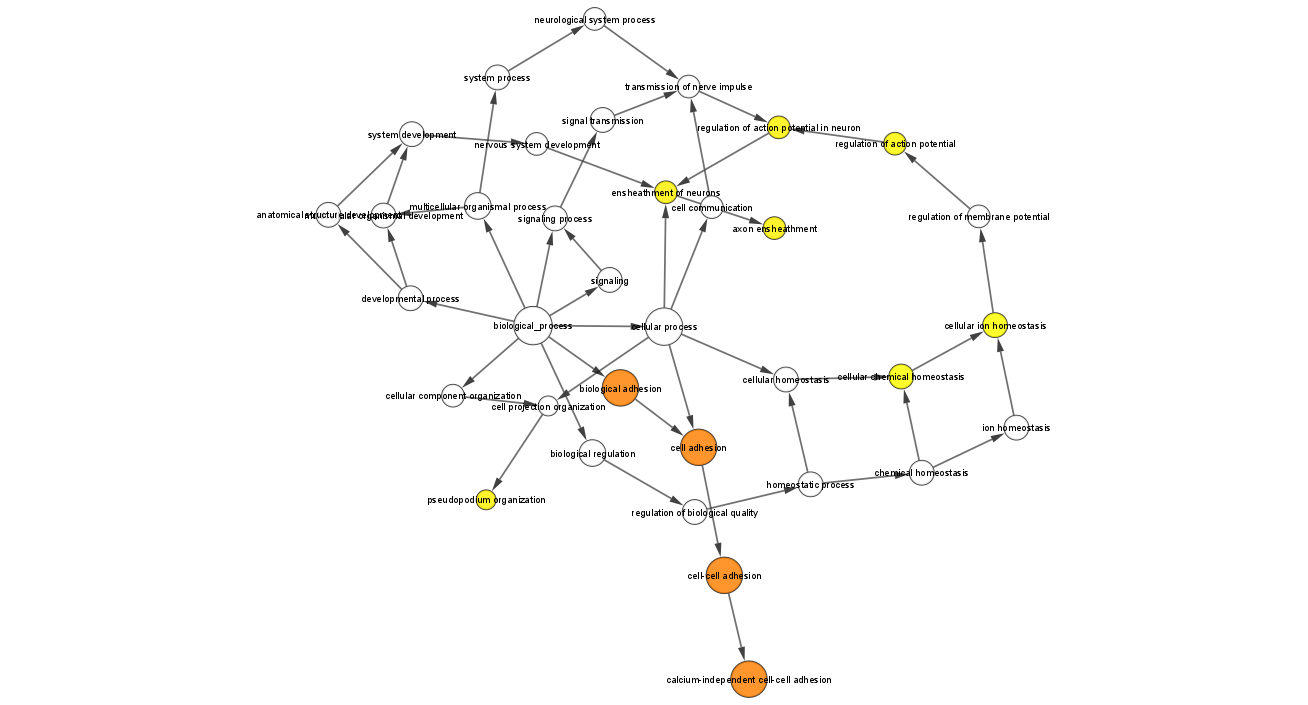

Supplement: Supplementary file 2 [file DataSheet3.ZIP › Zip S6/BINGO and DAVID/BP.png]

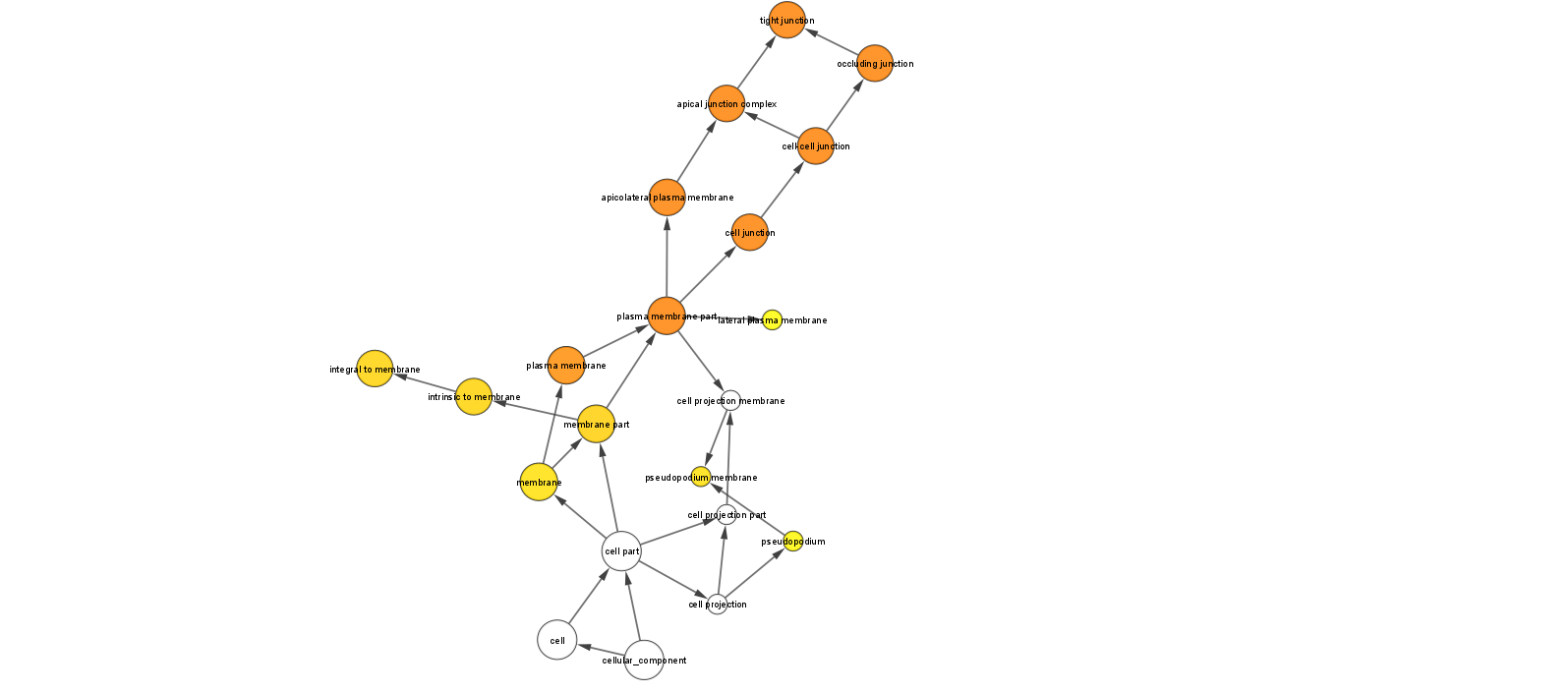

Supplement: Supplementary file 2 [file DataSheet3.ZIP › Zip S6/BINGO and DAVID/CC.png]

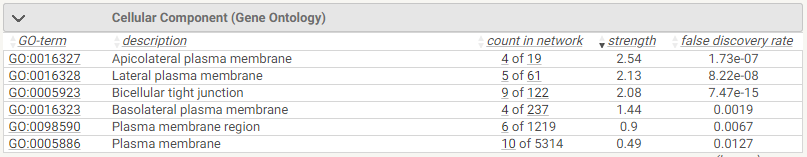

Supplement: Supplementary file 2 [file DataSheet3.ZIP › Zip S6/BINGO and DAVID/CELLULAR_COMPONENT.PNG]

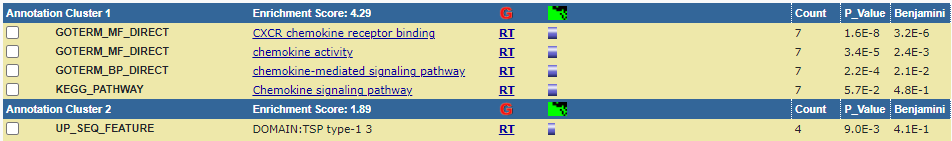

Supplement: Supplementary file 2 [file DataSheet3.ZIP › Zip S6/BINGO and DAVID/DOWNREGULATED_GENES_PATHWAYS.PNG]

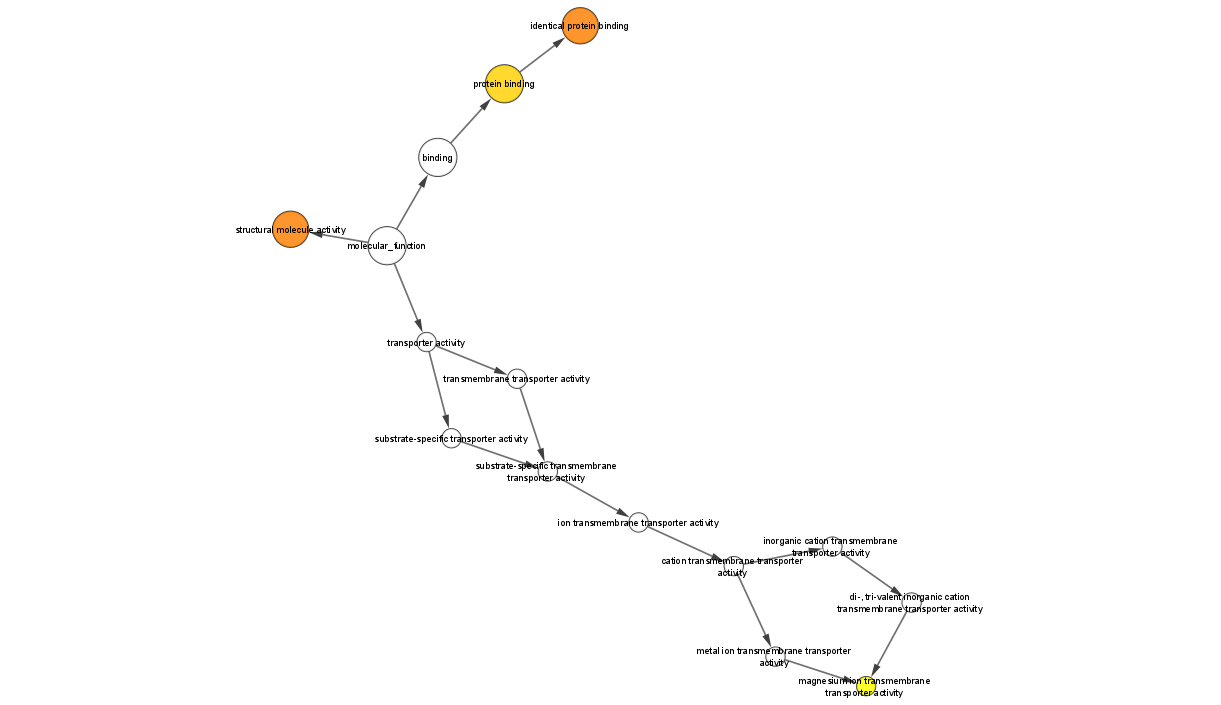

Supplement: Supplementary file 2 [file DataSheet3.ZIP › Zip S6/BINGO and DAVID/MF.png]

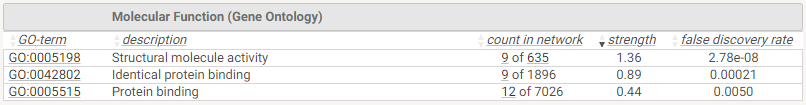

Supplement: Supplementary file 2 [file DataSheet3.ZIP › Zip S6/BINGO and DAVID/MOLECULAR_FUNCTION.PNG]

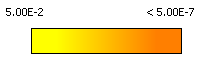

Supplement: Supplementary file 2 [file DataSheet3.ZIP › Zip S6/BINGO and DAVID/SCALE.PNG]

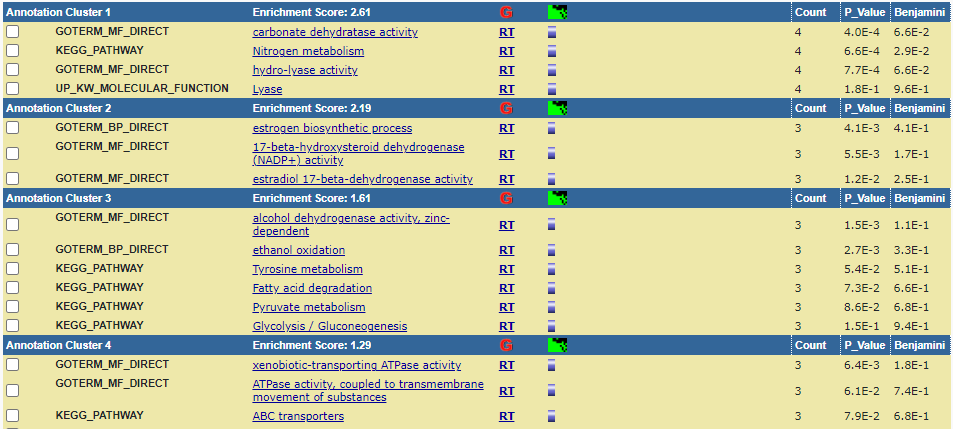

Supplement: Supplementary file 2 [file DataSheet3.ZIP › Zip S6/BINGO and DAVID/UPREGULATED_GENES_PATHWAYS.PNG]

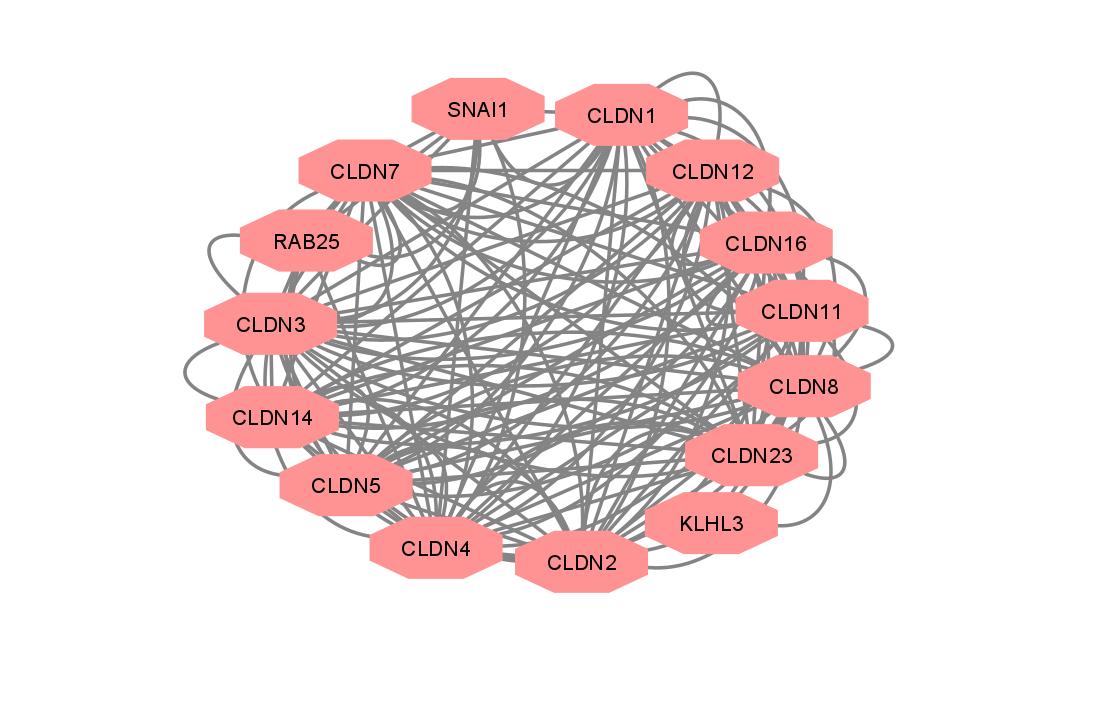

Supplement: Supplementary file 2 [file DataSheet3.ZIP › Zip S6/CYTOSCAPE/CLAUDINS.jpeg]

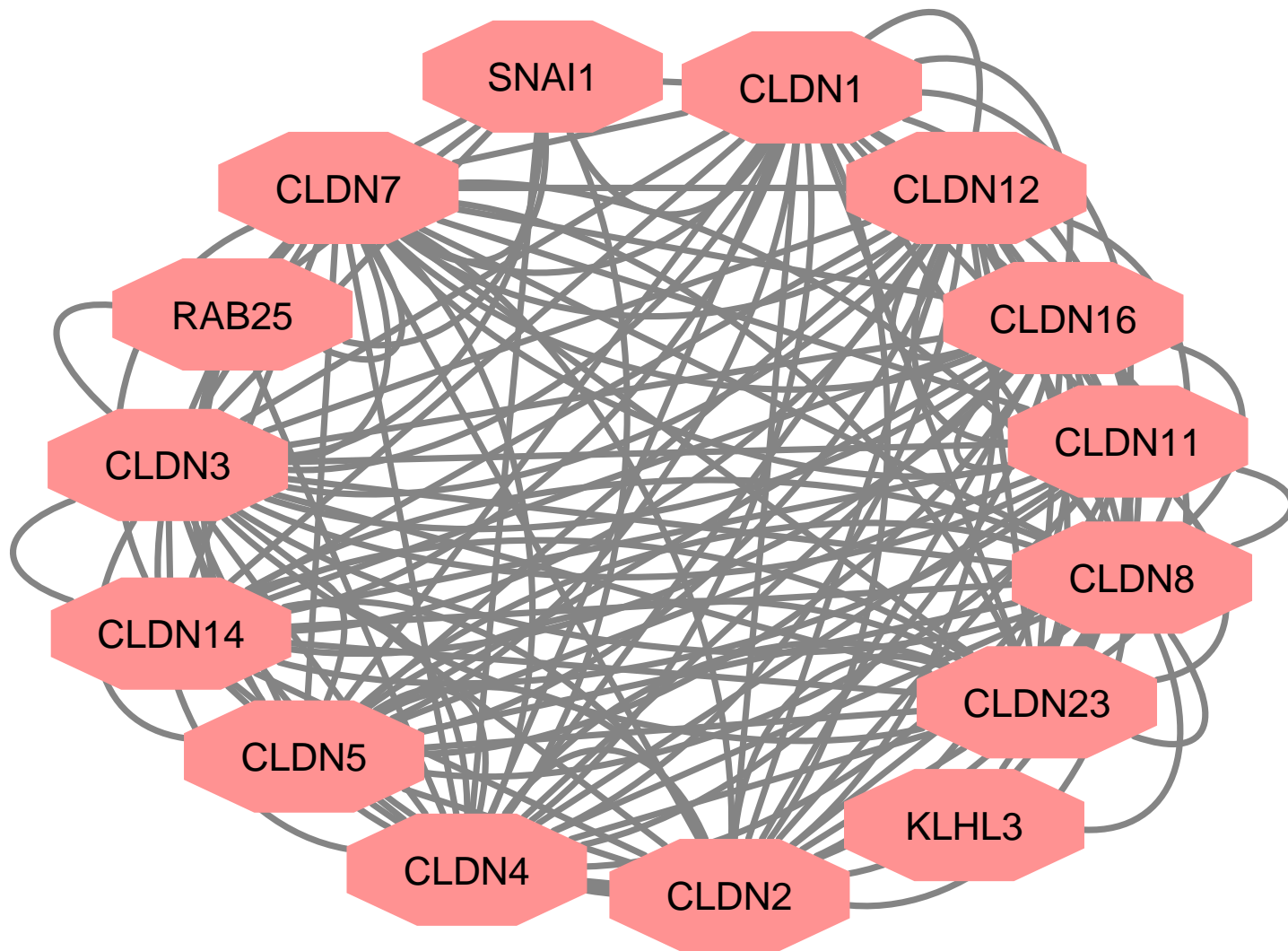

Supplement: Supplementary file 2 [file DataSheet3.ZIP › Zip S6/CYTOSCAPE/CLAUDINS.pdf]

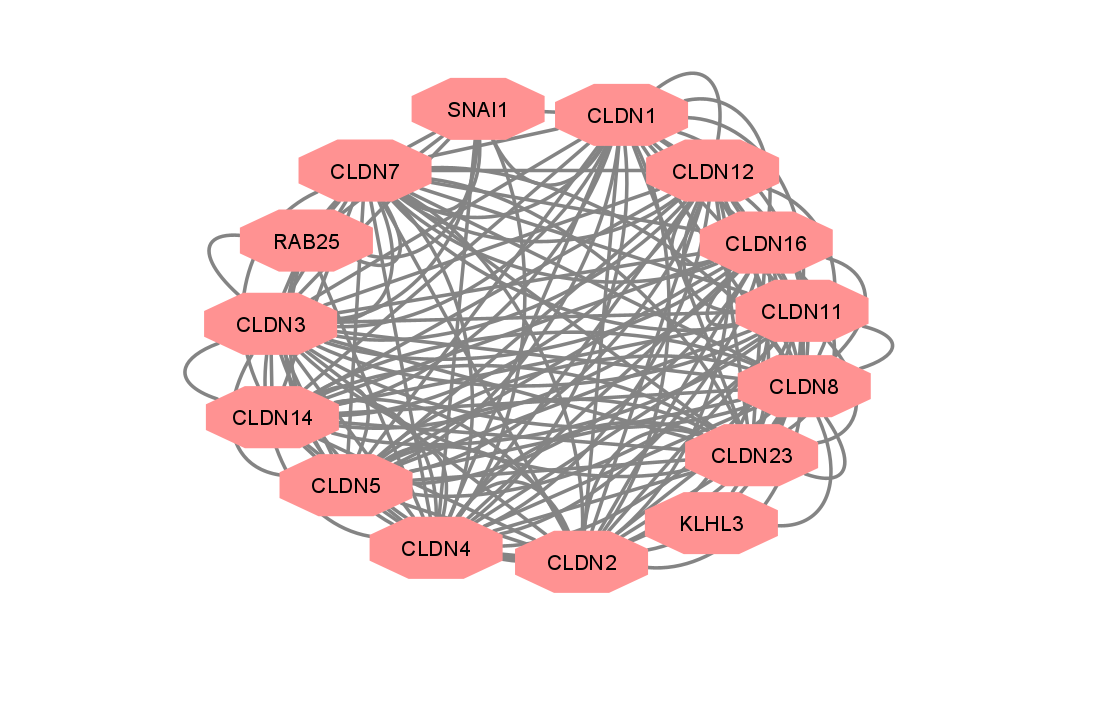

Supplement: Supplementary file 2 [file DataSheet3.ZIP › Zip S6/CYTOSCAPE/CLAUDINS.png]

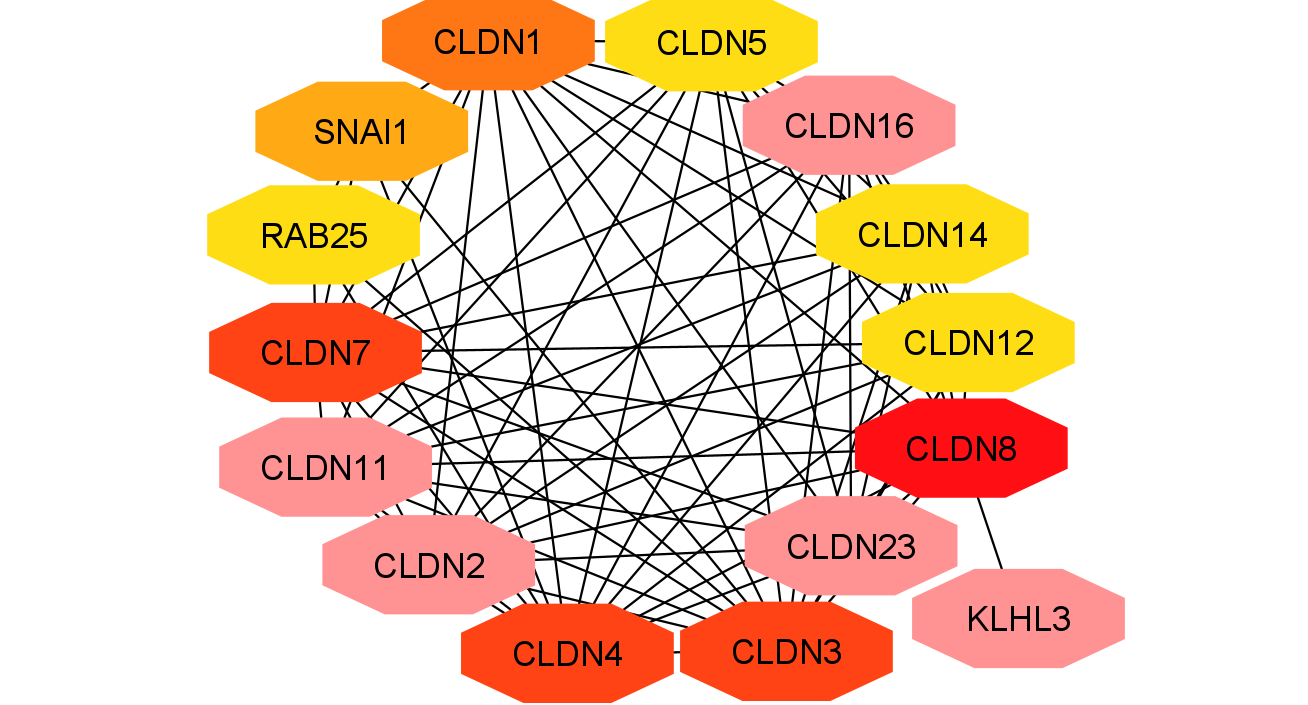

Supplement: Supplementary file 2 [file DataSheet3.ZIP › Zip S6/CYTOSCAPE/Sheet1_Betweenness_top10_with_neighbors_and_expanded.png]

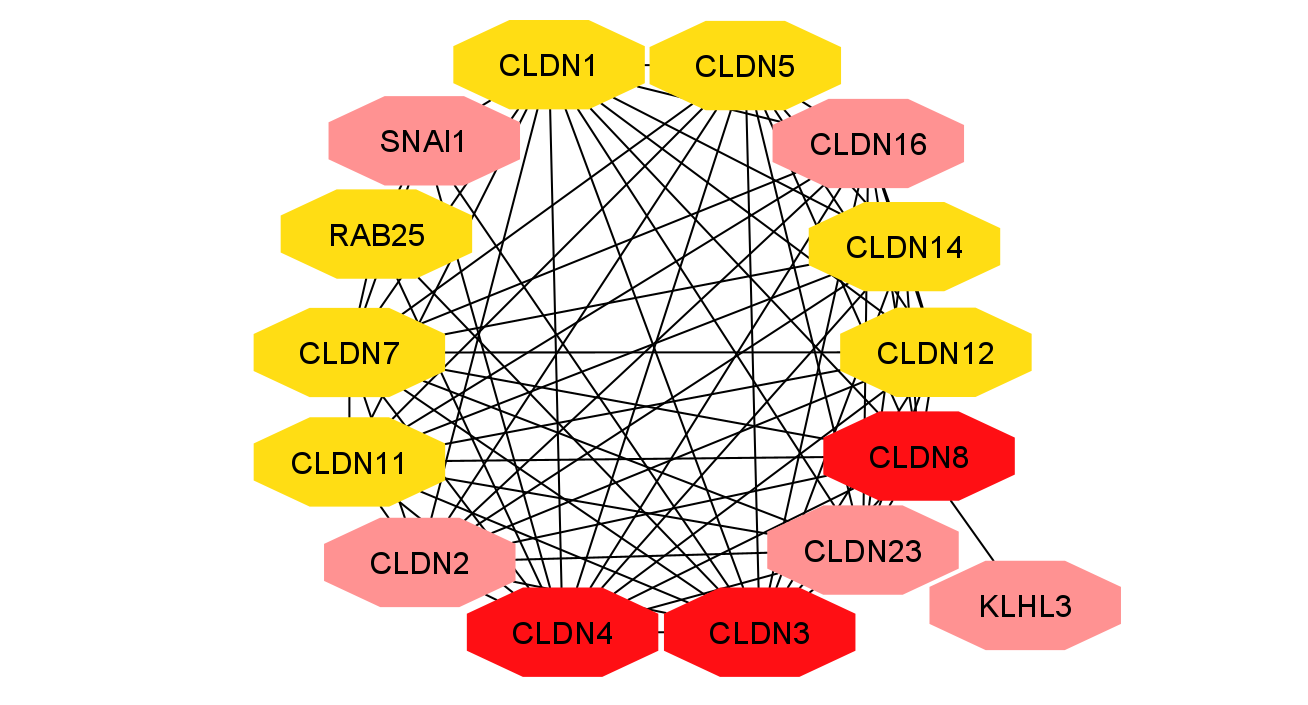

Supplement: Supplementary file 2 [file DataSheet3.ZIP › Zip S6/CYTOSCAPE/Sheet1_BottleNeck_top10_with_neighbors_and_expanded.png]

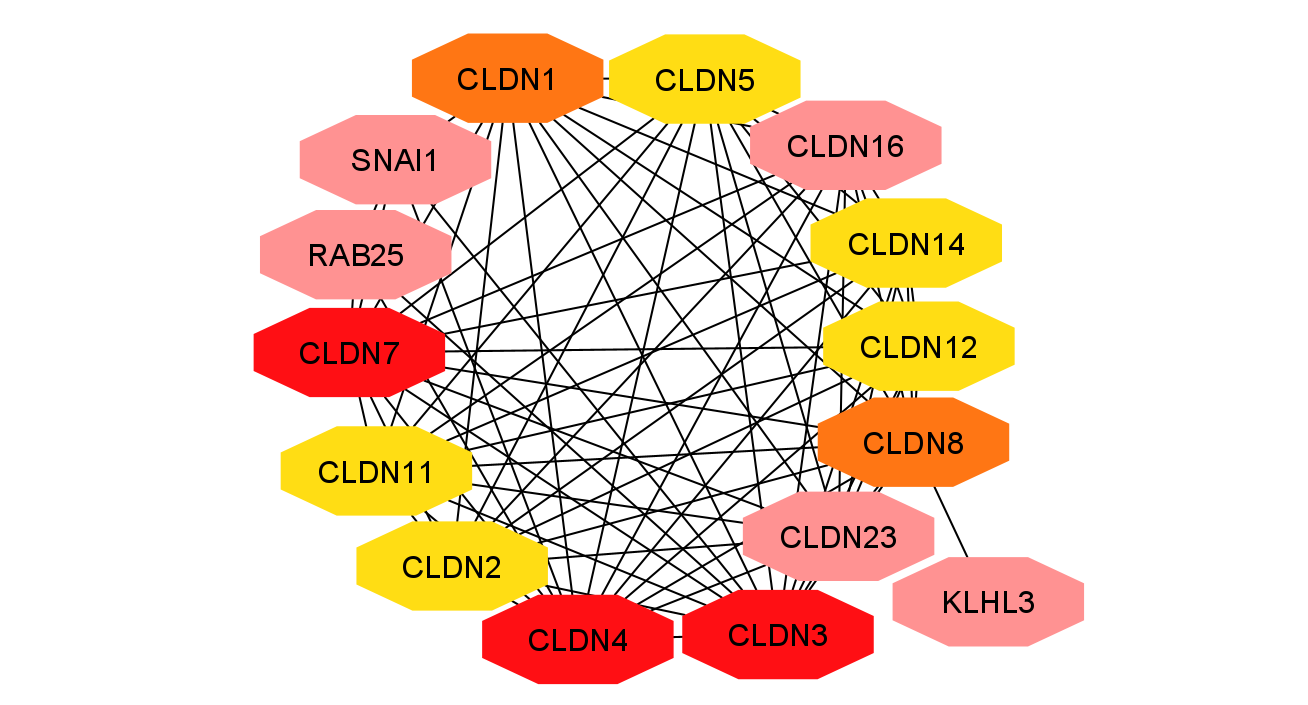

Supplement: Supplementary file 2 [file DataSheet3.ZIP › Zip S6/CYTOSCAPE/Sheet1_Closeness_top10_with_neighbors_and_expanded.png]

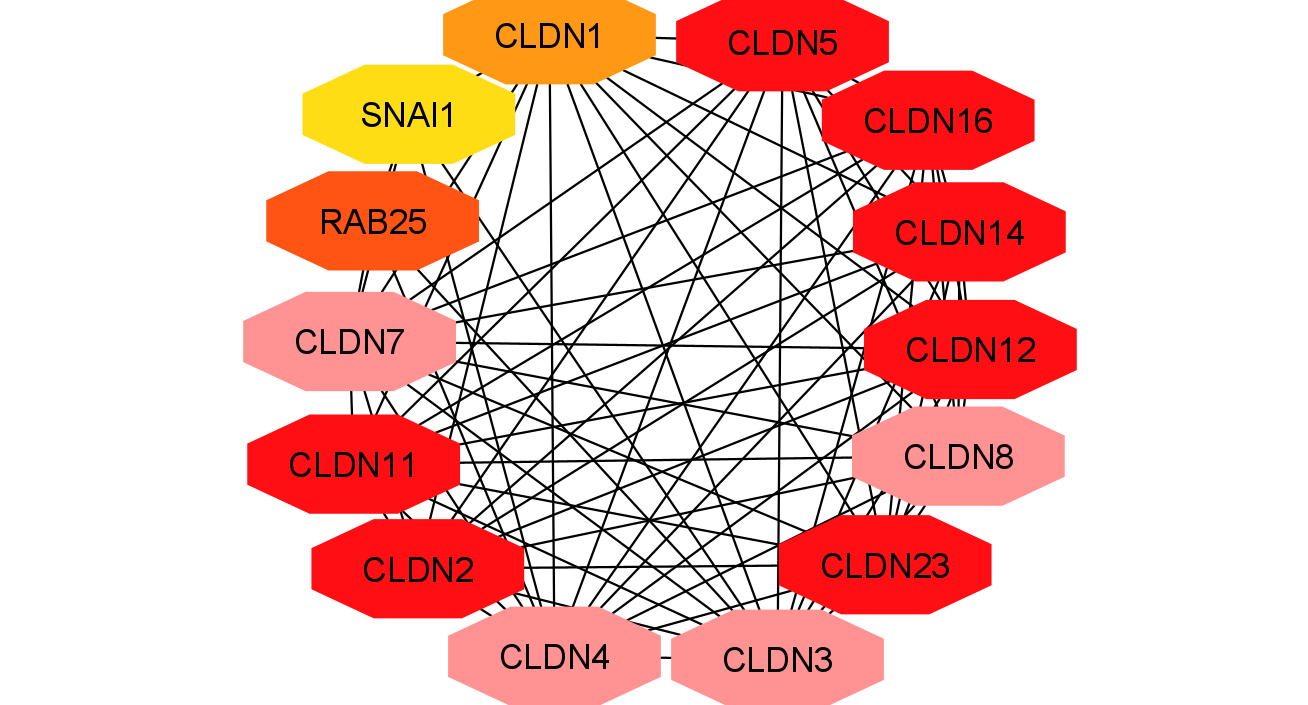

Supplement: Supplementary file 2 [file DataSheet3.ZIP › Zip S6/CYTOSCAPE/Sheet1_ClusteringCoefficient_top10_with_neighbors_and_expanded.png]

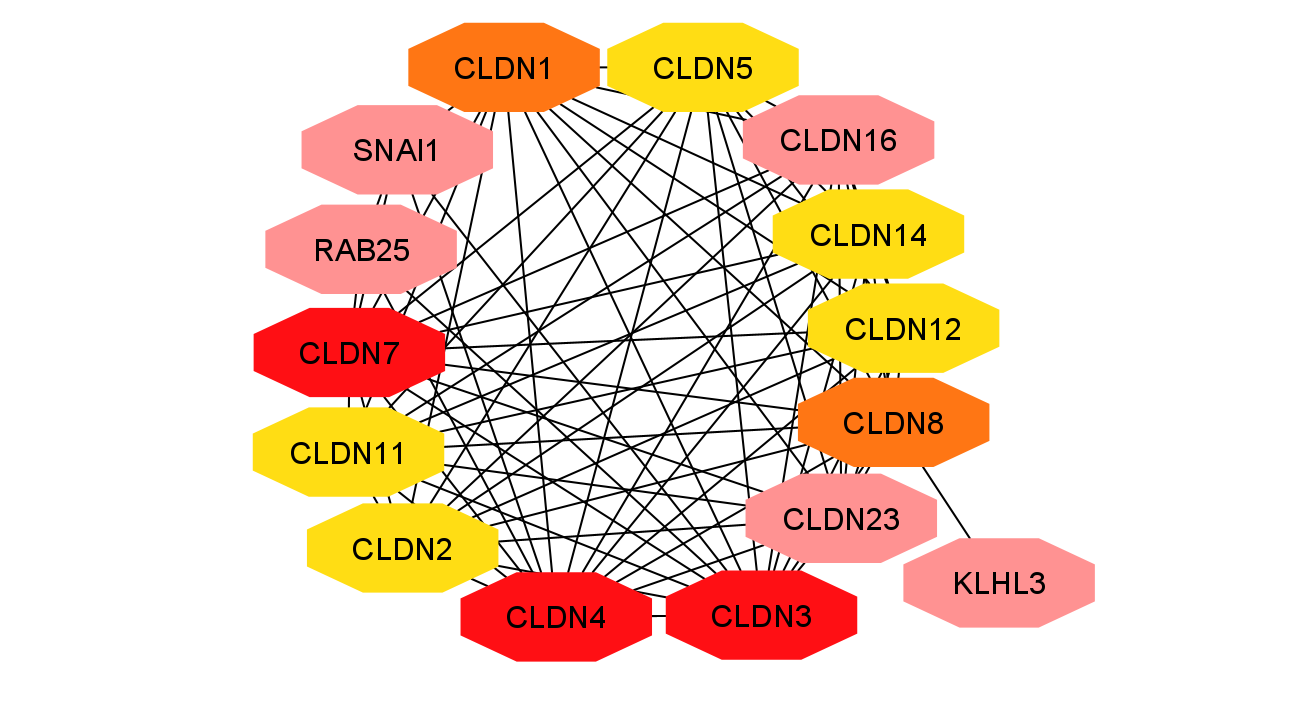

Supplement: Supplementary file 2 [file DataSheet3.ZIP › Zip S6/CYTOSCAPE/Sheet1_Degree_top10_with_neighbors_and_expanded.png]

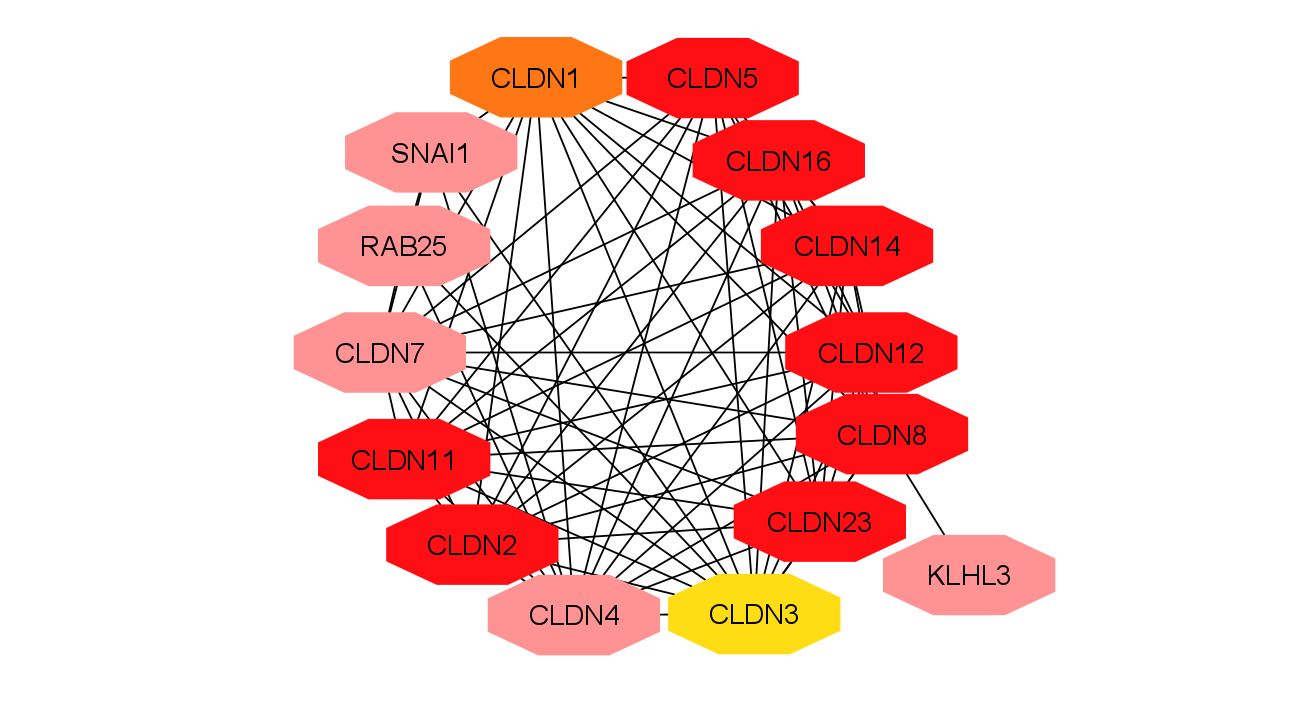

Supplement: Supplementary file 2 [file DataSheet3.ZIP › Zip S6/CYTOSCAPE/Sheet1_DMNC_top10_with_neighbors_and_expanded.png]

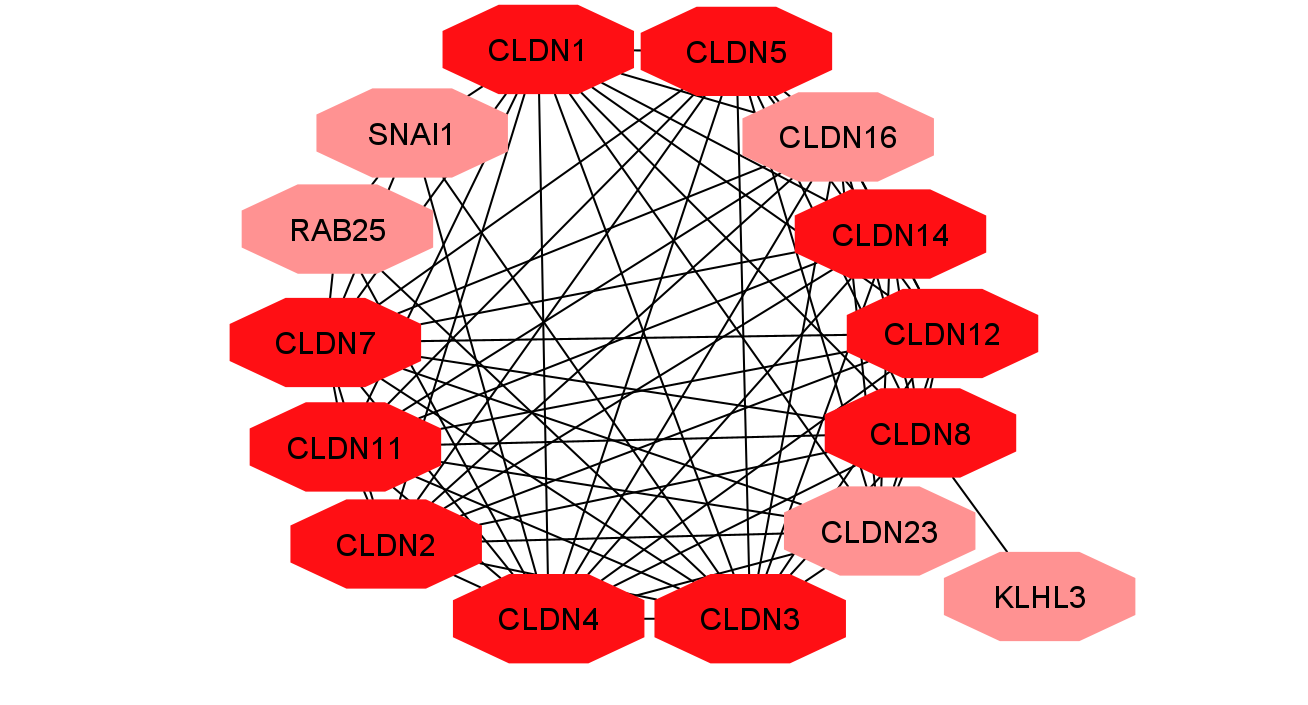

Supplement: Supplementary file 2 [file DataSheet3.ZIP › Zip S6/CYTOSCAPE/Sheet1_EcCentricity_top10_with_neighbors_and_expanded.png]

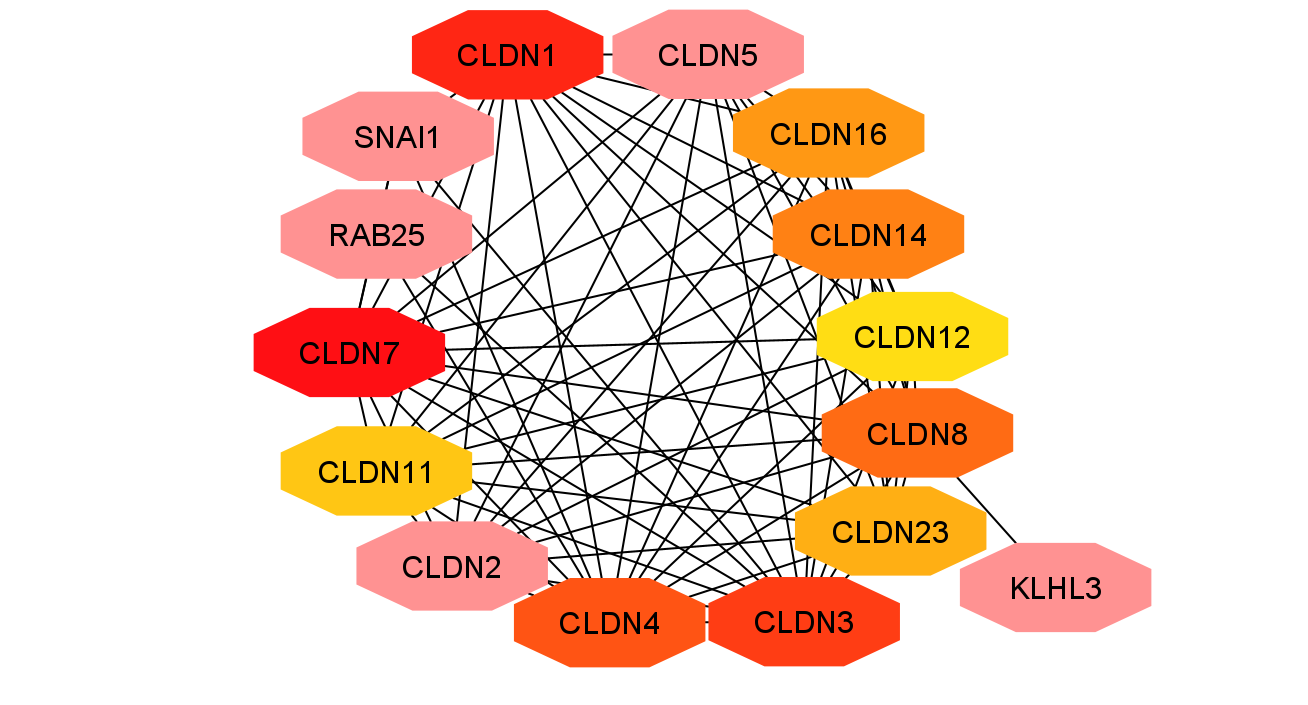

Supplement: Supplementary file 2 [file DataSheet3.ZIP › Zip S6/CYTOSCAPE/Sheet1_EPC_top10_with_neighbors_and_expanded.png]

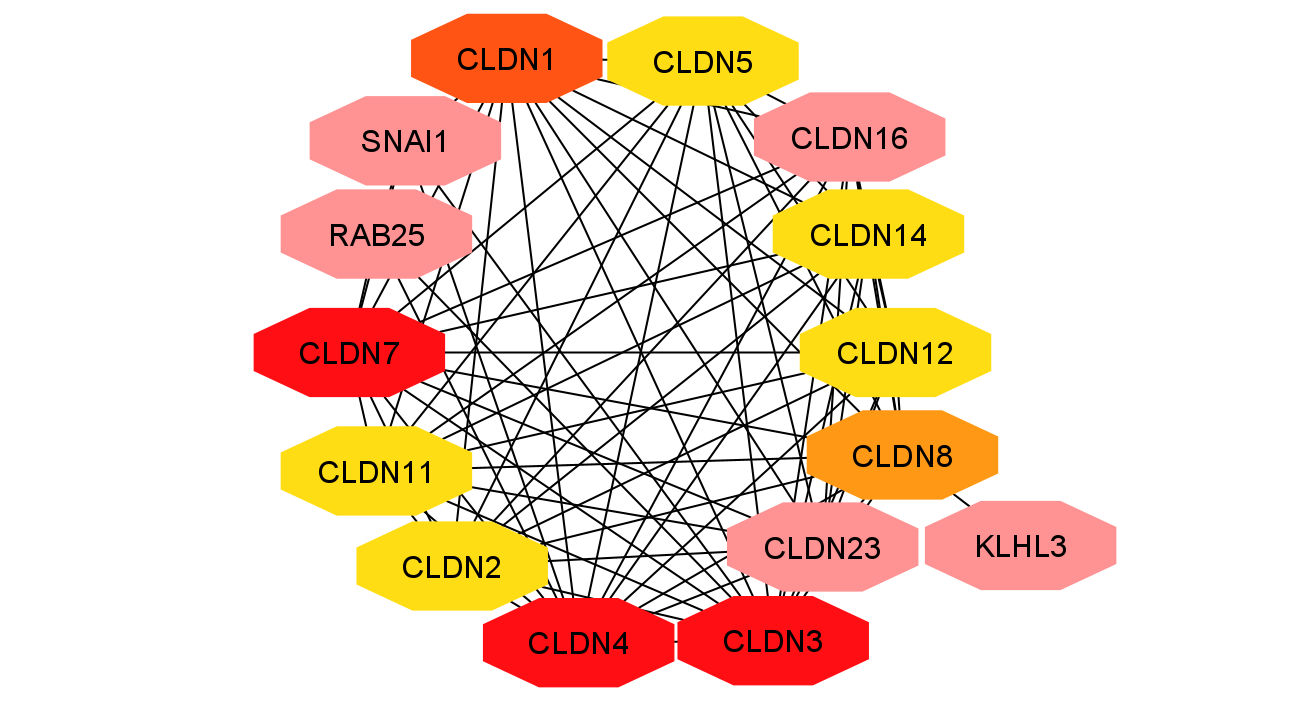

Supplement: Supplementary file 2 [file DataSheet3.ZIP › Zip S6/CYTOSCAPE/Sheet1_MCC_top10_with_neighbors_and_expanded.png]

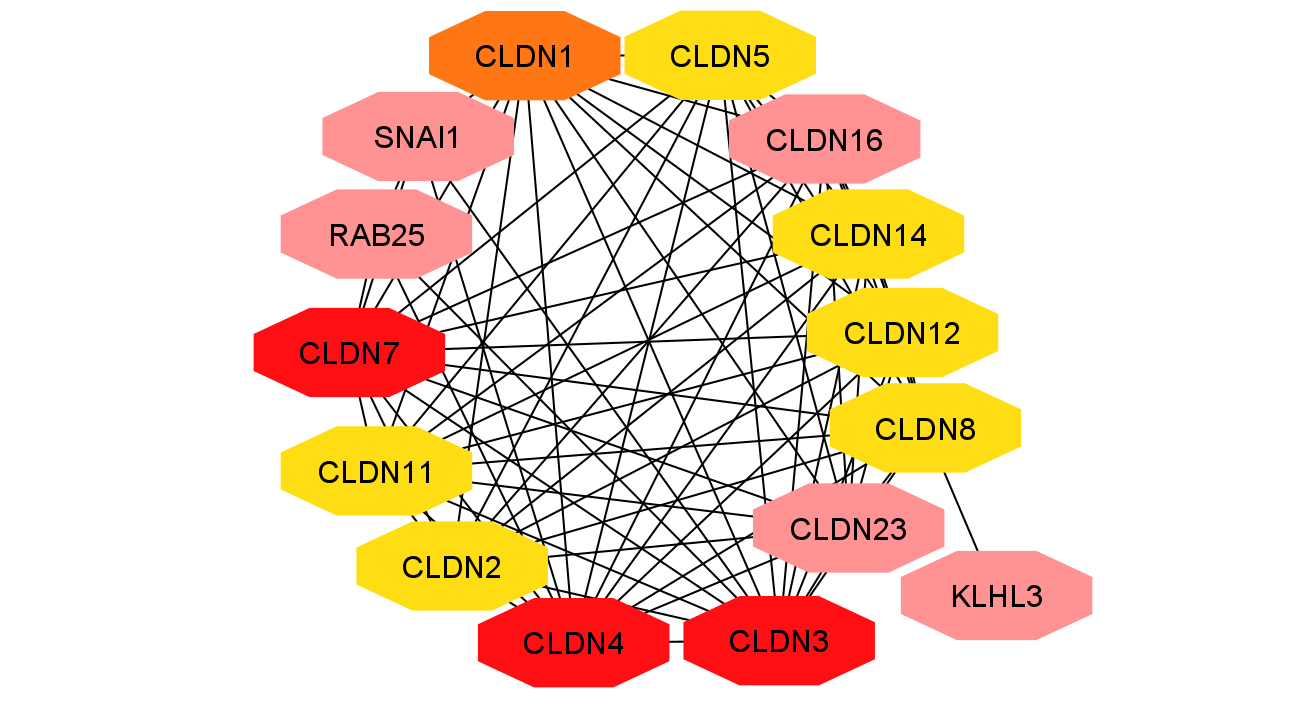

Supplement: Supplementary file 2 [file DataSheet3.ZIP › Zip S6/CYTOSCAPE/Sheet1_MNC_top10_with_neighbors_and_expanded.png]

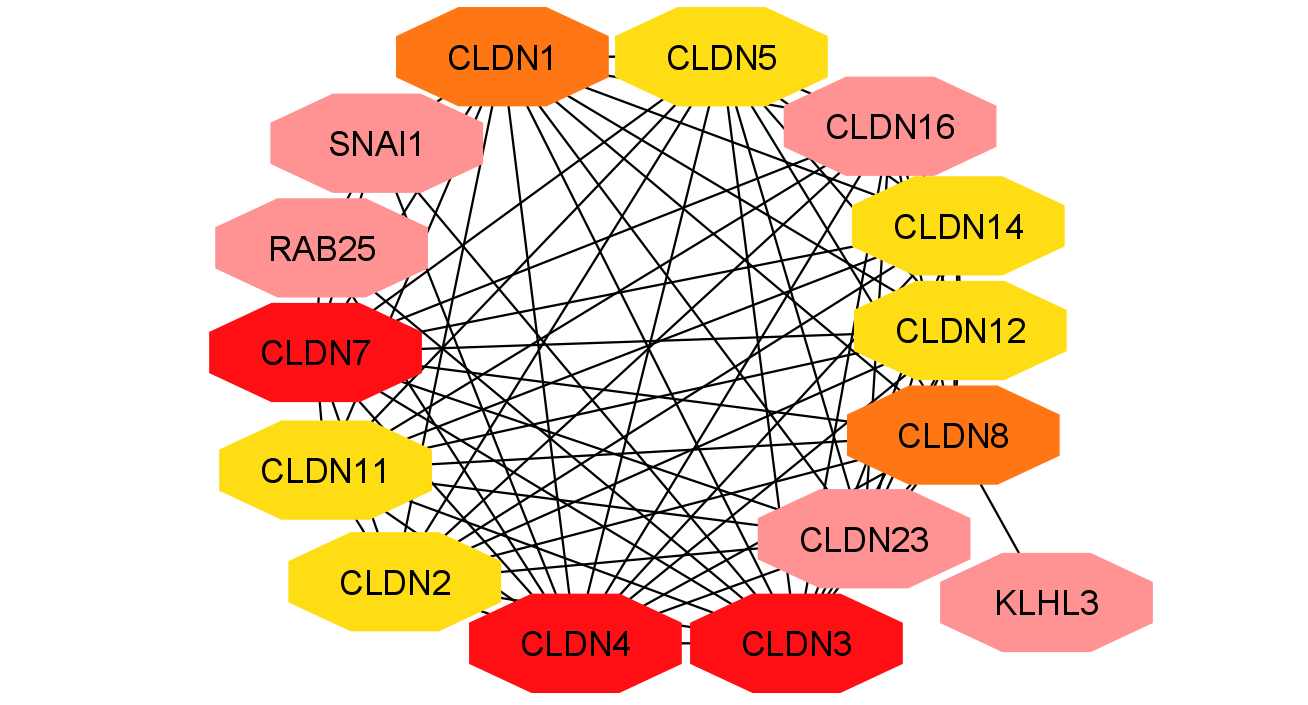

Supplement: Supplementary file 2 [file DataSheet3.ZIP › Zip S6/CYTOSCAPE/Sheet1_Radiality_top10_with_neighbors_and_expanded.png]

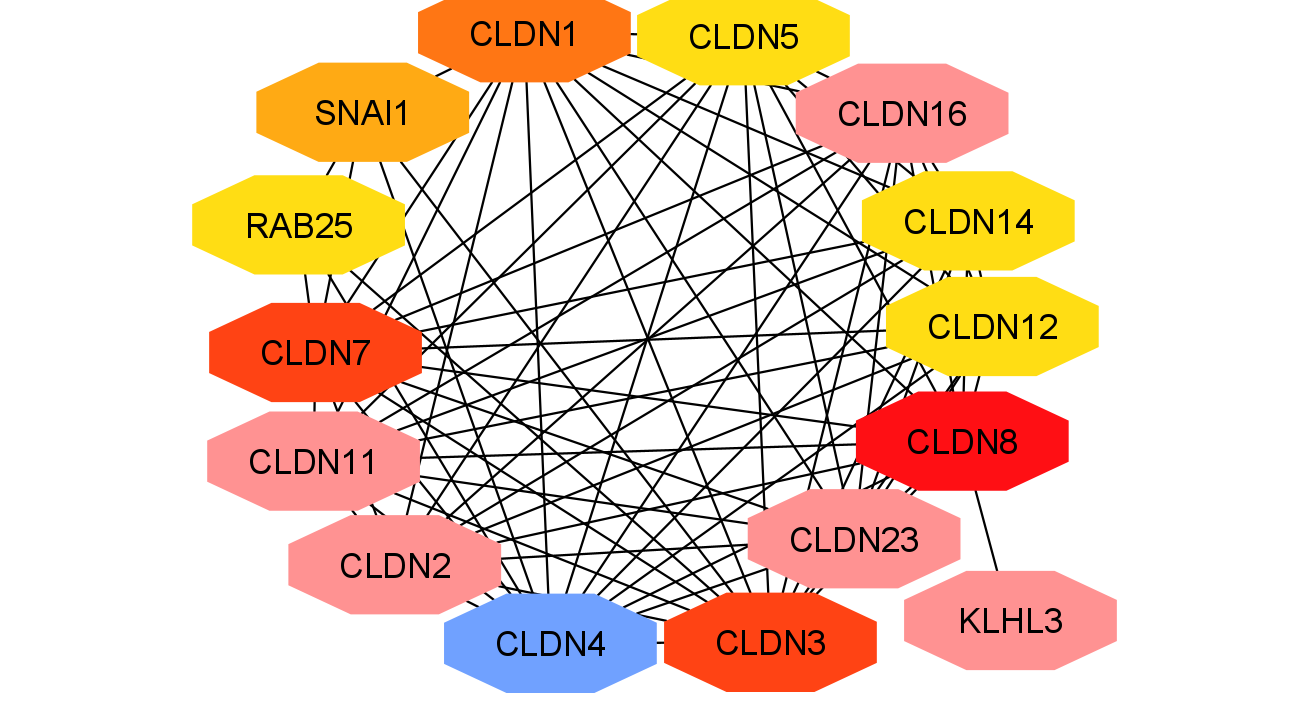

Supplement: Supplementary file 2 [file DataSheet3.ZIP › Zip S6/CYTOSCAPE/Sheet1_Stress_top10_with_neighbors_and_expanded.png]

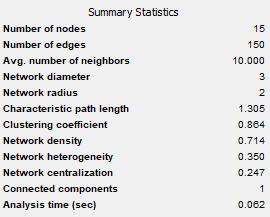

Supplement: Supplementary file 2 [file DataSheet3.ZIP › Zip S6/CYTOSCAPE/SUMMARY STATISTICS.PNG]

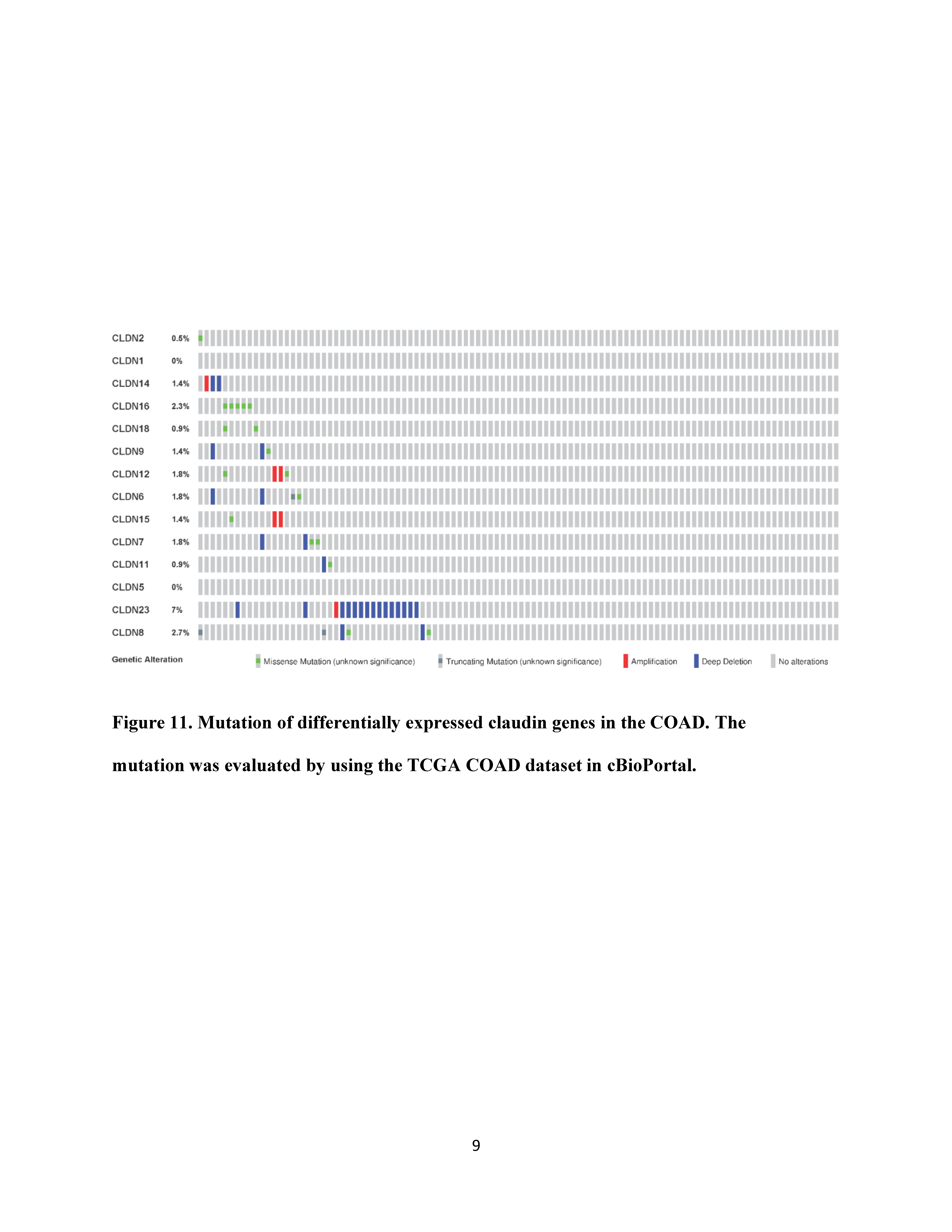

Supplement: Supplementary file 3 [file Figure11.TIFF]

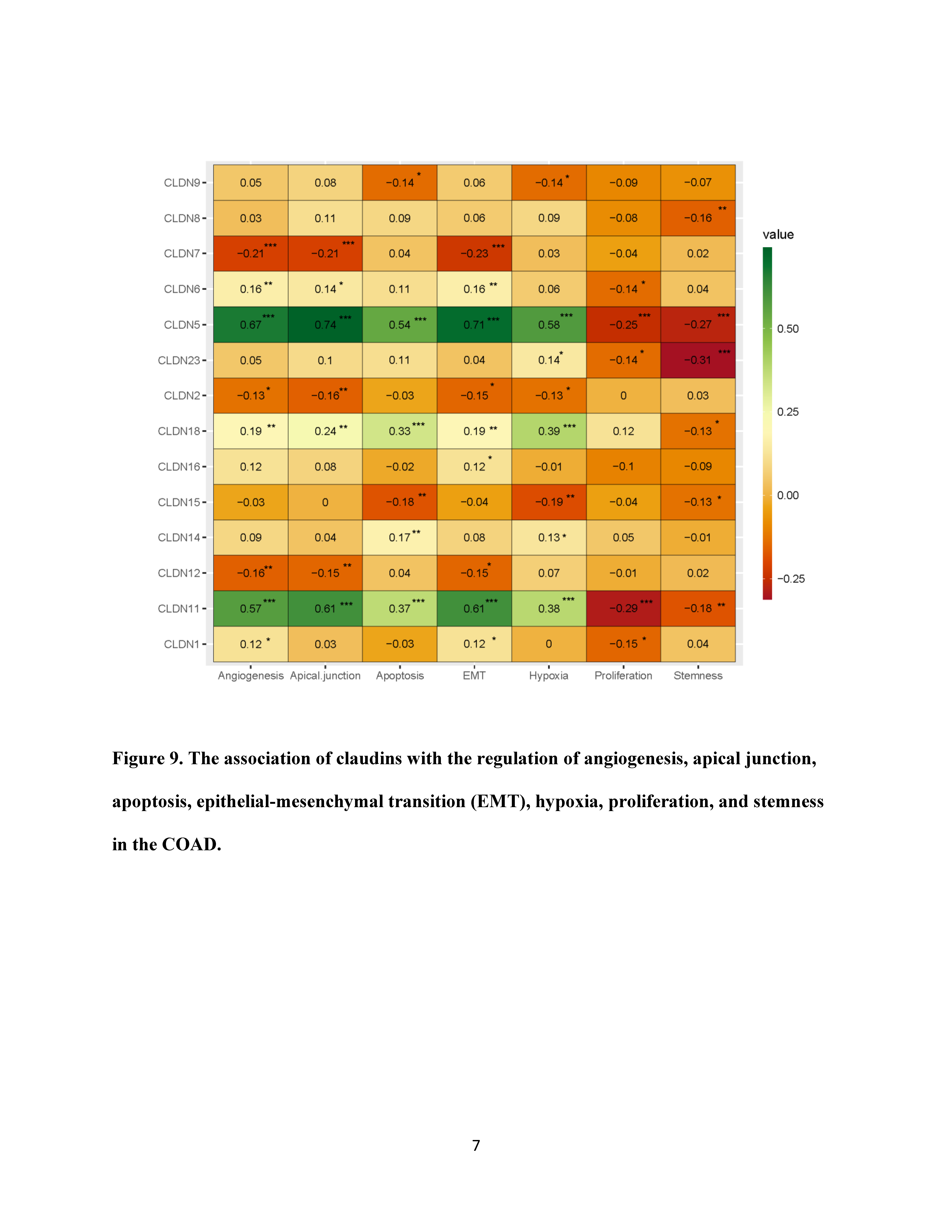

Supplement: Supplementary file 4 [file Figure9.TIFF]

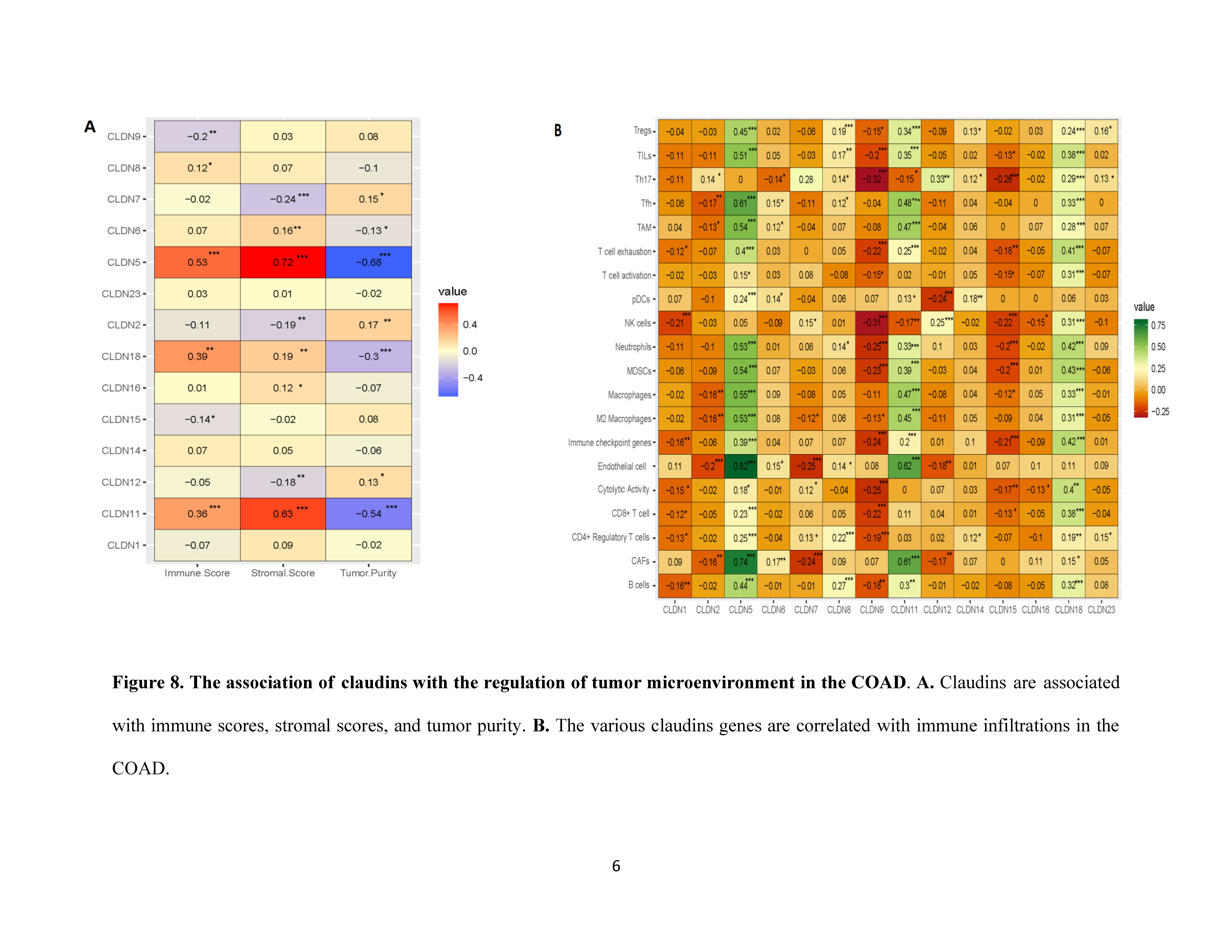

Supplement: Supplementary file 5 [file Figure8.TIFF]

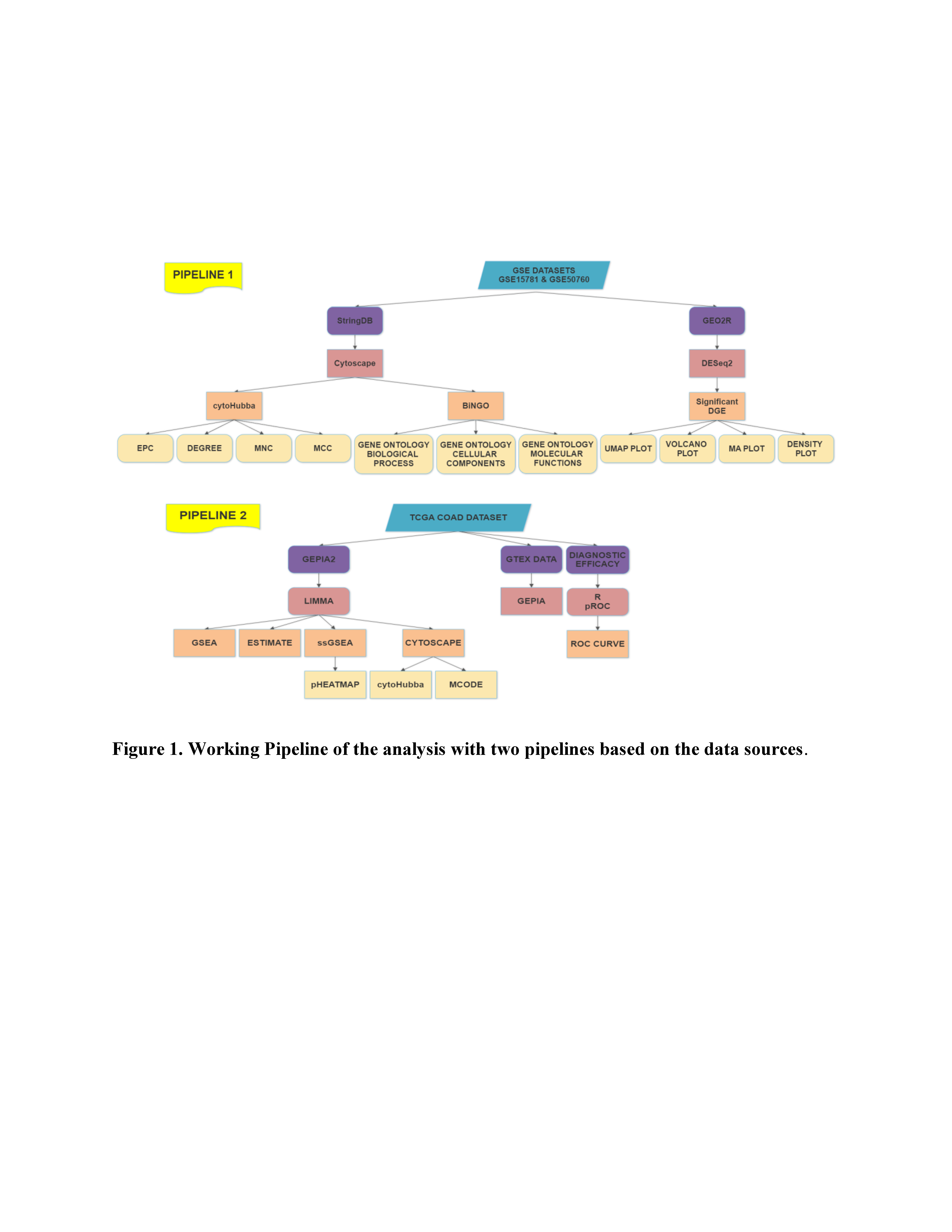

Supplement: Supplementary file 6 [file Figure1.TIFF]

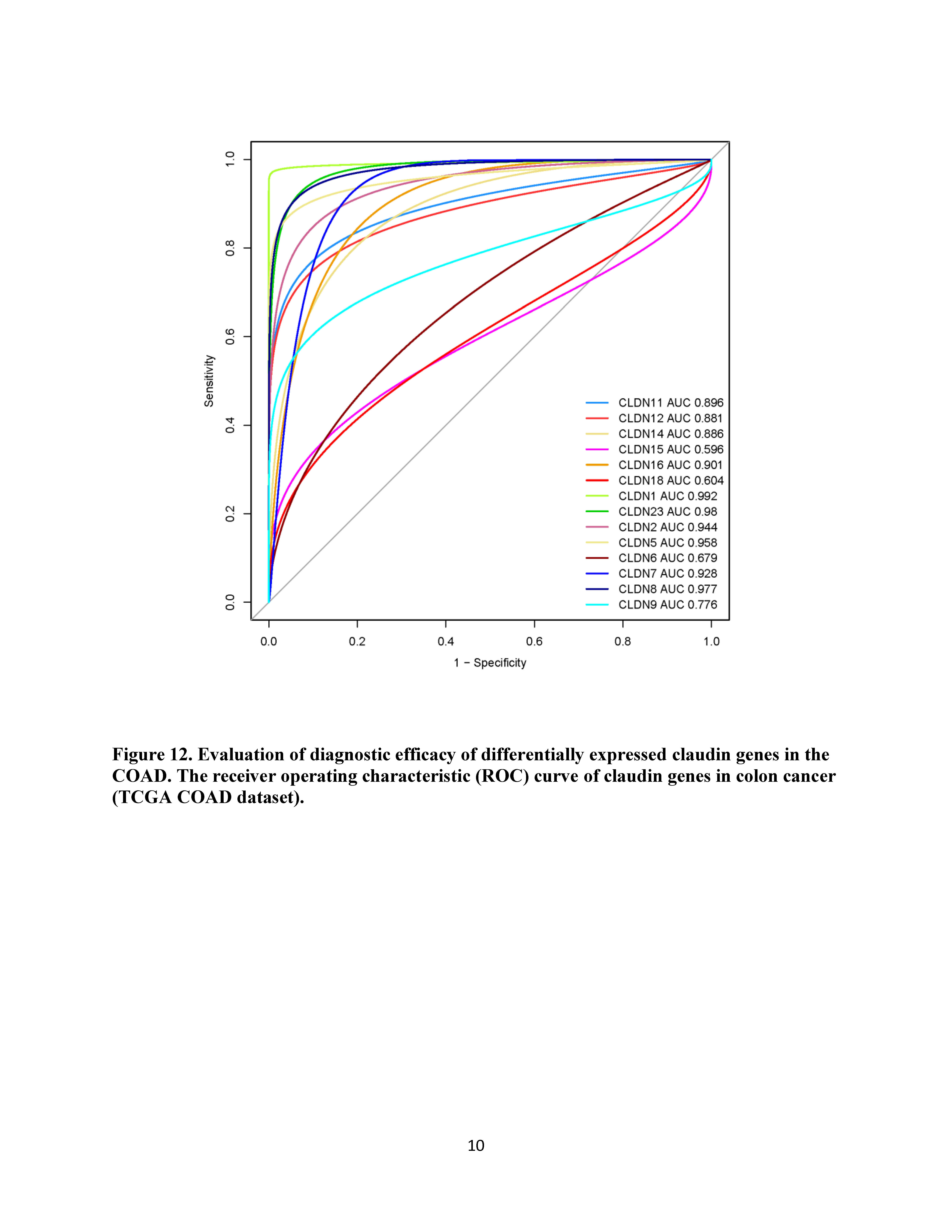

Supplement: Supplementary file 7 [file Figure12.TIFF]

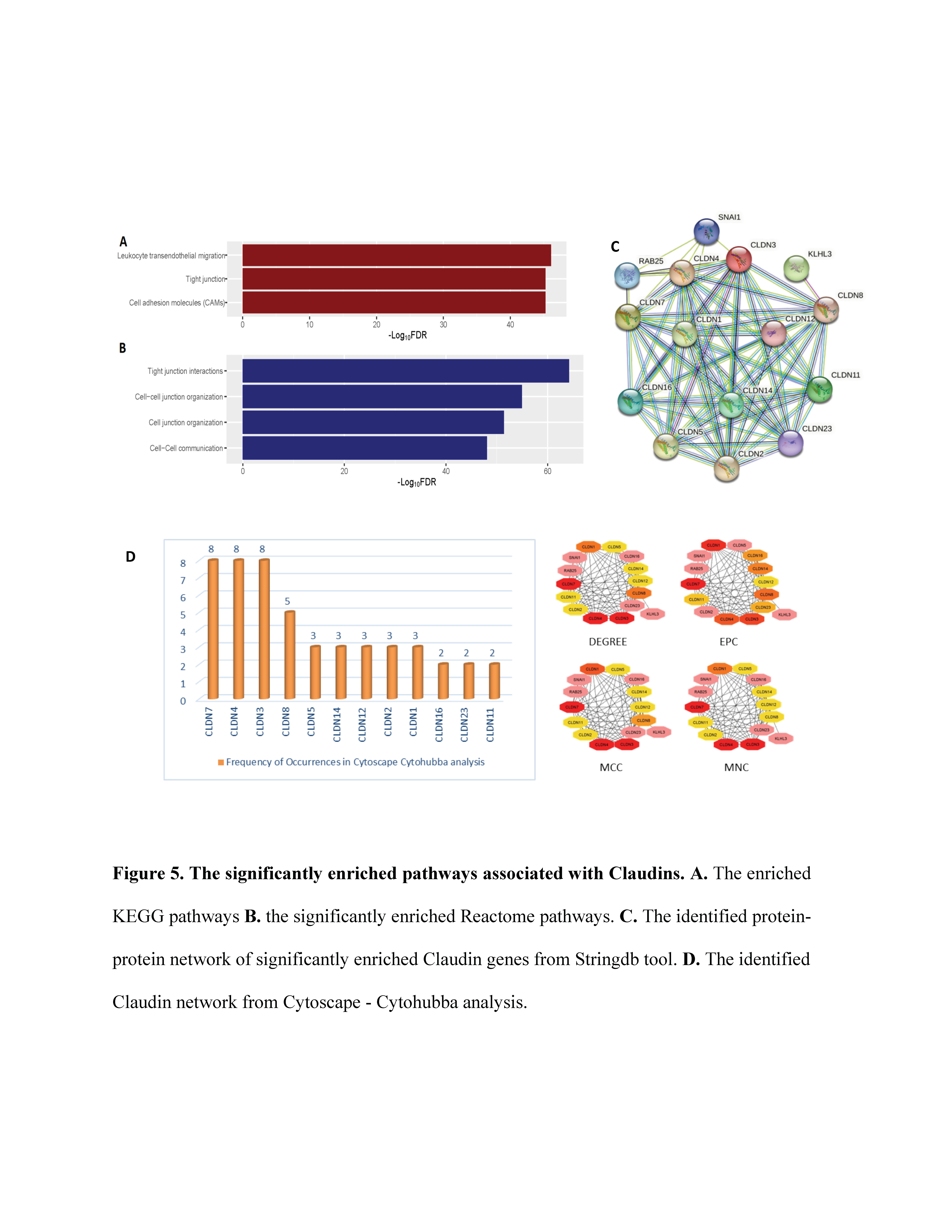

Supplement: Supplementary file 8 [file Figure5.TIFF]

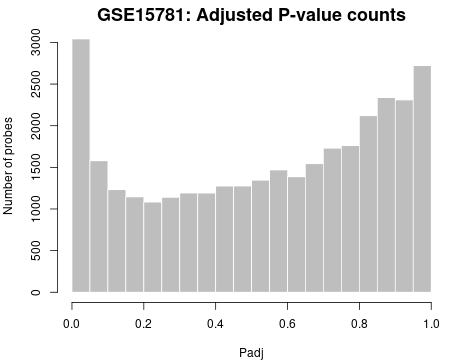

Supplement: Supplementary file 10 [file DataSheet2.ZIP › Zip S5/GSE15781/GSE15781.ADJ.PVALUEPLOT.jfif]

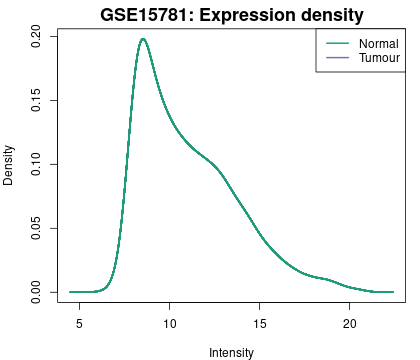

Supplement: Supplementary file 10 [file DataSheet2.ZIP › Zip S5/GSE15781/GSE15781.EXP.DENSITYPLOT.jfif]

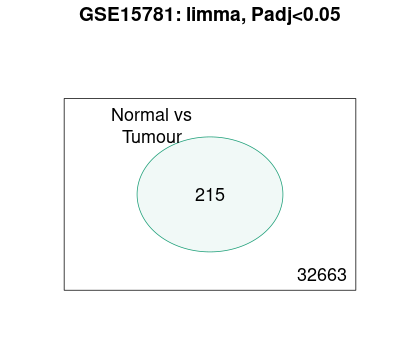

Supplement: Supplementary file 10 [file DataSheet2.ZIP › Zip S5/GSE15781/GSE15781.LIMMAPLOT.jfif]

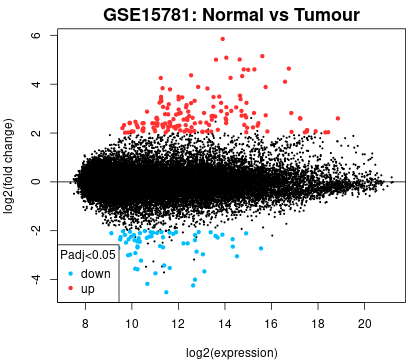

Supplement: Supplementary file 10 [file DataSheet2.ZIP › Zip S5/GSE15781/GSE15781.MAPLOT.jfif]

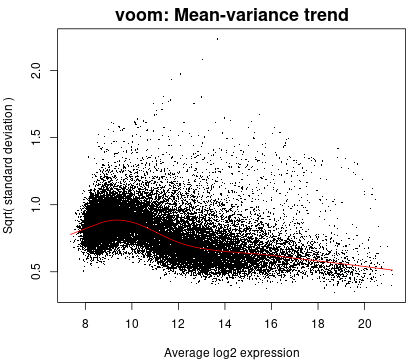

Supplement: Supplementary file 10 [file DataSheet2.ZIP › Zip S5/GSE15781/GSE15781.MEAN-VAR.PLOT.jfif]

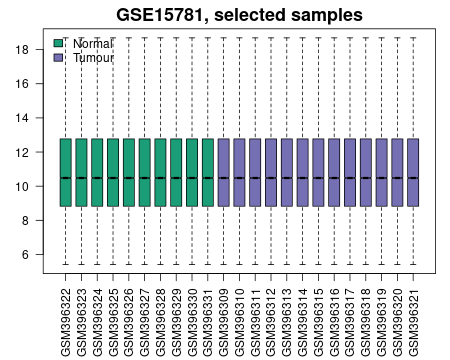

Supplement: Supplementary file 10 [file DataSheet2.ZIP › Zip S5/GSE15781/GSE15781.SAMPLEDIST.PLOT.jfif]

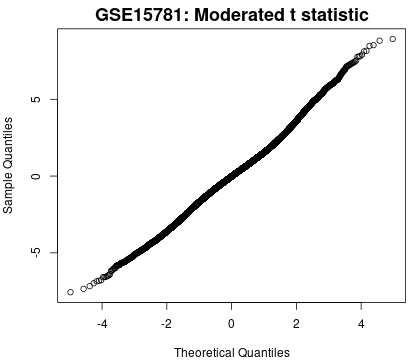

Supplement: Supplementary file 10 [file DataSheet2.ZIP › Zip S5/GSE15781/GSE15781.TSTATISTICSPLOT.jfif]

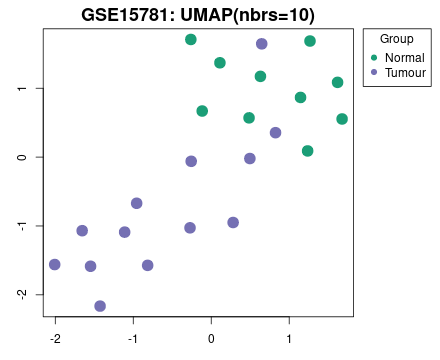

Supplement: Supplementary file 10 [file DataSheet2.ZIP › Zip S5/GSE15781/GSE15781.UMAPPLOT.jfif]

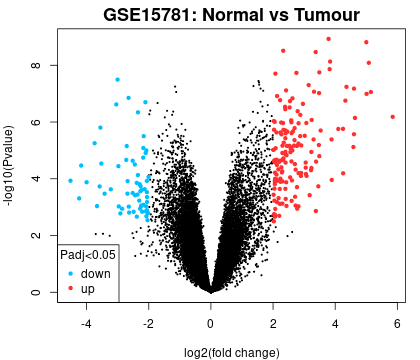

Supplement: Supplementary file 10 [file DataSheet2.ZIP › Zip S5/GSE15781/GSE15781.VOLCANOPLOT.jfif]

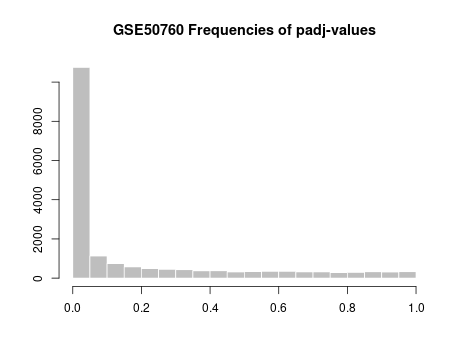

Supplement: Supplementary file 10 [file DataSheet2.ZIP › Zip S5/GSE50760/NORMAL vs METASTASIS/GSE50760.ADJ.PVALUEPLOT.jfif]

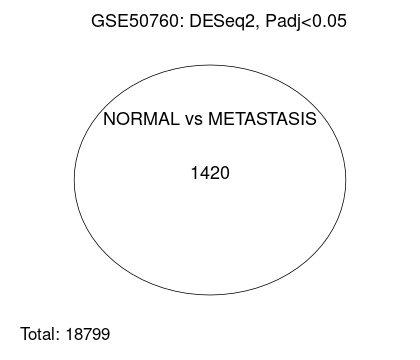

Supplement: Supplementary file 10 [file DataSheet2.ZIP › Zip S5/GSE50760/NORMAL vs METASTASIS/GSE50760.LIMMAPLOT.jfif]

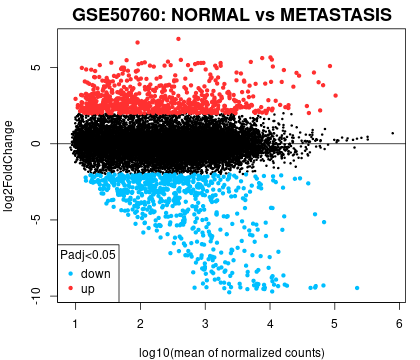

Supplement: Supplementary file 10 [file DataSheet2.ZIP › Zip S5/GSE50760/NORMAL vs METASTASIS/GSE50760.MAPLOT.jfif]

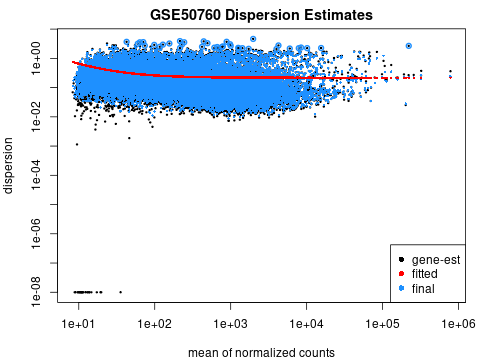

Supplement: Supplementary file 10 [file DataSheet2.ZIP › Zip S5/GSE50760/NORMAL vs METASTASIS/GSE50760.MEAN-VAR.PLOT.jfif]

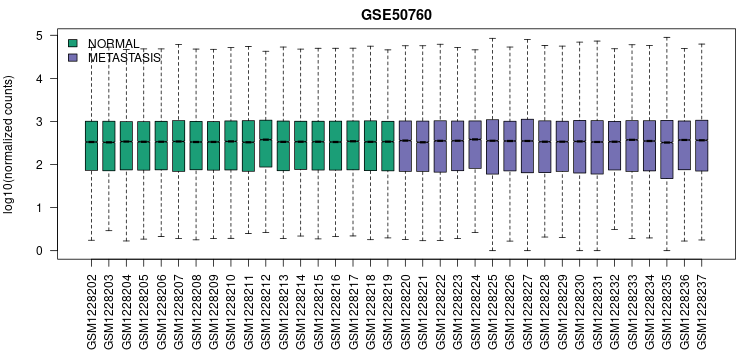

Supplement: Supplementary file 10 [file DataSheet2.ZIP › Zip S5/GSE50760/NORMAL vs METASTASIS/GSE50760.SAMPLEDIST.PLOT.jfif]

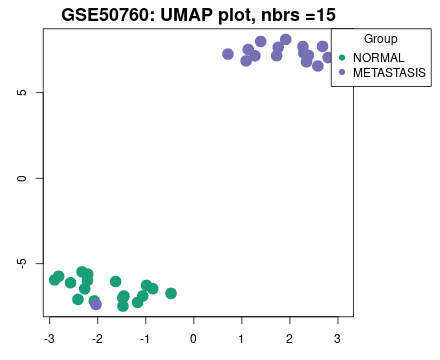

Supplement: Supplementary file 10 [file DataSheet2.ZIP › Zip S5/GSE50760/NORMAL vs METASTASIS/GSE50760.UMAPPLOT.jfif]

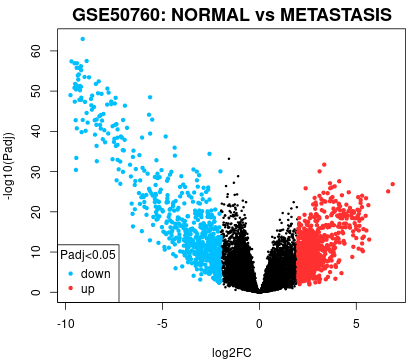

Supplement: Supplementary file 10 [file DataSheet2.ZIP › Zip S5/GSE50760/NORMAL vs METASTASIS/GSE50760.VOLCANOPLOT.jfif]

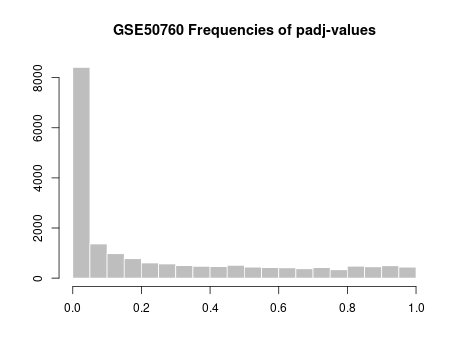

Supplement: Supplementary file 10 [file DataSheet2.ZIP › Zip S5/GSE50760/NORMAL vs TUMOUR/GSE50760.ADJ.PVALUEPLOT.jfif]

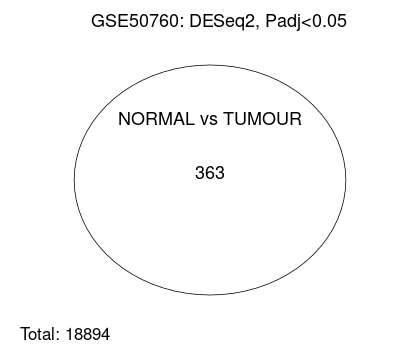

Supplement: Supplementary file 10 [file DataSheet2.ZIP › Zip S5/GSE50760/NORMAL vs TUMOUR/GSE50760.LIMMAPLOT.jfif]

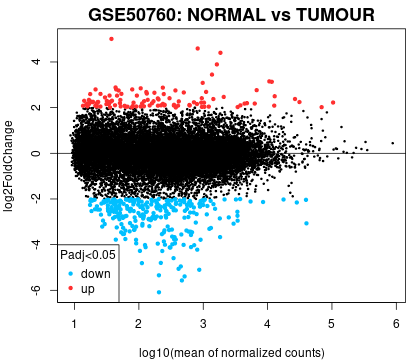

Supplement: Supplementary file 10 [file DataSheet2.ZIP › Zip S5/GSE50760/NORMAL vs TUMOUR/GSE50760.MAPLOT.jfif]

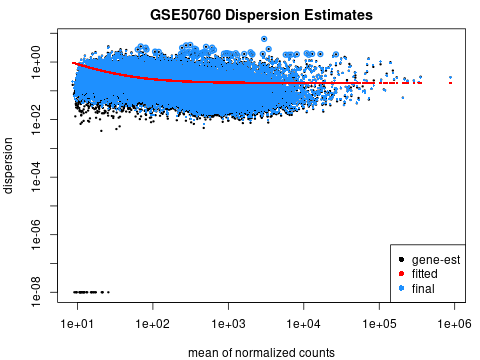

Supplement: Supplementary file 10 [file DataSheet2.ZIP › Zip S5/GSE50760/NORMAL vs TUMOUR/GSE50760.MEAN-VAR.PLOT.jfif]

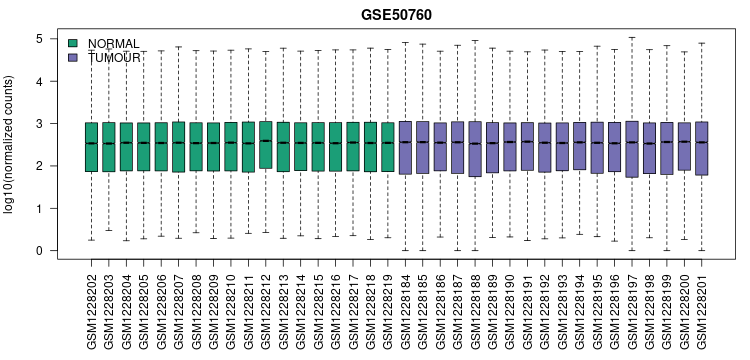

Supplement: Supplementary file 10 [file DataSheet2.ZIP › Zip S5/GSE50760/NORMAL vs TUMOUR/GSE50760.SAMPLEDIST.PLOT.jfif]

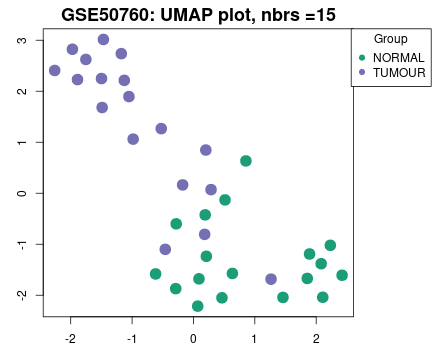

Supplement: Supplementary file 10 [file DataSheet2.ZIP › Zip S5/GSE50760/NORMAL vs TUMOUR/GSE50760.UMAPPLOT.jfif]

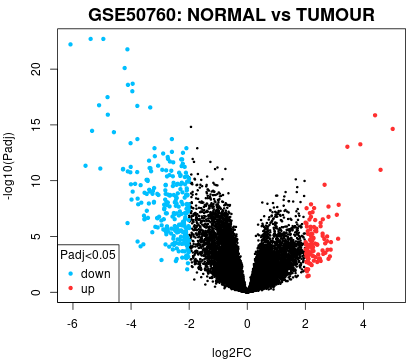

Supplement: Supplementary file 10 [file DataSheet2.ZIP › Zip S5/GSE50760/NORMAL vs TUMOUR/GSE50760.VOLCANOPLOT.jfif]

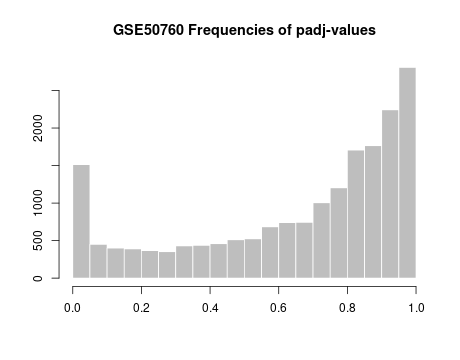

Supplement: Supplementary file 10 [file DataSheet2.ZIP › Zip S5/GSE50760/TUMOUR vs METASTASIS/GSE50760.ADJ.PVALUEPLOT.jfif]

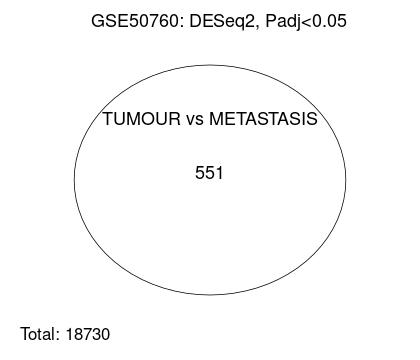

Supplement: Supplementary file 10 [file DataSheet2.ZIP › Zip S5/GSE50760/TUMOUR vs METASTASIS/GSE50760.LIMMAPLOT.jfif]

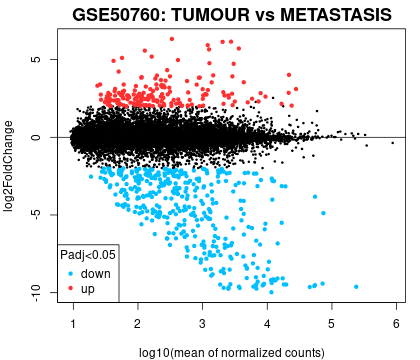

Supplement: Supplementary file 10 [file DataSheet2.ZIP › Zip S5/GSE50760/TUMOUR vs METASTASIS/GSE50760.MAPLOT.jfif]

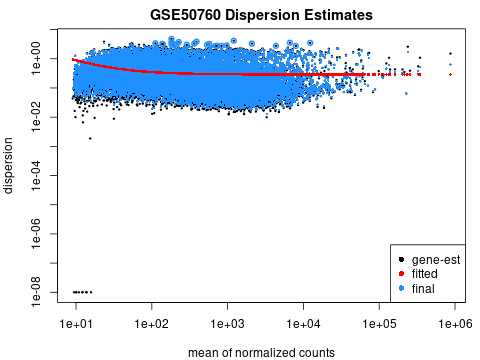

Supplement: Supplementary file 10 [file DataSheet2.ZIP › Zip S5/GSE50760/TUMOUR vs METASTASIS/GSE50760.MEAN-VAR.PLOT.jfif]

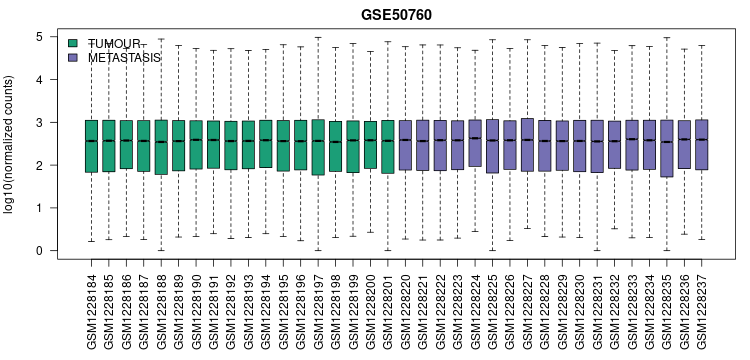

Supplement: Supplementary file 10 [file DataSheet2.ZIP › Zip S5/GSE50760/TUMOUR vs METASTASIS/GSE50760.SAMPLEDIST.PLOT.jfif]

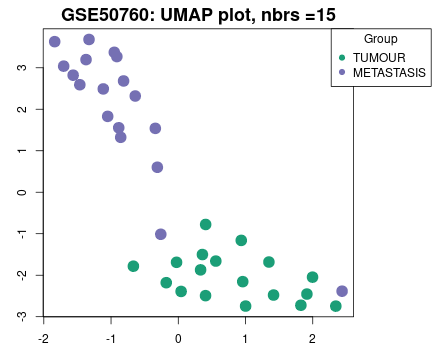

Supplement: Supplementary file 10 [file DataSheet2.ZIP › Zip S5/GSE50760/TUMOUR vs METASTASIS/GSE50760.UMAPPLOT.jfif]

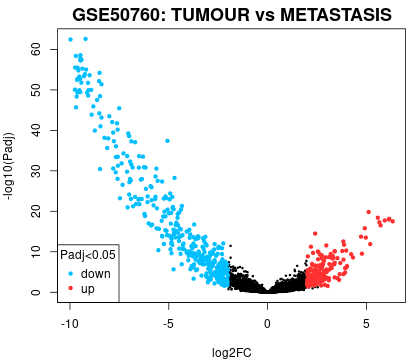

Supplement: Supplementary file 10 [file DataSheet2.ZIP › Zip S5/GSE50760/TUMOUR vs METASTASIS/GSE50760.VOLCANOPLOT.jfif]

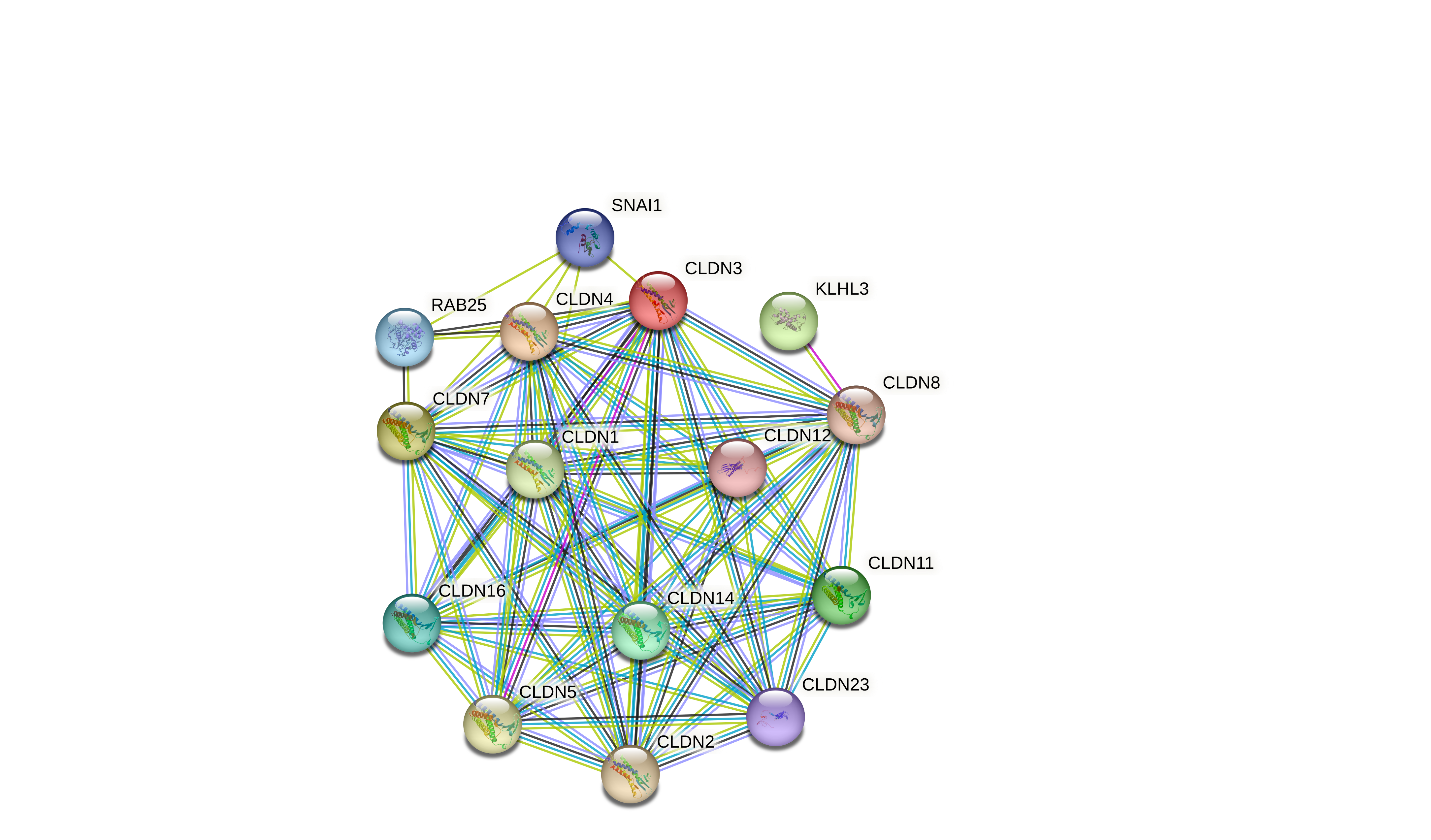

Supplement: Supplementary file 10 [file DataSheet2.ZIP › Zip S5/STRING CLAUDINS/string_hires_image.png]

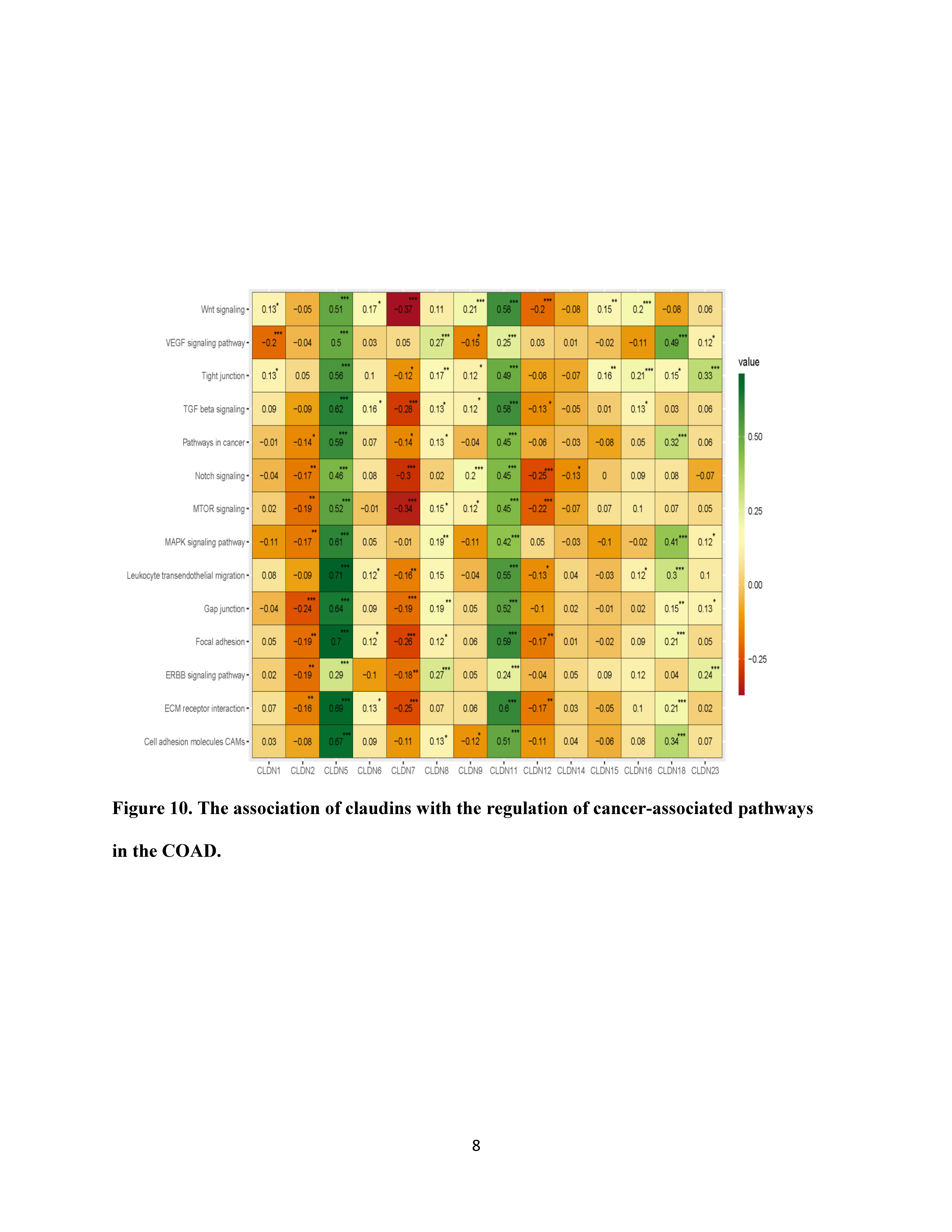

Supplement: Supplementary file 11 [file Figure10.TIFF]

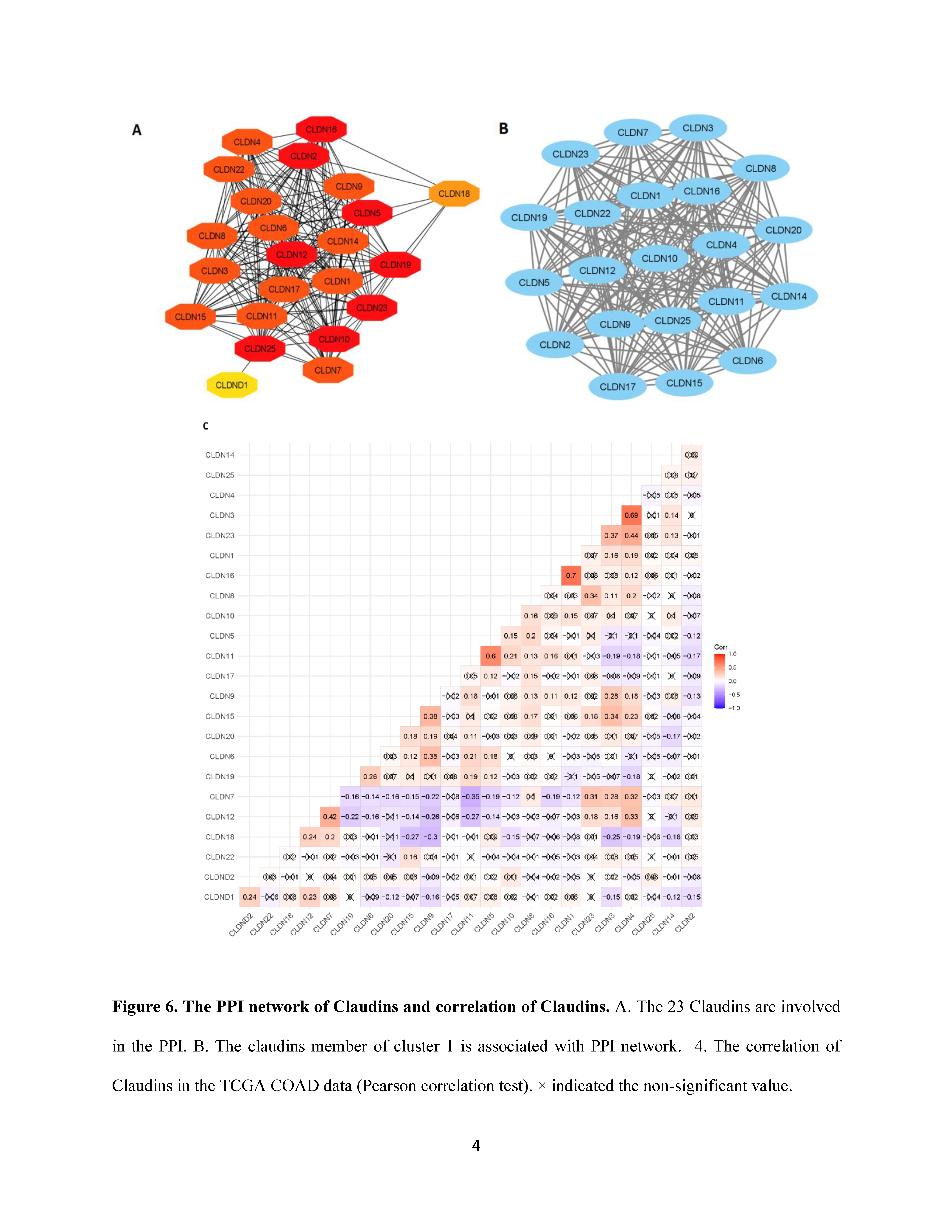

Supplement: Supplementary file 13 [file Figure6.TIFF]

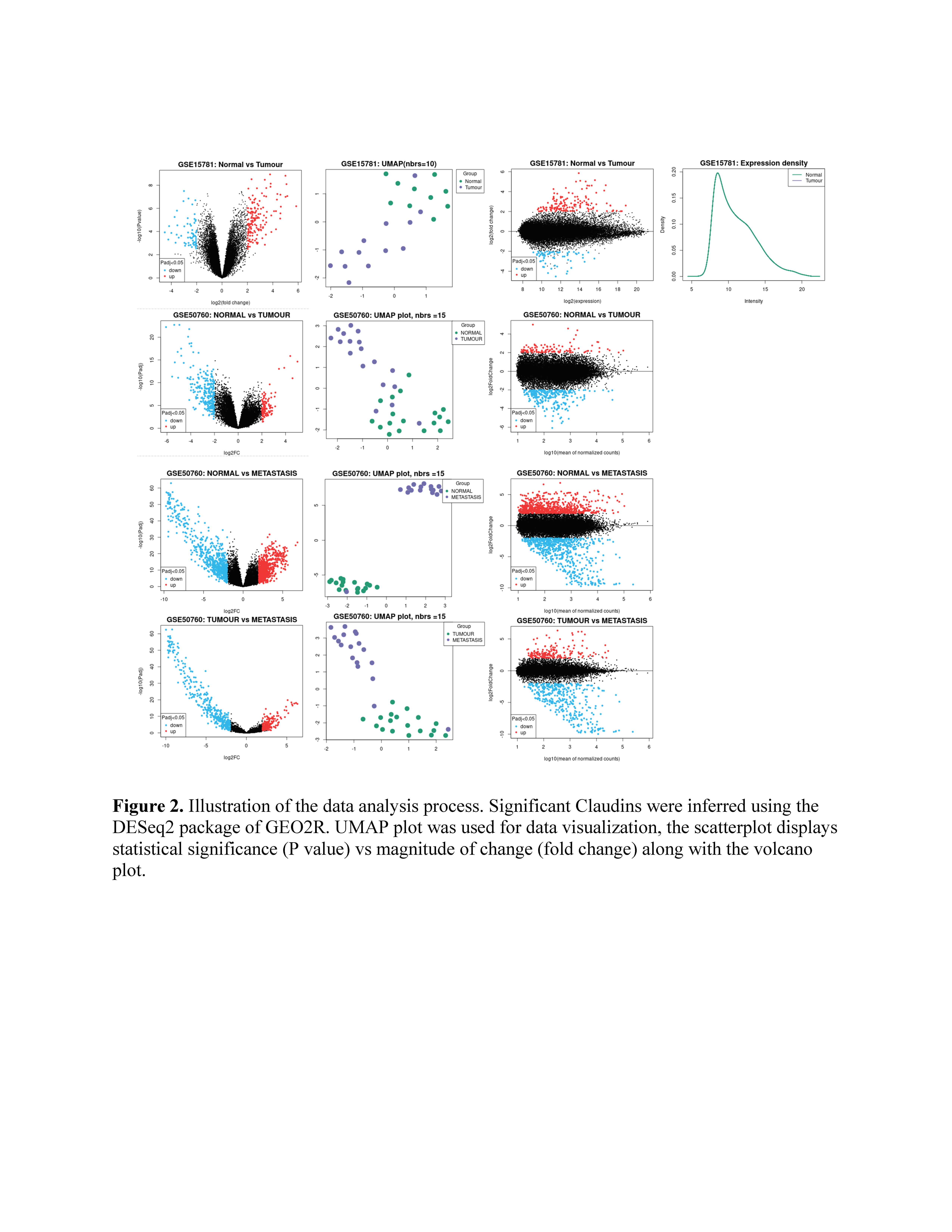

Supplement: Supplementary file 14 [file Figure2.TIFF]

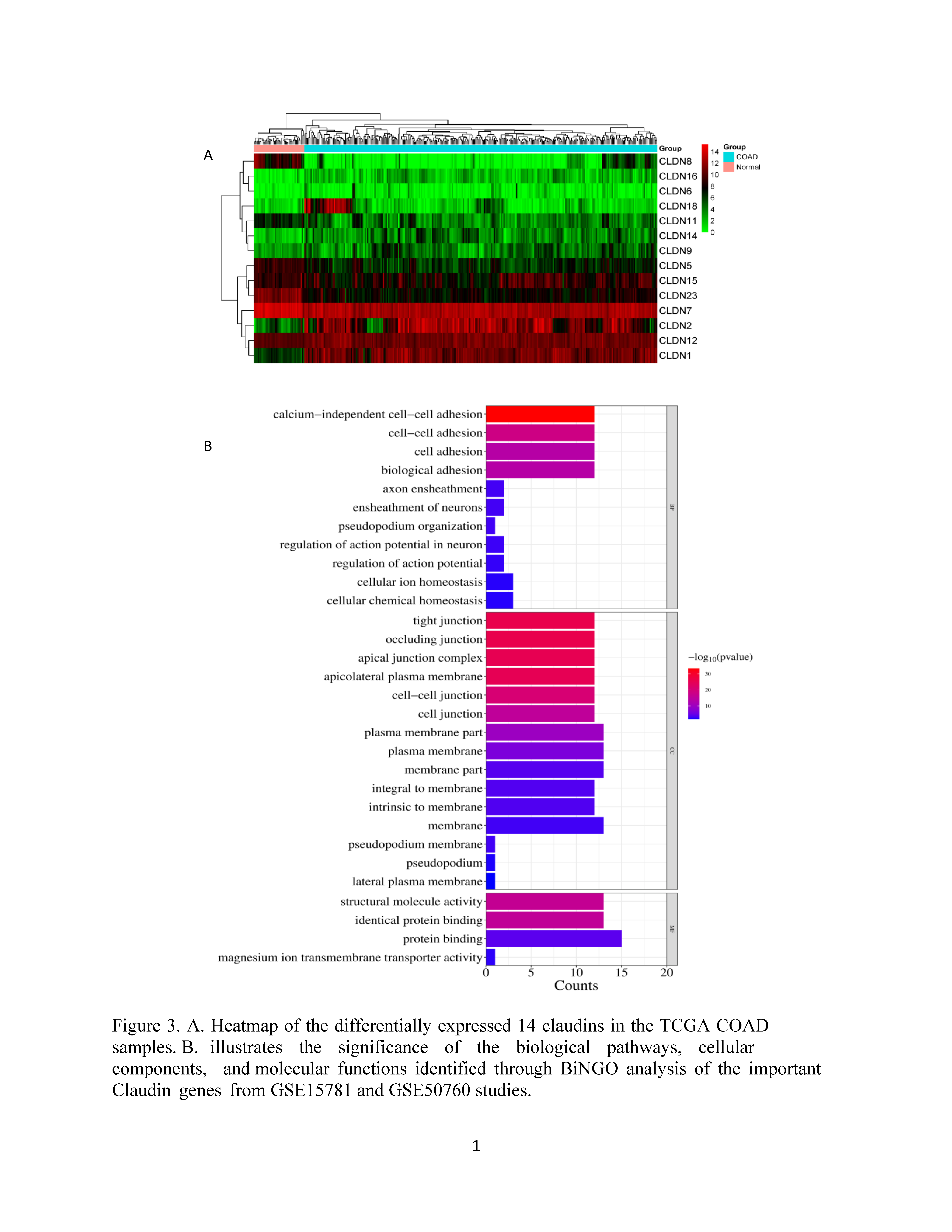

Supplement: Supplementary file 15 [file Figure3.TIFF]

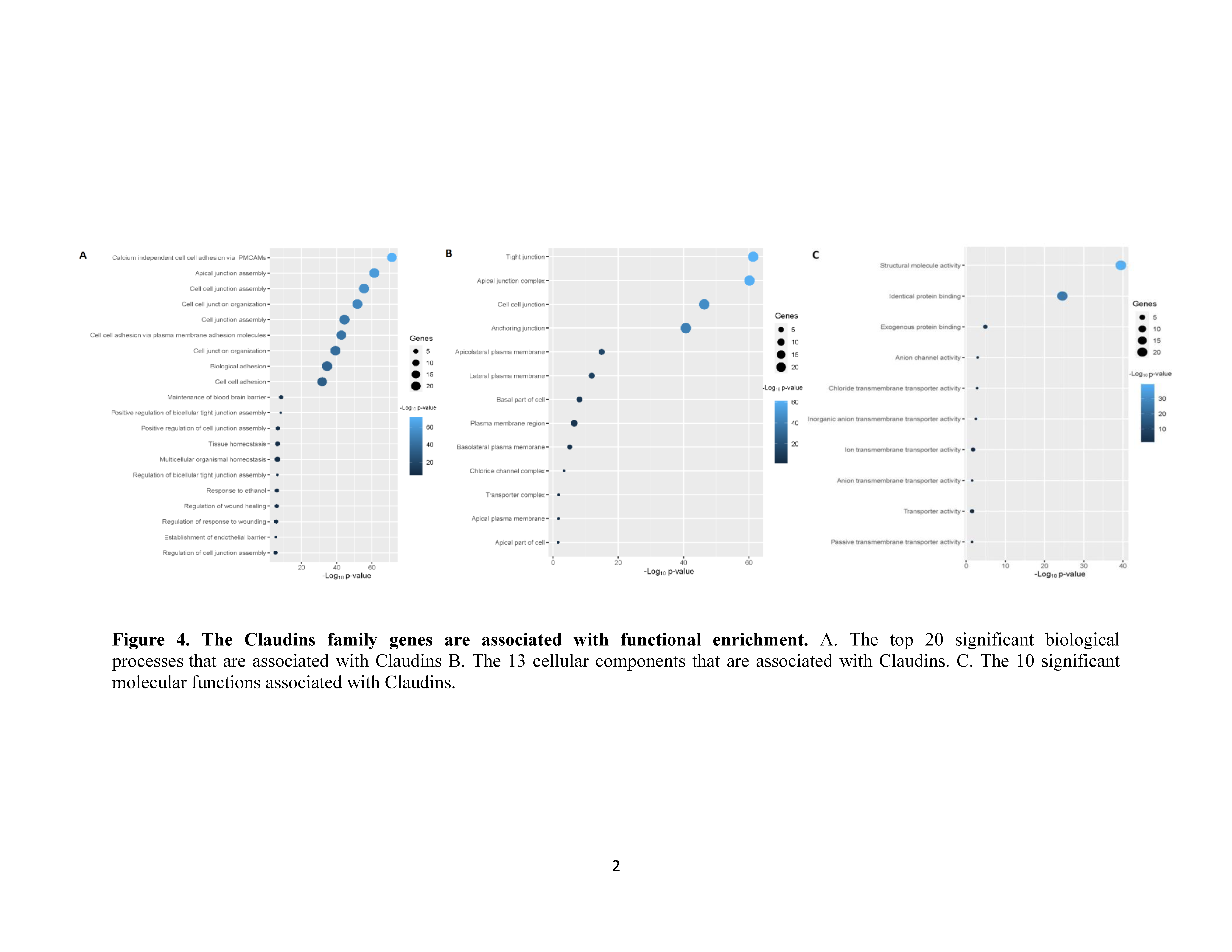

Supplement: Supplementary file 16 [file Figure4.TIFF]

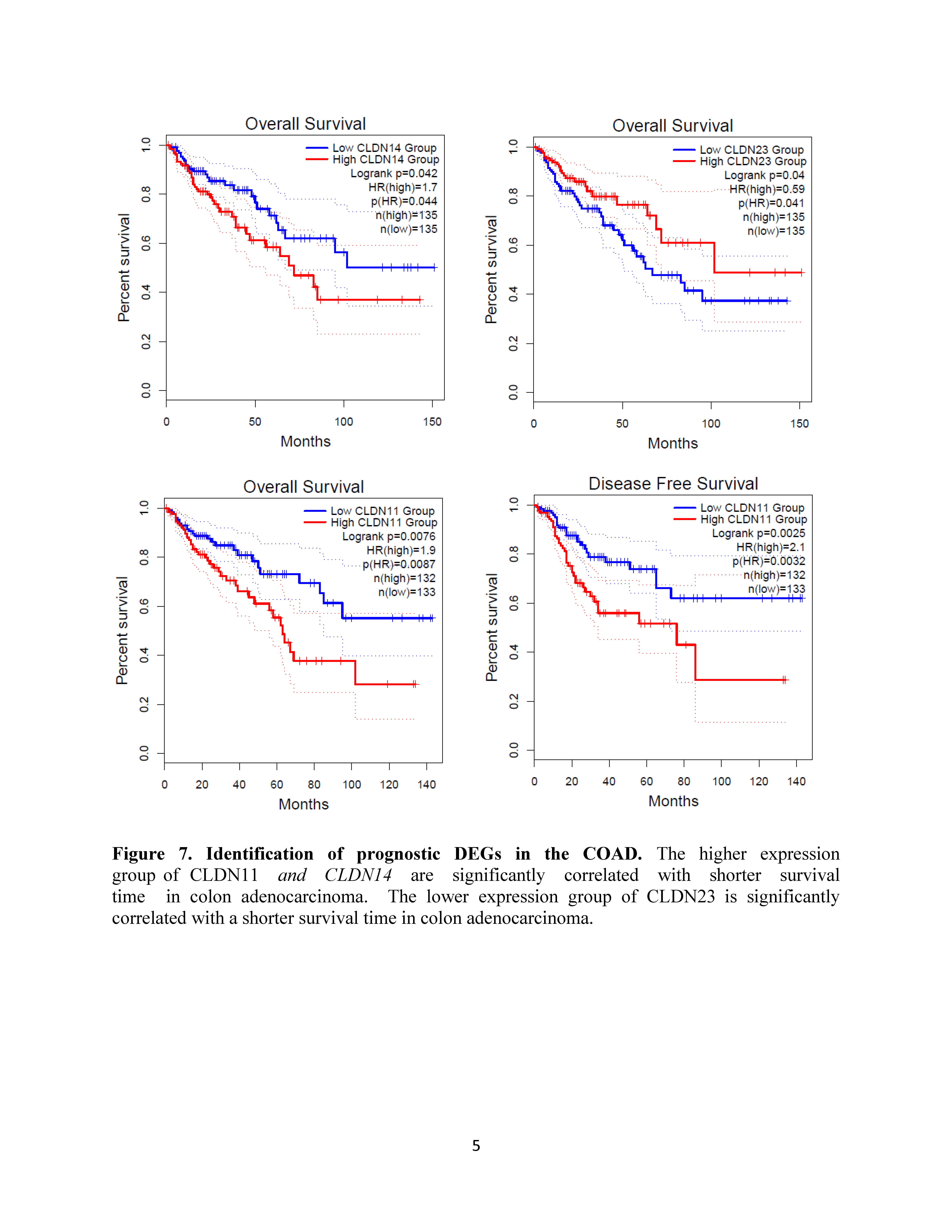

Supplement: Supplementary file 17 [file Figure7.TIFF]
