# Supplementary material for: Identification of key claudin genes associated with survival prognosis and diagnosis in colon cancer through integrated bioinformatic analysis
Source: Front Genet. 2023 Sep 19;14:1221815. doi: 10.3389/fgene.2023.1221815 (PMC10550083; doi:10.3389/fgene.2023.1221815)
Supplement: Supplementary file 9 [file DataSheet1.PDF]

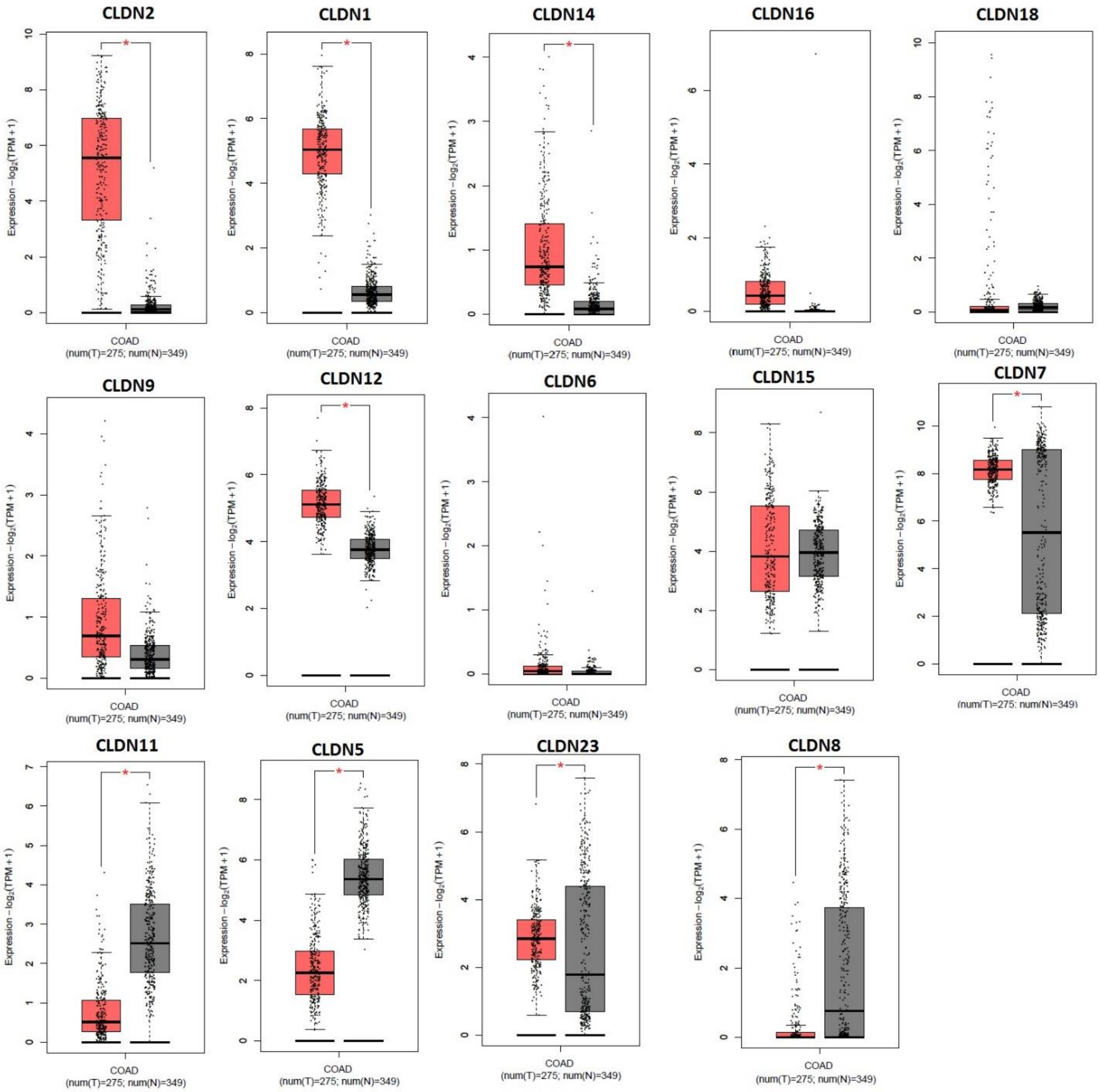

Supplementary Figure S1. The expression differences of claudins in the TCGA tumor samples with combined GTEx normal data and TCGA normal data.* indicated that the adjusted p-value<0.05 and Log2FC>0.50.
